# Knowledge about handling hazardous materials as factors associated with adherence to healthcare waste management practices among waste handlers at government district hospitals of Madhesh province, Nepal: A quantitative-qualitative methods study

**DOI:** 10.1371/journal.pgph.0002028

**Published:** 2024-12-05

**Authors:** Jot Narayan Patel, Shambhu Kumar Upadhyay, Ajay Rajbhandari, Rabindra Bhandari, Anil Poudyal

**Affiliations:** 1 Nepal Health Research Council, Kathmandu, Nepal; 2 Patan Academy of Health Science, Lalitpur, Nepal; 3 Helen Keller International, Nepal; 4 Public Health Concern Trust, Nepal; University of Groningen, NETHERLANDS, KINGDOM OF THE

## Abstract

Healthcare Waste (HCW) is a special waste produced in healthcare institutions, including hospitals. It has a high potential for infection and injuries. The issue of waste disposal is growing as the number of hospitals, clinics, and diagnostic laboratories in Nepal continues to increase. The study aimed to assess the adherence to healthcare waste management (HCWM) practices and knowledge among waste handlers at the government district hospitals of Madhesh Province of Nepal which lies in the southern part of the country. A cross-sectional mixed-method study design was employed to assess the adherence of healthcare waste management practices and knowledge of healthcare waste management guideline among HCW handlers from 10 district-level hospitals in Madhesh Province. We developed a semi-structured questionnaire from Nepal’s National HCWM Guideline 2014 and the World Health Organization HCWM Rapid Assessment Tool 2011, to interview 60 HCW handlers for quantitative information. Then 10 key informant interviews were conducted using KII guidelines with related stakeholders of district hospitals of Madhesh Province. A four-point Likert scale was used to assess the practices and knowledge of HCW handlers and health facility-related factors. Descriptive data analysis was presented in tables for frequency, percentage, mean, and standard deviation, and correlation was presented in Graphs. A thematic analysis was performed for qualitative data by using RQDA and discussing the findings before concluding the study. Among the sixty participants, the median age was thirty-five years while thirty percent were less than the median age. Among total participants, the majority of female were 65% and almost all of them (96.67%) were married. The majority (65%) were females and almost all (96.67%) were married. About one-third (36.67%) of participants were illiterate. Most of the participants had experience of 5 to 10 years. The mean adherence of HCWM was 74.88±9.66 SD. Among the participants, half of them had adequate knowledge while the median knowledge of HCWM was 39 and the inter-quartile range was 5 (q3 = 41, q1 = 36). The mean of the HCWM practice was 24.18±5.96. The median of health facilities-related factors was 13 and the interquartile range was 3 (q3 = 15 and q1 = 12). The full adherence to HCWM guideline 2014 was extremely low among healthcare waste handlers. The HCWs had less adequate knowledge of HCWM and they did not practice to manage HCW adequately in district hospitals. However, the hospitals had adequately provided amenities to manage healthcare waste.

## Introduction

HCW is the term encompassing all waste generated by healthcare facilities, laboratories, and research facilities. According to the World Health Organization, of the total amount of waste generated by healthcare activities, about 15% is considered hazardous material as it tends to be infectious, toxic, or radioactive [1, 2].

A study conducted in South Africa showed that around 90.4% of cleaning staffs were trained on HCWM in the academic hospital in Pretoria. Around 64% of participants knew the theories, legislation, and guidelines of HCWM [3]. The major causes that create a problem in HCWM are interim storage (50%), fatigue (25%), lack of supply (10%), segregation (7.5%), sharp disposal (6%), and timeliness/training (1.5%) [4]. In Uganda, Full adherence to HCWM among healthcare workers was 10.5% and 85% were partially adhered [5]. The self-reported questionnaire method is one of the easy method to measure the adherence. It need a cut-off point to measure the adherence [6]. A study among healthcare providers and cleaning staffs reported that the majority (78%) had received training for waste management and 52% of them had trained twice a year. Likewise, 47% of respondents were working for 11–15 years. Only 52.0% of the respondents knew about the bio-medical waste management process in the hospital. Similarly, 37% knew that microwaves can be used to handle bio-medical waste management. However, 66.0% were aware of the universal precaution rule for bio-medical waste management [7]. It was also reported that 53.0% used color coding to dispose of infectious waste, 55.5% segregated general waste from Bio-medical waste, and 46.5% collected bio-medical waste and transported to the designated storage site. About 66.5% used personal protective equipment when handling bio-medical wastes [7]. During waste handling 30.0% of the participants had previous exposure to the risk/hazards, 20% had been pricked with needles, 7.0% who had been exposed to infectious and 3.0% had come into contact with blood [3]. It was observed that segregation of waste at the generation point was not properly followed and hospitals staffs were not fully aware of proper segregation at the point of generation and collection. The waste was being collected in shopping bags/bins, once filled (56.3%) carried out by waste handlers (50%) to temporary storage point. It also revealed that 50% hospital had temporary storage area within the hospital premises with color coded labeled containers (50%) for waste segregation, rest of the hospitals dumped waste in open area [8]. A study also reported that the sanitary staffs had low knowledge of healthcare waste management. All the sanitary staffs did not know about the management of medical waste except they had correct knowledge about the definition of waste management and the hazards of exposure to infectious waste [9]. A waste segregation was 56.2% in Uganda shown by a study that was below WHO recommended standards of adherence (80%) and 44.4% of healthcare waste generated before disposal. It was also lower percentage of WHO recommended standards [5]. A study reported that 20% housekeeping staffs had poor knowledge on HCWM and 58% were following a moderate level of practicing Bio-medical waste disposal methods [10]. HCW is becoming a global public health concern, particularly in Low and Middle-Income Countries (LMICs) [11]. In LMICs like Nepal, studies on the level of safe HCWM practices in healthcare facilities are limited. HCWM training is essential to HCW handlers that help to improve their knowledge and practice. In big hospitals in the western part of Nepal, only 9% were found to be labeled and 16% of bins were cleaned in hospitals. In clinics, 19% of the bins were labeled and 33% were cleaned. Only cardboard, plastic bottles, and metals were being sent to recycling during the study period [12]. The hospital staffs generally connected healthcare waste with the transmission of hospital-acquired infections. A good number of diseases (HIV, jaundice (hepatitis), diarrhea, TB, etc) had transmitted through healthcare waste [13].

The presence of raised concentrations of lead and chlorine seen in the site of hospital waste dump. Lead is toxic elements and chlorine in particular is related to dioxin elements and furans emission in the environment [14]. Major heavy metal present in soil after contamination of HCW are Mercury, lead, Zink, Chromium, Cadmium, Coper, and Silver. Hg and Zn were above critical levels between 20 and 80 m while Pb and Cd level were above critical levels between 20 and 40 m. These were determined from the incinerator at given distance [15]. The source of these heavy metal are thermometer, blood pressure cuffs, laboratory chemicals, plastics, syringes etc [16].

The most prevalent antibacterial resistance bacteria are *E*.*coli* and *Klebsiella spp*. And it depended on the distance of sample collection from the hospital drainage essential point for liquid hospital waste. The number of bacteria was found to decrease with the increase in distance and it was reported that the highest number of isolates were resistant to Amoxicillin (75%). *Salmonella spp*. were 100% resistant to Ampicillin, followed by *E*.*coli* (75%), *p*. *vulgaris* (75%), *klebsiella spp* (68.7%) and *Enterobacter spp*.53.6%). Among all isolates of Gramm-negative bacteria, 75% of were found to be resistant to Amoxicillin, followed by Ampicillin (63.5%), Chloramphenicol (30.7%), Nitrofurantoin (26.9%), Gentamycin (28.8%), and least Ciprofloxacin 23.1%) [17]. The common species of bacteria present in leachate of HCW are *Staphylococcus felis* (64.4%), *Staphylococcus sciuri* (26.0%), *Staphylococcus epidermidis* (2.7%), *Staphylococcus warneri* (2.7%), *Staphylococcus lentus* (1.4%), *Staphylococcus saprophyticus* (1.4%), and *Staphylococcus haemolyticus* (1.4%) [18]. Penicillin was the least effective drug, i.e. resistance rate of 60.3%, followed by erythromycin (39.8%), azithromycin (28.8%), and oxacillin (16.5%). However, Vancomycin, levofloxacin, and gentamicin were the most effective antimicrobials [18]. Further, the wastewater and waste dump soil isolates were all resistant to Amoxicillin, Tetracycline, Gentamycin, Erythromycin, and chloramphenicol. It was also reported that wastewater isolates, 4 (66,7%) were sensitive to Zinnacef, 3 (50%) to Nitrofurantoin and Pefloxacin, and 2 (33.3%) were sensitive to Ciprofloxacin [19].

**Fig 1:**
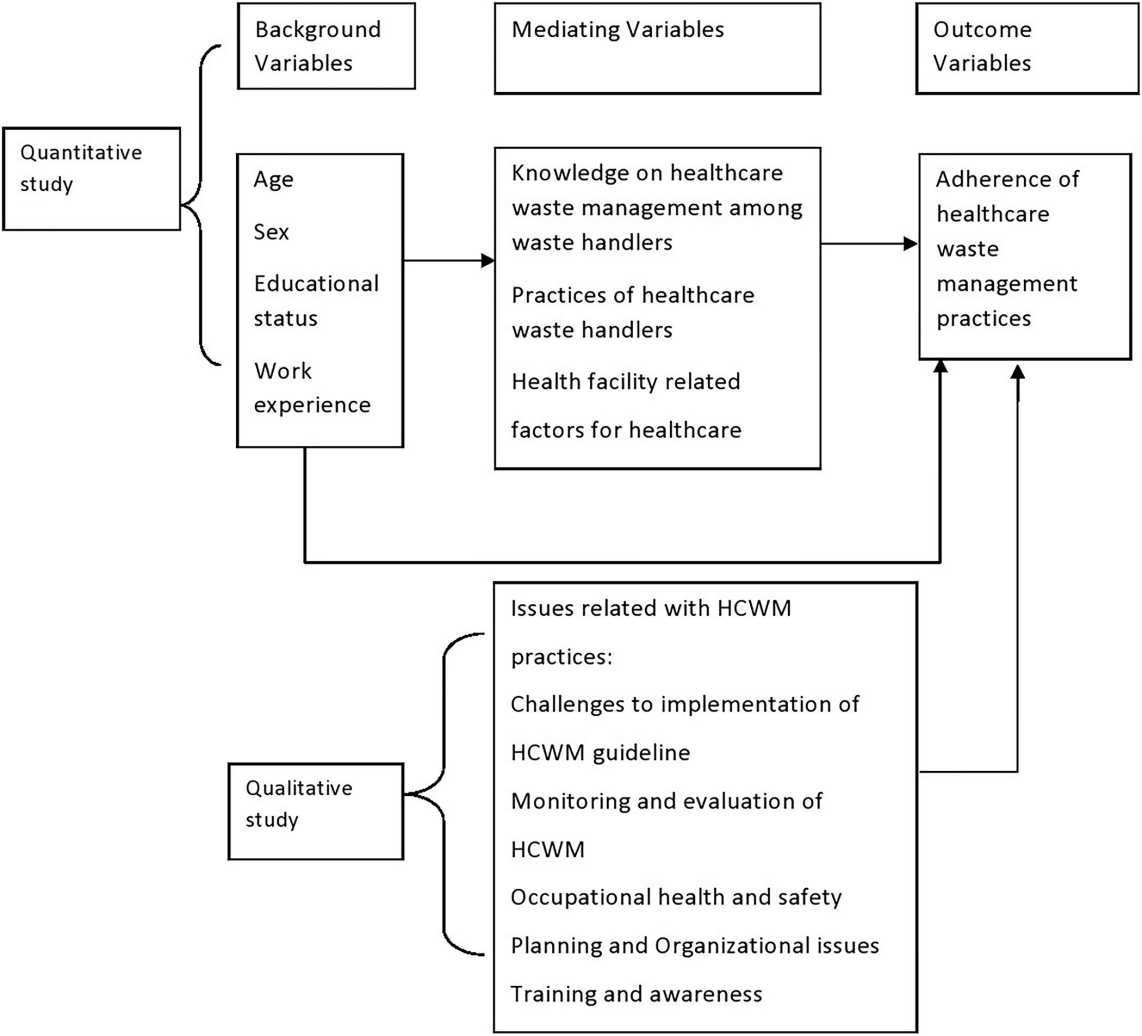
Conceptual framework.

A study from Lucknow (India) reported that the knowledge of color coding and segregation of bio-medical waste at source was much lower (0.0% & 4.6% only) among cleaning and maintenance staffs of hospitals. Only one out of twenty-two cleaning and maintenance could correctly identify the symbol of bio-hazards. Cleaning and maintenance staffs were least concerned about reporting of bio-medical waste system in case of noncompliance with the guidelines [20]. With the rapid rise in several healthcare institutions in Nepal, the number of medical waste is also increasing. For the safe and scientific management of biomedical waste, several crucial steps need to be followed by healthcare institutions. These include proper waste handling, segregation, mutilation, disinfection, storage, transportation, and finally disposal [21]. A range of initiatives have been pursued by various governmental and non-governmental institutions in Nepal. Those initiatives include the Solid Waste Management Act of 2011 [22], guideline 2014 [23] to address the waste challenge in Nepal. HCWM is a part of hospital hygiene and environment maintenance activities. Only bio-medical waste is hazardous; when hazardous waste is not segregated and mixed with non-hazardous waste, then it becomes 100% hazardous [24]. It is vital to make the whole hospital clean and in a satisfactory state of hygiene [24]. However, the compliance with HCW management observed has not been consistent with Nepal’s recommended guidelines. Till the commencement of the study, researchers could not find any information available to describe the scenario of actual HCW handling practice in the government health institutions of Madhesh Province. Therefore, the present study was conducted to assess the adherence to HCWM practices among HCW handlers of all government district hospitals in Madhesh Province as well as to assess the level of knowledge and practice of HCW handlers. This will identify the gap in knowledge and practice of the HCW handlers and adherence to the HCWM guidelines. Identifying the issues will support the hospital management team and related authorities to improve the HCWM practice adequately adhering to the guidelines. A conceptual framework was adapted from a study conducted in Colombo in 2017 on an analysis of medical waste management practices in the healthcare sector in Colombo [25], as depicted in Fig 1.

## Methods

### Study design, setting, and participants

We employed a hospital-based cross-sectional quantitative-qualitative method study design among the government district hospitals in Madhesh Province of Nepal. This province is in the southern part of the country and is characterized as a plain region, being the second most densely populated. At the time of the study, the total population of Madhesh Province was 5,918,972 based on census 2011. This province comprises eight districts, from Saptari in the east to Parsa in the west. The predominant languages spoken here are Maithili, Bhojpuri, Bajjika, and Nepali. It has 136 local government bodies including; 1 metropolitan city, 2 sub-metropolitan cities, 73 municipalities, and 59 rural municipalities. The estimated growth rate of the Gross domestic product (GDP) of Madhesh Province was 2.3% for 2019/2020 [26].

Regarding health structures in the province; there are altogether 790 government health facilities, among them 742 are health posts, 35 are primary healthcare centers, 10 district-level hospitals at the time of the study, and 2 health Academy and one referral-level hospitals (specialized hospitals) [27]. Besides, there are 80 private hospitals and two private medical colleges in the province. There are different sanctioned posts for health workers and other support staffs in each district hospital. The total sanctioned post of staff for ten district hospitals of the province was two hundred forty-two (242) which includes medical superintendents, Specialists, Medical Officers, Nursing Officers, staff nurses, Auxiliary Nurse Midwives (ANMs), Paramedics, Administrative staff, and support staffs during the study time. However, according to a new organogram of district hospitals, there is no sanctioned post for HCW handlers.

The Health Service Act was endorsed in 1996 based on the National Health Policy of 1991 and was amended in 2018. Since then, the government has given the authority to hire the HCW handlers either on a contract or wage basis i.e., per day payment through the hospital operating committee or hospital management committee rather than the permanent service entry system. They hire the required workforce and manage them with the income from the hospital operating committee [28–30]. The hiring of temporary staffs is not a sustainable way for HCWM due to the frequent turnover of trained HCW handlers. Repeated hiring of new HCW handlers need orientation and training before involving HCWM in a health institution. A short training course of HCWM is provided by the management division of the Department of Health Service to Quality in charge of hospital i.e. registered nursing staffs.

We used a complete enumeration method to collect the data from ten district government hospitals of Madhesh Province, Nepal. At the time of the study three hundred sixty-seven (367) staffs, including 63 HCW handlers, were working in the ten hospitals.

The study population comprised all 63 healthcare waste (HCW) handlers who met the inclusion criteria. Each HCW handler was approached for participation; however, three individuals were on leave during the study period. The data collection tools were prepared in English then it was translated into Nepali. The Nepali questionnaires were back-translated into English. After that, the questions were matched with the previous English tool. Only matched questionnaires were administered for data collection. The Nepali Language was used during the interview. Many of them understood the Nepali Language well and in case of confusion the researcher himself spoke in participants’ local language. The local languages were Maithali and Bhojapuri. The researcher himself was from Madhesh Province and his mother language was Bhojpuri and also fluent in Maithili. A researcher explained information in front of witnesses and participants, and then a figure print was taken from the participants for participation.

According to the HCWM guideline 2014 AD; each hospital has an HCWM committee that includes the Chiefs/Directors of health facility, Department Head, Matron, Waste Management Officer, and Representative from supportive staffs. Medical Superintendents are mostly available in District hospitals and act as authoritative and regulatory bodies for the HCWM. So, one KII was done with the medical superintendent from each hospital giving a total of 10 KII. Verbal consent was sought from all the participants due to the COVID-19 lockdown during the data collection period. Obtaining consent over the phone ensured participant safety while adhering to ethical standards during these challenging circumstances.

### Study variables

#### Background variables

Age: Participants’ age was more than 18 years. Sex: both male and female genders were included in the study, educational status, marital status, and work experience: Healthcare waste handlers had been working at the hospital for more than six months.

#### Mediating variables

*A*. *Knowledge of HCWM*. Knowledge of HCW handlers towards healthcare waste management was a mediating variable.

Sixteen items (4-point Likert scale) on knowledge of HCWM were used [31]. The score of knowledge of HCW handlers did not follow the normal distribution; the median score of knowledge was 39.5, IQR = 4.5 (41–36.5 = 4.5). The median was taken as a cut-off value for knowledge of HCWM. More than or equal to the median was taken as adequate knowledge of HCWM and less than the median was taken as inadequate knowledge of HCWM [32].

*B*. *Practice of HCWM*. Practices of HCWM by HCW handlers were also taken as a mediating variable in this study.

Fifteen items (4-point Likert scale) on the practice of HCWM were used. The practice score of HCW handlers followed the normal distribution; thus, the mean was obtained as the cut-off value. The mean of practice items was found to be 24.18±5.96 SD. Equal to or more than the mean was taken as an adequate practice and less than the mean was taken as an inadequate practice performed by HCW handlers on HCWM [33].

*C*. *Availability of related health-facility-related factors on HCWM*. The health facility (Hospital) related factors (supplies and availability) were also taken as a mediating variable in this study.

Eight items (4-point Likert scale) on the availability of health-facility-related factors for HCWM were used. The data did not follow the normal distribution. Thus, the median was obtained as the cutoff value for assessing the level of availability of health facilities-related factors which was 13, IQR = 3 (15–12 = 3). More than or equal to the median was taken as adequate while less than the median was taken as inadequate availability [33].

### Outcome variable

#### Adherence to HCWM guideline

Adherence to Healthcare Waste Management Guideline 2014, Nepal was the outcome variable of the study.

Adherence to HCWM guidelines is the adoption of practices to handle healthcare waste, knowing healthcare waste, and the availability of health facility-related factors to manage healthcare waste at health institutions according to HCWM guidelines. The maximum average score of knowledge, practice, and health facility-related factors by the HCW handlers was 117 (100%). The score of 80% or more by the HCW handler was taken as full adherence while less than 80% was taken as partial adherence [5, 34].

According to the WHO, the minimum value required for full adherence to the HCWM guidelines is 80%, as presented in Table 1 [5].

**Table 1:** Category of adherence to HCWM.

SN	Level of adherence	Score (Percentage)
**1**	Full adherence	≥ 93.6 (≥80%)
**2**	Partial adherence	> 0 to 93.5 (0%-80%)

### Tools and techniques for data collection

A semi-structured questionnaire was used to assess knowledge, practices, and health facility-related factors for adherence to the HCWM guideline 2014 among HCW handlers. And an open-ended interview guideline was used to explore in-depth information from Key persons and stakeholders related to HCW of 10 government district hospitals. The data collection period was from November to December 2020.

Questionnaires were adapted from the literature review, 5 items for knowledge, 6 items for studies for practice, and 1 item for health facilities-related amenities required for HCWM were taken from a similar study of a low-income setting country. In addition; 4 items for knowledge, 4 items for practice and 4 items for the availability of amenities required for HCWM on health facilities were adapted from HCWM guideline 2014, department of Health Service, Nepal, and likewise, 3 items for knowledge, 3 items for practice and 1item for health facility related amenities required for HCWM were taken from HCWM rapid assessment tool of WHO; Additionally, 4 items for knowledge, 3 items for practices, 3 items for health facilities related amenities required for HCWM were taken from a study conducted by Nepal Health Research Council [23, 35–37]. After that different sets of questionnaires were prepared to measure the Knowledge [31] and practice of the waste handlers and amenities required for HCW management.

Knowledge questions were structured on a 4-point Likert scale, strongly agree = 3, agree = 2, disagree = 1, and strongly disagree = 0. Practice questions were structured on a 4-point Likert scale of never = 0, rarely = 1, often = 2, and always = 3 [7].

### Data management and analysis

The researcher assigned codes to the questionnaire and entered them on the pre-defined data entry form in Epi-Info 7 software developed by CDC, USA. Entered data were then exported as a Microsoft Excel spreadsheet and cleaned data in Excel format were imported and analyzed using STATA MP Version 13. A descriptive statistic was used to analyze quantitative data for frequency, percentage, and mean/median of the study variables. The level of knowledge, practice, and health facility-related factors of HCW handlers for HCWM were assessed. Similarly, qualitative data was collected from the key informants, transcribed in Nepali, and translated into English language. The translated data was transported to RQDA software for analysis. All the transcription was repeatedly reviewed and using the added option of RQDA the pieces of code were highlighted in RQDA. After that, the themes were generated according to the deductive method. After that the HTML file was generated (S1 Text); the description of the code according to the suitable theme and qualitative finding was generated.

A six-step method was used to analyze the qualitative data. The transcription was read three times by the researcher, to understand the meaning of the sentences and paraphrase. After that quotations were selected. After the revision of selected quotations, the keywords were decided for the code. Codes were organized into meaningful groups to identify patterns and relationships, getting inside into the research question and developing a theme. The themes were conceptualized and used RQDA to develop a conceptual model [38]. The primary codes were generated by researchers and coded by another independent researcher. The percentage agreement method was used to calculate the inter-coder reliability. The percentage agreement methods were calculated from the number of concordant responses divided by the total codes generated by both researchers [39]. Total codes generated by the first researcher were 61 and by second researcher were 51. Among them 45 codes were similar. The total codes generated by both researchers were 61, including matched codes (45). The inter-coder reliability score was 74.13%. The member checking was done for KIIs. All 10 interview transcriptions along with records were sent to participants for reviewing any difference between transcription and recording. The suggested feedback was incorporated into the transcriptions.

### Validity and reliability of tools

The data collection tool was adapted from a report on the assessment of biomedical waste management practice among healthcare institutions by the Nepal Health Research Council (NHRC) (2012-2-13) [36] and the HCWM rapid assessment tool (WHO), [37] as well as from similar research carried out in low-income countries [33]. After that tools were prepared in English first and then, finalized in Nepali language after discussion with an expert, guide (Prof. Dr. Shambhu Kumar Upadhyay-Patan Academy of Health Science), and co-guide (Associate Prof. Ajay Rajbhandari). The pilot study was conducted in a similar setting on the district hospital of Koshi province among 10 participants from two hospitals. After that, the necessary correction was done under the supervision of a guide and co-guide.

### Inclusion and exclusion criteria

Inclusion criteria: All HCW handlers of government district hospitals of Madhesh Province with at least six months of involvement in the job of waste handling [40].

Exclusion criteria: HCW handlers who were on leave or official deputation within the data collection period for two months.

### Ethical statements

Ethical approval for the study was obtained from the IRC of Patan Academy of Health Sciences (IRC-PAHS Ref: PHP2011171469). Additionally, formal approval was secured from the Provincial Health Directorate of Madhesh Province. The approval for the study was also received from the respective government district hospitals. Considering the COVID-19 lockdown, Informed consent was obtained verbally over the phone, and audio recordings were conducted. The privacy and confidentiality of the information provided by the participants were maintained by using initials during data entry and analysis. The recorded data was stored on the hard drive of the computer with coding by the name of the hospital and respondent.

## Findings

### Distribution of participants by their socio-demographic characteristics

A total of 60 participants were involved in the study. The majority of the participants belonged to the age group of more than 35 years. About 65% were female, 96.67% were married, 36.67% illiterate, and only a few (3.33%) of participants had completed higher secondary/above education. Likewise, 38.33% of participants had been working for 60 to 120 months (about 10 years) and about 26.67% had been working for more than 120 months, as presented in Table 2 (S1 Data).

**Table 2:** Distribution of socio-demographic characteristics of HCW handlers.

Variables	Frequency	Percentage (%)
Age	Median age: 35 years, interquartile range = 9 (41 to 32)	
18–25 years	2	3.33
26–30	7	11.67
31–35	9	15.00
>36 years	42	70.00
**Sex**		
Female	39	65.00
Male	21	35.00
**Current marital status**		
Never married	2	3.33
Married	58	96.67
**Education**		
Illiterate	22	36.67
No formal schooling	15	25.00
Primary school (1–5 class)	16	26.67
Secondary (6–10 class)	5	8.33
Higher Secondary (11–12 class) and above	2	3.33
**Working Period**		
6–60 months	21	35.00
61 months to 120 months	23	38.33
>121 months	16	26.67

### Scoring for knowledge, practice, and health facility-related factors for HCW management

Knowledge, practice and health facility-related factors score of respondents were continuous variables. Normality was checked before deciding the cut-off value. The median was taken as a cut-off point for knowledge; the mean was taken as the cutoff point for practices and the median was taken as a cutoff point for health facility-related factors. The result of knowledge, practice, and health facility-related factors were categorized as adequate and inadequate [33, 40].

### Distribution of participants based on knowledge of HCWM

The median score of knowledge was 39.5 (IQR = 4.5, 41–36.5 = 4.5). More than half of the participants had inadequate knowledge of HCW management.

About 68.33% of participants strongly agreed that HCW should be segregated into three different categories at the point of waste generation area and 81.67% of participants agreed that HCW must collect at least different color-coated dustbins, 56.67% of participants strongly agreed that they had closed the lid of the container during transportation. Further, 55% of participants strongly agreed that the disinfected HCW decreases the risk of disease transmission among HCW handlers and visitors in a district hospital. Likewise, 68.33% of participants strongly agreed that HCW should be stored before treatment and disposal at district hospital, and 11.67% of participants strongly disagreed that all HCWs were not hazardous in nature. About 60% of participants agreed that poor handling and disposal of HCW poses a threat to environmental effects, as presented in Table 3.

**Table 3:** Knowledge on HCWM.

Knowledge items on HCWM N = 60	Strongly disagree n (%)	Disagree n (%)	Agree n (%)	Strongly Agree n (%)
Waste generated in Hospital are HCW	8 (13.33)	7 (11.67)	45 (75.00)	0 (0.00)
Different types of waste are generated in hospital	2 (3.33)	1 (1.67)	15 (25.00)	42 (70.00)
HCW and Municipality waste are different to each other	0 (0.00)	16 (26.67)	44 (73.33)	0 (0.00)
Different color-coded dustbins should be used to segregate HCW	1 (1.67)	8 (13.33)	10 (16.67)	41 (68.33)
Different color-coded bins should be used to collect HCW	0 (0.00)	11(18.33)	49 (81.67)	0 (0.00)
The lid of waste containers should be closed during HCW transportation	11 (18.33)	3 (5.00)	12 (20.00)	34 (56.67)
Sterilization of HCW reduces the risk of disease transmission	8 (13.33)	3 (5.00)	16 (26.67)	33 (55.00)
HCW should be stored safely before treatment and disposal	1 (1.67)	0 (0.00)	18 (30.00)	41 (68.33)
All HCWs are not hazardous in nature	7 (11.67)	7 (11.67)	20 (33.33)	26 (43.33)
Non-hazardous HCWs can be reused	9 (15.00)	6 (10.00)	26 (43.33)	19 (31.67)
Bio-hazards symbols should be labeled on dustbins	2 (3.33)	1 (1.67)	11 (18.33)	46 (76.67)
HCW management, requires a special equipment	15 (25.00)	5 (8.33)	16 (26.67)	24 (40.00)
Education and training help to manage HCW	1 (1.67)	15 (25.00)	44 (73.33)	0 (0)
HCW handlers are at high risk of disease transmission	2 (3.33)	0 (0)	25 (41.67)	33 (55.00)
Poor HCWM affects environmental health	1 (1.67)	3 (5.00)	36 (60.00)	20 (33.33)
HCWM is a core standard of hospital service	2 (3.33)	6 (10.00)	29 (48.33)	23 (38.33)

The knowledge of healthcare waste was divided into two categories and found that about half of HCW handlers had adequate knowledge of HCWM and half of HCW handlers had inadequate knowledge of HCWM, as presented in Table 4.

**Table 4:** Level of knowledge of HCW handlers among HCW handlers.

SN	Level knowledge	Frequency	Percentage (%)
1	Adequate	30	50.00
2	Inadequate	30	50.00

**Fig 2:**
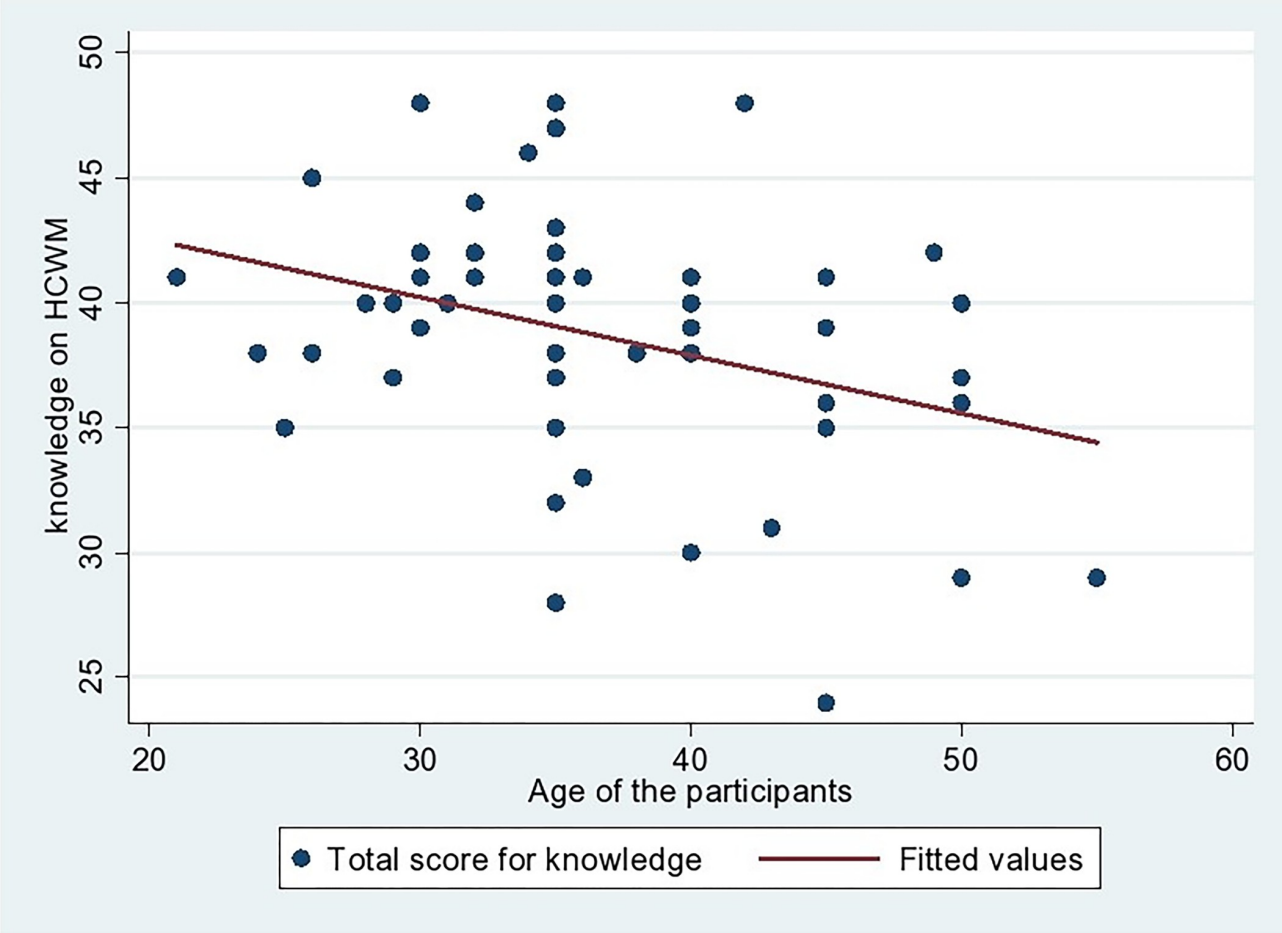
Correlation between age and knowledge of HCW handlers on HCW management.

The Pearson correlation between age and knowledge of HCW handlers on HCW management has a weak negative correlation (r = -0.345, p = 0.007), as depicted in Fig 2. It shows that the knowledge of HCW management decreased with the rise in age of HCW handlers.

### Practice of HCW management

About 46.67% of participants had performed adequate HCWM practices. Further, 60.00% of participants had never received training, 68.33% of participants had always used three color-coded dustbins correctly for waste collection and 61.67% of participants had always kept three color-coded bins in each unit. About 66.7% of them always collected HCW from waste generation sites at a fixed time, 45% of participants always removed HCW within 24 hours in the summer season, and 43.33% of participants always removed HCW within 48 hours in the winter season. All participants had never sterilized a reusable HCW in a week, and no one recorded HCW collection and disposal activities.

Further, 46% of participants often used a complete set of PPE and 16.67% of participants had sometimes used a complete set of PPE on duty hours. Similarly, 28.33% of participants always used duty gloves while handling healthcare waste. Among them, 46.67% of participants often used a boot during handling HCW and 16.67% of participants had often used an apron during handling healthcare waste. The mean of the practice of participants was 24.18 ± 0.7696 at 95% confidence interval (22.64–25.72). About 46.67% of participants had inadequate HCWM practices, as presented in Table 5.

**Table 5:** Distribution of participants based on practice of HCW management.

Practice items on HCWM	Never n (%)	Sometimes n (%)	Often n (%)	Always n (%)
HCW segregation procedure presented on wall at HCW segregation area	4 (6.67)	0 (0)	23 (38.33)	33 (55.00)
Use of color-coded dust bins to segregate waste	2 (3.33)	1 (1.67)	16 (26.67)	41 (68.33)
Use of color-coded dust bins in each unit	3 (5.00)	0 (0)	20 (33.33)	37 (61.67)
Collection of HCW at fixed time at district hospital	2(3.33)	7(11.67)	11(18.33)	40 (66.67)
Remove HCW from storage area within 24 hours in summer season	21 (35.00)	5 (8.33)	7 (11.67)	27 (45.00)
Practice to remove HCW from storage area within 48 hours in winter season	22 (36.67)	4 (6.67)	8 (13.33)	26 (43.33)
Practice to replace a sharp container (when filled 3/4 by needles)	4 (6.67)	14 (23.33)	17 (28.33)	25 (41.67)
Practice to close the lid container during HCW transportation	10 (16.67)	9 (15.00)	18 (30.00)	23 (38.33)
Practice to disinfect infectious HCW	60 (100.00)	0 (0.00)	0 (0.00)	0 (0.00)
Sterilization of reusable HCW in a week	60 (100)	0 (0.00)	0 (0.00)	0 (0.00)
Recording for waste collection and disposal	60 (100)	0 (0)	0 (0.00)	0 (0.00)
Use of Complete set of PPE on duty hour	6 (10.00)	10 (16.67)	28 (46.67)	16 (26.67)
Use of duty gloves during handling HCW	6 (10.00)	12 (20.00)	28 (46.67)	14 (23.33)
Use of boots during handling HCW	6 (10.00)	12 (20.00)	28 (46.67)	14 (23.33)
Use of apron during handling HCW	24 (40.67)	16 (26.67)	10 (16.67)	10 (16.67)
Received training or seminar on HCW management				
Yes	24 (40.00)			
No	36 (60.00)			

The practices for HCWM were also categorized into two categories and it was found that about 46.67% of HCW handlers had performed adequate handling of HCW and 53.33% of HCW handlers had performed inadequate handling of HCW, as presented in Table 6.

**Table 6:** Level of practice of HCWM among HCW handlers.

SN	Variables	Frequency (N = 60)	Percentage
1	Adequate	28	46.67%
2	Inadequate	32	53.33%

**Fig 3:**
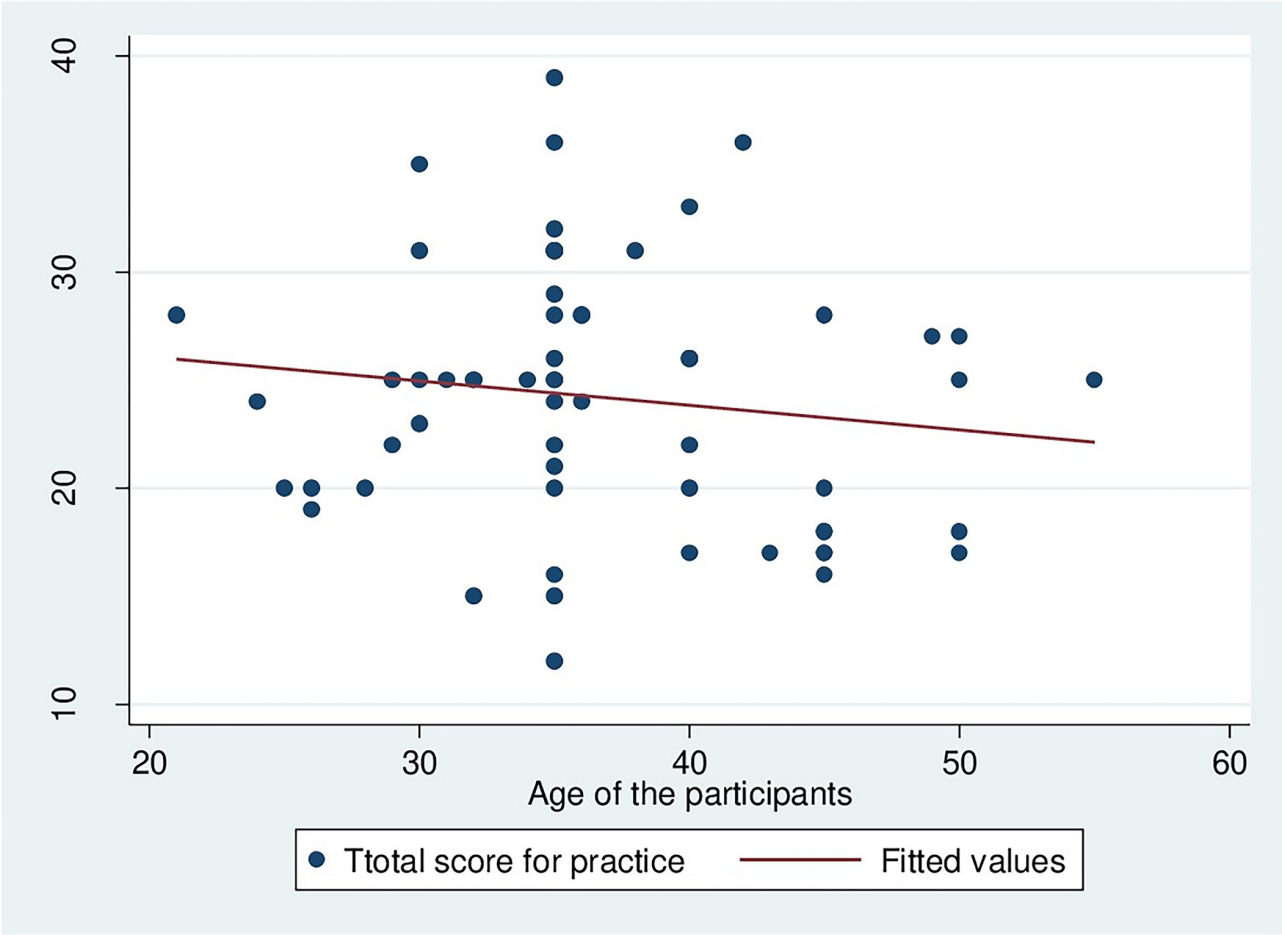
Correlation between HCW management practices and the age of HCW handlers.

The correlation between HCW management practices and the age of HCW handlers were weak negative correlation (r = -0.0137, p = 0.296), as depicted in Fig 3. It means, if the age of HCW handlers increases, the HCW management practices among them decrease.

### Health facilities-related amenities required for HCWM practice

About 71.67% of participants strongly agreed that they had PPE. Further, about 85% of participants strongly agreed that the district hospital had supplied dustbins and autoclaves for HCW collection and sterilization, respectively. About 78.33% of participants strongly agreed that the hospital had enough space to store HCW within the hospital premises. All the participants strongly disagreed that they had documented their HCWM-related activities in a district hospital. About 55% of participants agreed that there was a WCHM committee at the hospital. Likewise, 63.33% of participants strongly disagreed that there was a vaccination program for HCW handlers at district hospitals. The median of health facilities related factors was 13 (IQR = 3, 15–12). Further, 70% of participants received adequate health-facility related factors. Similarly, 65% of HCW handlers said that there was no HCWM guideline at district hospital, as presented in Table 7.

**Table 7:** Distribution of participants based on the supply by health facilities related to amenities required for HCWM.

Items on supply related factors from health-facility required for HCWM (N = 60)	Strongly Disagreed n (%)	Disagreed n (%)	Agreed n (%)	Strongly Agreed n (%)
Regular supply of PPE set to HCW handlers	0 (0.00)	6 (10.00)	11 (18.33)	43 (71.67)
Regular supply of dustbin for HCW collection	0 (0.00)	0 (0.00)	9 (15.00)	51 (85.00)
Availability of functional Autoclave to disinfect infected HCW	51 (85.00)	0 (0.00)	0 (0.00)	9 (15.00)
Availability of space for HCW storage	5 (8.33)	8 (13.33)	47 (78.33)	0 (0.00)
Regular supply of checklist to keep record of generation of HCW	60 (100.00)	0 (0.00)	0 (0.00)	0 (0.00)
Presence of active HCWM committee in hospital	22 (36.67)	0 (0.00)	33 (55.00)	5 (8.33)
The regularity of HCWM officer at district hospital for a year	20 (33.33)	0 (0.00)	29 (48.33)	11 (18.33)
Regular vaccination program for HCW handlers at hospital	38 (63.33)	0 (0.00)	16 (26.67)	6 (10.00)
Familiarity with HCWM Guidelines at district hospital				
Yes	35(58.33)			
No	25 (41.67)			

The supply of amenities required for HCWM were categorized into two categories and found that 70% HWC handlers received adequate amenities or use amenities required for HCW management. However, 30% of HCW handlers did not receive adequate amenities or did not use amenities required for HCWM, as presented in Table 8.

**Table 8:** Level of distribution of amenities required for HCWM.

SN	Level health-facilities-related factors	Frequency (N = 60)	Percentage (%)
1	Adequate	42	70.00%
2	Inadequate	18	30.00%

### Adherence to HCWM guideline

The mean adherence to HCWM guidelines among HCW handlers was 74.88 (CI 95%, 72.38–77.38), and 6.67% fully adhered to HCWM guidelines in 2014.

**Fig 4:**
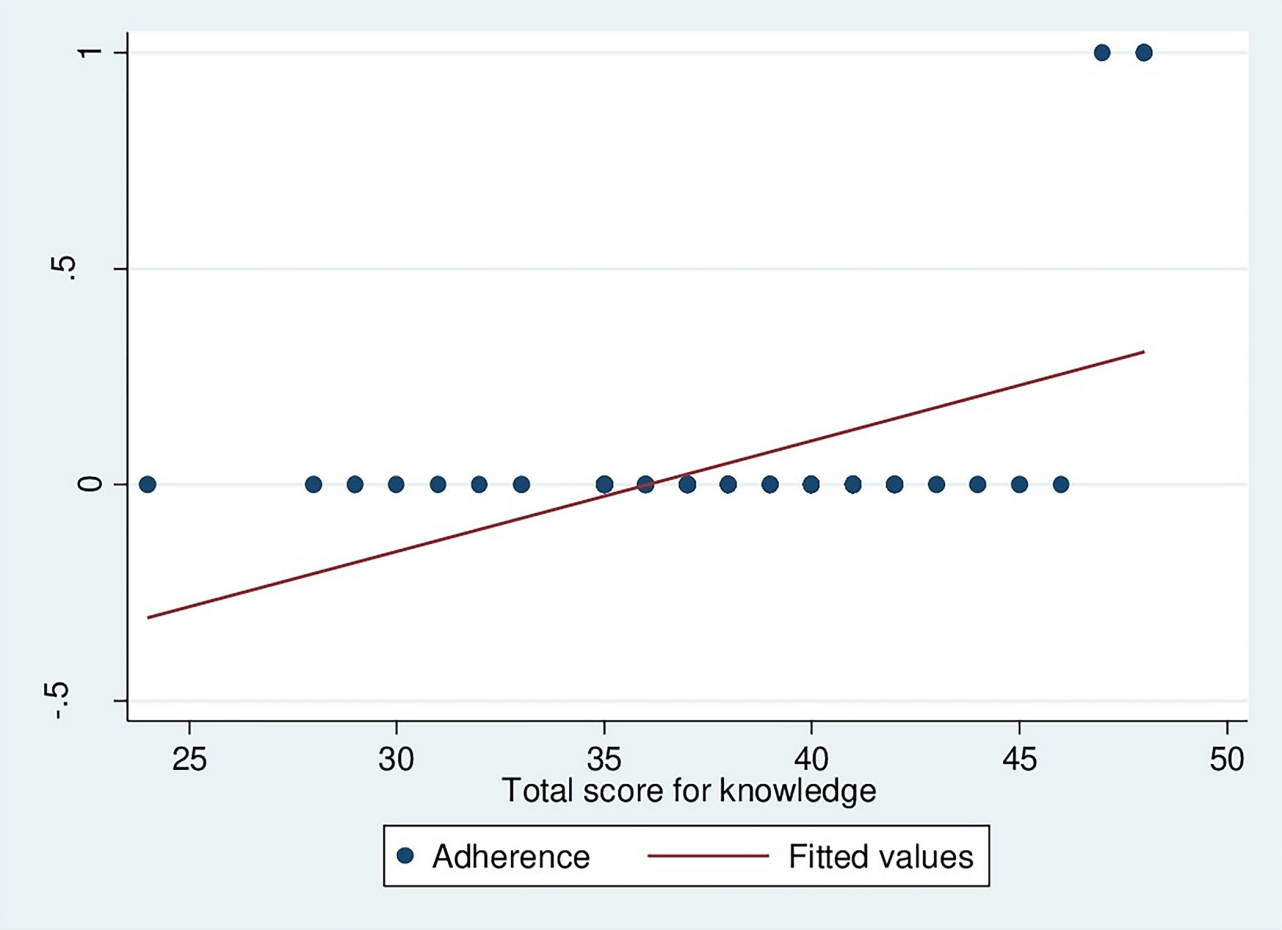
Correlation between knowledge of HCW handlers on HCW management and adherence to HCW management guideline.

**Fig 5:**
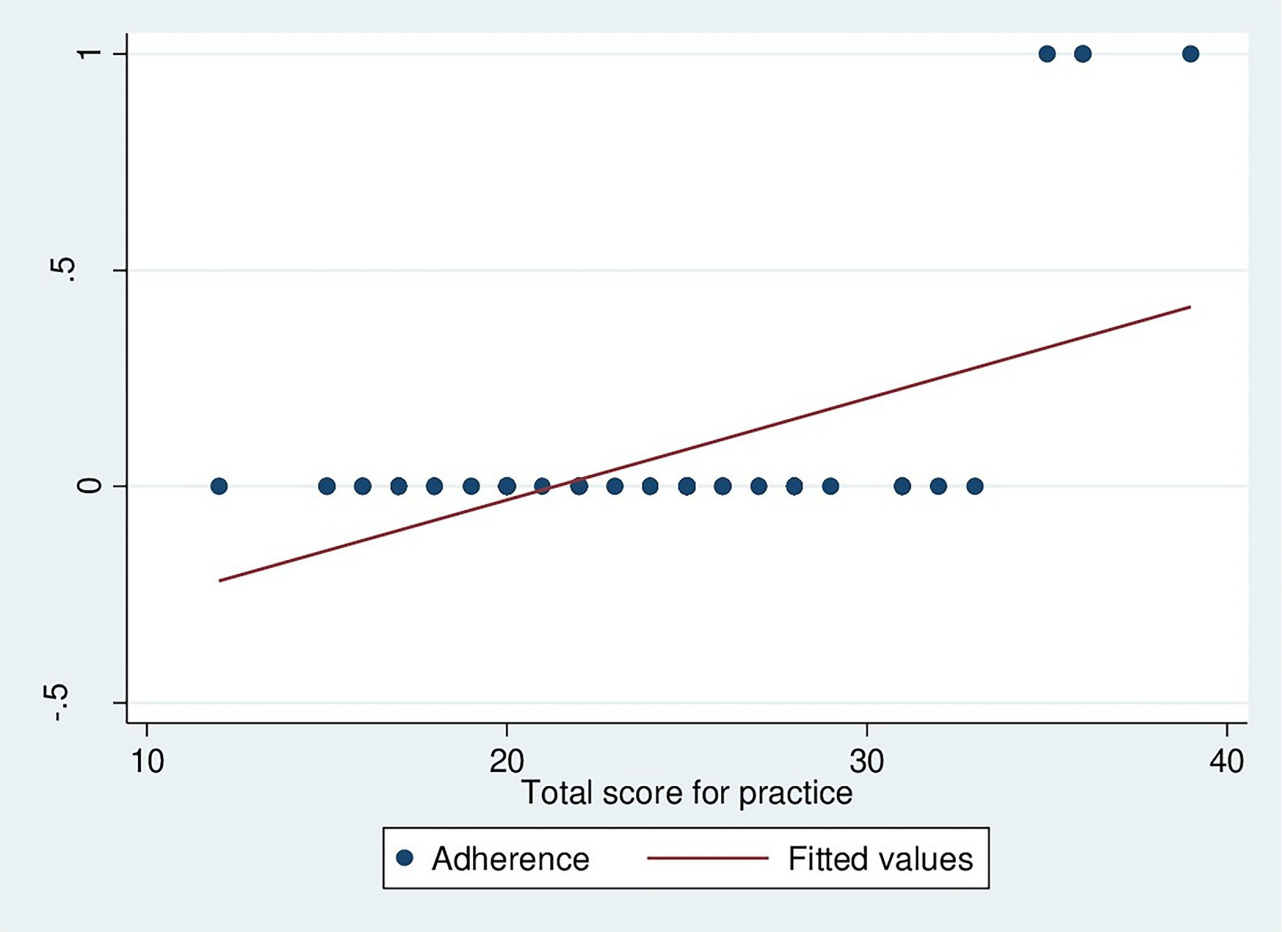
Correlation between HCW management practices among HCW handlers and adherence to HCW management guidelines.

The correlation between knowledge of HCW handlers on HCW management and adherence to HCW management guidelines was strongly correlated (r = 0.778, p<0.001), as presented in Fig 4. It means that an increase in knowledge of HCW handlers increases the adherence to HCW management guidelines. However, in this study, there was a very weak correlation between HCW management practices among HCW handlers and adherence to HCW management guidelines (r = 0.0842, p˂0.001), as depicted in Fig 5.

## Findings from the qualitative study

### Socio-demographic characteristics of qualitative participants

There were a total of 10 participants for KIIs among them one was female and the rest were male. The mean age of participants was 35.80±6.86, as presented in Table 9.

**Table 9:** Socio-demographic characteristic of participants involving in qualitative study.

Variables	Frequency	Percentage
Age (Years)	Mean age: 35.80±6.86	
20–25	0	
26–30	3	30.00
31–40	4	40.00
41–45	2	20.00
>45	1	10.00
**Gender**		
Male	9	90.00
Female	1	10.00
**Current Marital Status**		
Unmarried	3	30.00
Married	7	70.00
Widow	0	0.00
**Education**		
Secondary Level	0	0.00
Bachelor level	6	60.00
Master level	4	40.00
**Working Experience**	**Mean Experience: 7.60±4.427**	
≥ 5 Years	4	40.00
˃5 years	6	60.00

For the qualitative data analysis, Key informant interviews (KIIs) were performed (S1 File). Ten participants were selected for the interviews from Madhesh Province. The average time for an interview was 30–35 minutes.

The six phase qualitative data analysis process was used to analyze the qualitative data which includes data familiarization, generating initial codes, generating themes, reviewing potential themes, defining and renaming theme, and producing the report [41].

### Themes and codes

The themes and codes (Striking image) were generated by using six steps. RQDA was used to generate themes, as presented in Table 10. The method of qualitative data analysis is explained in the data analysis section previously.

**Table 10:** Theme and codes.

Theme	Codes
Steps HCWM	Segregation, Collection, Transport, Sterilization, Store, Disposed and periodically Collection
Challenges of Guideline	Staff transfer, Train resources, Purchasing mechanism, Hospital premises, laboratory waste,
Monitoring and evaluation	Checklist, Manpower, Recording, logbook,
Training and Awareness	Environmental Effect, Facilitation,
Occupational Health	ART service, Vaccine, Insurance, Personal Protective Equipment, Supply for waste handlers,
Planning and Organizational issues	Planning implementation, Policy, Manpower, Budget allocation,

### Steps of HCW management

The qualitative findings highlighted that the steps of HCWM are not properly followed at district level hospitals, majority of respondents perceived that segregation process of HCW was not done according to the guidelines. Patients and visitors disposed of all decomposed and non-decomposed waste in a single dustbin. In addition, the health worker also disposed of hazardous waste at normal waste collector dustbin. It was perceived that segregation, transportation, sterilization, and storage of HCW were serious issues related HCWM in district hospitals.

"Patient parties mix up a different type of HCW together like mixing needles, cotton and blood cotton." KII 1

“Patients and visitors put all kinds of waste in one bucket, and this is the first issue, and another issue is not sufficient space to store the HCW at hospital premises. So, HCW mixed at one place after collection.” KII 2

In many district hospitals, there were no central waste collection areas, and they lacked HCW treatment facilities too. There was un-availability of waste treatment plant in hospital premises.

“There is a problem in center collection side, the municipality is helping but they are helping only for non-hazardous waste.” KII 9

“All infectious HCW be disposed of only after autoclaving. However, it has not been done at a district hospital.” KII 1

### Challenges of guideline

The participants revealed many challenges to related HCWM guidelines. Among them, some claimed that many hospitals did not have adequate HCWM facilities due to lack of adequate hospital space and physical facilities, Placenta pit, as well as frequent staffs transfer, procurement problems on amenities for HCWM for HCWM, and availability of HCWM officer. There was no provision of laboratory waste management in the district level Hospital of Madhesh Province. These were the challenges of HCWM guidelines to implement in all district hospitals of Madhesh Province.

“We have not a chemical waste treatment facility in our hospital.” KII5

“Till now there is not a placenta pit at our hospital to manage placenta.” KII 2

### Monitoring and evaluation

The documentation on healthcare waste was not properly managed in district-level hospitals. Many participants claimed that there were low regular follow-ups from higher authorities which are essential for monitoring and evaluation of hospital and healthcare waste management. However, few hospitals had been properly conducted in the district hospitals for HCW management.

“We do not have any checklist and logbooks for the waste generated by the hospital.” KII 9

“Persons doing monitoring might have confused and less knowledge regarding monitoring, lack of understanding due to temporary placement in hospitals.” KII 10

### Training and awareness

Many of the respondents said that HCW handlers were not properly trained HCWM. So, HCWM had been a problem in the district-level hospitals.

"The training program is very important for cleaning staff to ensure proper HCWM. To ensure participation of every staff; training program should be conducted frequently (2–3 participants at one session)". KII 10

### Occupational health and safety

We found that many HCW handlers were unaware of HCW and most of them have not received training. However, few hospitals supplied PPE sets to waste handlers regularly which motivated and supported them to conduct HCWM.

"We are providing PPE such as masks and gloves to ensure the safety of the waste handlers in our hospital daily." KII 4

“We are providing PPEs, masks, and gloves to the waste workers regularly. But the main issue we need is proper training and awareness program.” KII 5

However, certain hospitals provided vaccination and post-exposure services to their staffs including HCW handlers.

“Hospital provide ART services to HIV infected staffs of hospital after exposure of HIV. KII 1

“We have a regular vaccination program and provide it to HCW handlers after any injury e.g. TT, Hepatitis B Vaccine.” KII 9

“Treatment should be done without any formality to HCW handlers after exposure to and injuries related to hazardous waste by the government. However, it is not applicable for HCW handlers.” KII 3

### Planning and organizational issues

The salary of the HCW handlers was extremely low. So, they were not fully motivated by their responsibility. However, few hospitals had an effective HCWM committee and they included all department chief in HCWM committee.

“Government must understand that the salary is very low that demotivates staffs in managing waste at the district hospital.” KII 6

“If so, we will include emergency in-charge, heads of all departments, indoor in-charge, medical recorder, lab in-charge, administration, and finance head in HCWM planning.” KII 4

## Discussion

In this study, the median age of HCW handlers was 35 years. A similar study also reported that the mean age of participants working for HCWM in Kathmandu was 41.94 years [35]. The mean age of the previous study is higher than the current study. It may be due to the retention of HCW handlers in the hospital. In this study, more than three-fifth of the participants were female and one-third of the participants were male. A similar study conducted in Nepal reported that 24.2% males and 75.8% of females were participated in the study [35]. This seems near to similar involvement of people in the healthcare waste handler’s profession. Among the total participants of this study, nearly one-third of participants were illiterate. Similarly, one-fourth of participants had completed non-formal education, slightly more than one-fourth of participants had completed primary level education, only few of participants had completed some secondary and PCL/above. Likewise, a study conducted in Ethiopia in 2016 among HCW handlers found that more than 45.4% of HCW handlers had completed the diploma/certificate level and the remaining had completed primary schools/below [42]. The education level of waste handlers was higher than the current study. It may result the better adherence to HCWM guidelines.

In the current study, half of participants had adequate knowledge of Healthcare waste management in District Hospitals of Madhesh Province. A similar study conducted in Kathmandu reported that 50.8% of participants had adequate knowledge on Healthcare waste management [35]. A similar study from Bangladesh reported that (56%) of HCW handlers had adequate knowledge of HCW management [43]. It may be due to the same regional practices and similar knowledge of HCW management. As well as a similar study conducted in Ethiopia reported that 45.5% of participants had adequate knowledge on HCWM, and 80% of participants had performed adequate practice for HCWM [33]. As discussed above the proper disposal of HCW may save from soil contamination, anti-biotic resistant, and water contamination [18, 19].

In the current study, it revealed that patients’ parties or visitors were the major factors who had major role to contaminate or mix all types of waste at one bin. A similar study also reported that separation of infectious medical waste is of little practical value because all types of waste has been eventually mixed together [44].

In the current study, third-fifth of HCW handlers were not trained for HCWM. In a similar study conducted in southwest Ethiopia reported that less than two-fifths (28.8%) proportion of health workers had received training of HCWM [45]. As well as in another similar study in Nigeria, it was found that only 57.3% healthcare worker had trained for HCW management [46]. The HCWM training for HCW handlers is important to safe from hospital waste hazards to HCW handlers, health staffs, patients, and patients’ visitors. Small numbers of trained staff could not work efficiently in a hospital. Most of the key stakeholders perceived that many of the HCW handlers were not trained in HCWM. So, HCWM had been a problem in the district-level hospital. A similar study also reported that cleaning staff had superficial knowledge on HCWM but not properly manage the waste in proper way [44, 47]. Proper training on HCW for waste handlers save them needle stick injury and other environmental hazards [48, 49].

In this study, about one-third of participants had used duty gloves, about one-fifth had used boots, and about two-five had never used an apron while handling HCW. A similar study conducted in a Sub-regional hospital in Birgunj in 2006 showed that all sweepers had used gloves, about 16% had used an apron and only a few (3%) had used boots [33]. Most of the healthcare waste handlers were not aware of healthcare waste risk. So, many of them had not used PPE properly. Mainly, PPE is crucial to saving healthcare waste handlers from different infectious diseases including hepatitis B, *Staphylococcus aureus*, *Pseudomonas aeruginosa*, and other skin allergy-related problems [48, 50, 51].

Gloves and boots are major safety equipment to protect from hazards during risky work. Low use of these materials increases the risk of injuries among HCW handlers.

In the current study, all participants had never recorded the collection and disposal of HCW. Another study conducted in the Terai region of Nepal also shows the same result. There was no provision for documentation and registration of collected waste [43]. The recording and documentation of waste generated in hospital and its management including regular monitoring and evaluation from higher authority influence in better healthcare waste management. However, many key stakeholders of the hospital accepted that many hospitals lack checklist and logbooks for the waste generated record and management. Furthermore, there were no regular or weak monitoring and evaluation was conducted by higher level authority in district level hospital of Madhesh province [47]. In this study, about 30% of participants reported that hospitals were not provided adequate amenities at hospital for healthcare waste management.

The documentation for HCW production and its management is essential for further management of different healthcare waste within a hospital and for making national policy on HCW management. The current study revealed that many challenges in implementation of HCWM guideline in Madhesh province include Human resources, inadequate hospital spaces and physical facilities like placenta pit, procurement-related issues on amenities for HCWM, and availability of HCWM officers. A similar study also reported that the revision and commitment of implementation of HCWM guideline is crucial in hospital for better hygiene of hospital including proper management diapers of patients [52]. A non-documented work could not support in any policy work. So, it may affect the policy-making process regarding HCW management.

In the current study, it was reported that more than half of the participants had performed inadequate HCWM practices at district hospitals of Madhesh Province. A similar study conducted in KwaZulu-Natal, south Africa reported that 29.4% of participants had performed poor practices for healthcare waste management [53]. As well as a similar study conducted in Ethiopia reported that 80% of participants had performed adequate practice for HCWM [33]. It may be due to the some supporting staffs hiring policy. In Nepal, the non-grade staffs used to be hired on contract basis locally by the hospital operating committee which would have compromised HCW handling practice due to a lack of knowledge and training among them.

About seventh-tenth of participants strongly agreed that the health facilities had supplied regular PPE sets to the participants. The study shows that many HCW handlers were not aware of HCW and most of them have not received training. However, few hospitals supply PPE sets to waste handlers regularly.

“We are providing PPEs, masks, and gloves to the waste workers regularly. But the main issue we need is proper training and awareness program.” KII 5

"We are providing PPE such as masks, and gloves daily." KII 4

A similar study from Kenya reported a better supply of PPE by healthcare institutions to HCW handlers [34]. Another similar study revealed that the demand of PPE was high for HCW handlers in healthcare institutions. Because they thought that these are essential to save from many infectious diseases [13]. Another similar study also reported that poor safety of HCW worker increases the high-risk during waste handling [54]. This study was conducted during the COVID-19 period and found that PPE was satisfactory. Which had helped to safe many HCW handlers from Hospital hazards during COVID-19 pandemic in Nepal.

In the current study 58.33% of participants were familiar with HCWM guidelines. In the current study the challenges of HCWM guideline were attitude of staffs, hospital premises, staffs transfer, procurement, unmanaged dumping of HCW and HCWM officers.

“Behavior change and the discipline of the top to bottom workers are equally important in HCWM at district hospitals.” KII 5

An organization or a working modality needs guidelines to work in an efficient manner. Without guidelines, any process could not function properly. The study was conducted at COVID-19 period, despite training on healthcare waste management, many hospital had provided PPE to HCW handlers. A similar study also reported that lack of sensitivity and resources effects on healthcare waste management in hospital [52]. A similar study reported that the role of the staff and their attitude and behavior is crucial for healthcare management in hospital. However, some people do not think that this is a common good and do not care about it [55]. In the district’s hospitals of Madhesh Province, only half of the HCW handlers were familiar with HCWM guidelines. So, it shows many HCW handlers were working without proper guidance at hospitals.

In a similar study, it was found that nurses were more aware than HCW handlers of HCWM guideline [34]. As well as in another similar study, it was found that 64% of cleaning staffs know about the policies/legislation/guidelines on HCW management [3]. A similar study suggested that the behavior and socio-economic status of HCW handlers also affect HCW management [42].

The current study shows that 85% of HCW handlers agreed that there were waste dustbins in hospital. A similar study found that less (59.69%) adequate waste dustbins were available in hospitals compared to the current study [34]. Proper placement of waste dust bins is also essential to collect waste at the proper place for a proper time. Due to the COVID-19 pandemic hospital administration supplied appropriate and enough waste dust bins in district hospitals.

In the current study, many key stakeholders accepted that there were a issues related to HCW handlers remuneration and coordination issues in participation in HCWM committee. Which is an essential point for HCWM in hospital. This is not followed in many hospitals of Madhesh Province. A similar study also revealed that inter-sectoral and intra-sectoral coordination is an important factor in the proper HCWM performance [56].

The current study found that only a few HCW handlers fully adhered to HCWM guidelines and about one-tenth were partially adhered. A similar study conducted in Kenya reported that 21.8% of health workers were fully adhered and 73.3% were partially adhered to the medical waste guidelines [34]. The adherence to HCWM among HCW handlers was more than in the current study. This may be due to the higher educational level and high retention of HCW handlers in the previous study.

## Limitations

There are certain limitations in the study. First, the study was conducted during the peak time when COVID-19 was spreading rapidly. Thus, adequate observations for all the components of health care waste management could not be made due to pandemic restrictions. So, the research was conducted using phone conversation with participants. The Census method was followed for data collection in the study. So, the inferential statistics could not be performed in the study. But the study provides an in-depth scenario of the situation and issues with the HCW management.

## Conclusion

The full adherence to HCWM guideline 2014 was extremely low among HCW handlers. The HCWs had less adequate knowledge of HCWM, and they did not practice to manage HCW adequately in district hospitals. Age had affected the knowledge and practices of HCWM among participants. However, the hospital had adequately provided amenities to manage healthcare waste. However, Knowledge and Practices of HCW handlers on HCWM had affected the adherence of healthcare waste management guideline.

Hospital administration and the government should provide training on HCWM to HCW handlers and should assess the process of HCWM practices at hospitals regularly to improve the knowledge and practices of HCW handlers which support increasing the adherence of HCWM guidelines.

## Supporting information

S1 Text HTML file developed by RQDA. (HTML)

S1 Data Quantitative data. (XLS)

S1 File Qualitative transcript file. (DOCX)
