# Supplementary material for: Knowledge about handling hazardous materials as factors associated with adherence to healthcare waste management practices among waste handlers at government district hospitals of Madhesh province, Nepal: A quantitative-qualitative methods study
Source: PLOS Glob Public Health. 2024 Dec 5;4(12):e0002028. doi: 10.1371/journal.pgph.0002028 (PMC11620432; doi:10.1371/journal.pgph.0002028)
Supplement: S1 Text — (HTML) [file pgph.0002028.s001.html]

   window.NREUM||(NREUM={});NREUM.info = {"beacon":"bam.nr-data.net","errorBeacon":"bam.nr-data.net","licenseKey":"NRJS-bbf55bdee5f59ed6475","applicationID":"1567086821","transactionName":"MwAHY0oCWxFZV0RaXgpKJGRoTHENT1pcXFAALQRZXA9QEA==","queueTime":0,"applicationTime":94,"agent":"","atts":""}  (window.NREUM||(NREUM={})).init={privacy:{cookies_enabled:true},ajax:{deny_list:[]},session_replay:{sampling_rate:1.0E-6,mask_text_selector:"*",block_selector:"",mask_input_options:{},mask_all_inputs:true,enabled:true,error_sampling_rate:1.0E-6},session_trace:{sampling_rate:0.0,mode:"FIXED_RATE",enabled:true,error_sampling_rate:0.0},distributed_tracing:{enabled:true}};(window.NREUM||(NREUM={})).loader_config={agentID:"1588829430",accountID:"3382936",trustKey:"2038175",xpid:"VwUPU19QDhABUVdVBwkBXlYA",licenseKey:"NRJS-bbf55bdee5f59ed6475",applicationID:"1567086821"};;/*! For license information please see nr-loader-spa-1.272.0.min.js.LICENSE.txt */
(()=>{var e,t,r={8122:(e,t,r)=>{"use strict";r.d(t,{a:()=>i});var n=r(944);function i(e,t){try{if(!e||"object"!=typeof e)return(0,n.R)(3);if(!t||"object"!=typeof t)return(0,n.R)(4);const r=Object.create(Object.getPrototypeOf(t),Object.getOwnPropertyDescriptors(t)),o=0===Object.keys(r).length?e:r;for(let a in o)if(void 0!==e[a])try{if(null===e[a]){r[a]=null;continue}Array.isArray(e[a])&&Array.isArray(t[a])?r[a]=Array.from(new Set([...e[a],...t[a]])):"object"==typeof e[a]&&"object"==typeof t[a]?r[a]=i(e[a],t[a]):r[a]=e[a]}catch(e){(0,n.R)(1,e)}return r}catch(e){(0,n.R)(2,e)}}},2555:(e,t,r)=>{"use strict";r.d(t,{Vp:()=>c,fn:()=>s,x1:()=>u});var n=r(384),i=r(8122);const o={beacon:n.NT.beacon,errorBeacon:n.NT.errorBeacon,licenseKey:void 0,applicationID:void 0,sa:void 0,queueTime:void 0,applicationTime:void 0,ttGuid:void 0,user:void 0,account:void 0,product:void 0,extra:void 0,jsAttributes:{},userAttributes:void 0,atts:void 0,transactionName:void 0,tNamePlain:void 0},a={};function s(e){try{const t=c(e);return!!t.licenseKey&&!!t.errorBeacon&&!!t.applicationID}catch(e){return!1}}function c(e){if(!e)throw new Error("All info objects require an agent identifier!");if(!a[e])throw new Error("Info for ".concat(e," was never set"));return a[e]}function u(e,t){if(!e)throw new Error("All info objects require an agent identifier!");a[e]=(0,i.a)(t,o);const r=(0,n.nY)(e);r&&(r.info=a[e])}},9417:(e,t,r)=>{"use strict";r.d(t,{D0:()=>h,gD:()=>g,xN:()=>p});var n=r(993);const i=e=>{if(!e||"string"!=typeof e)return!1;try{document.createDocumentFragment().querySelector(e)}catch{return!1}return!0};var o=r(2614),a=r(944),s=r(384),c=r(8122);const u="[data-nr-mask]",d=()=>{const e={mask_selector:"*",block_selector:"[data-nr-block]",mask_input_options:{color:!1,date:!1,"datetime-local":!1,email:!1,month:!1,number:!1,range:!1,search:!1,tel:!1,text:!1,time:!1,url:!1,week:!1,textarea:!1,select:!1,password:!0}};return{ajax:{deny_list:void 0,block_internal:!0,enabled:!0,harvestTimeSeconds:10,autoStart:!0},distributed_tracing:{enabled:void 0,exclude_newrelic_header:void 0,cors_use_newrelic_header:void 0,cors_use_tracecontext_headers:void 0,allowed_origins:void 0},feature_flags:[],generic_events:{enabled:!0,harvestTimeSeconds:30,autoStart:!0},harvest:{tooManyRequestsDelay:60},jserrors:{enabled:!0,harvestTimeSeconds:10,autoStart:!0},logging:{enabled:!0,harvestTimeSeconds:10,autoStart:!0,level:n.p_.INFO},metrics:{enabled:!0,autoStart:!0},obfuscate:void 0,page_action:{enabled:!0},page_view_event:{enabled:!0,autoStart:!0},page_view_timing:{enabled:!0,harvestTimeSeconds:30,autoStart:!0},performance:{capture_marks:!1,capture_measures:!1},privacy:{cookies_enabled:!0},proxy:{assets:void 0,beacon:void 0},session:{expiresMs:o.wk,inactiveMs:o.BB},session_replay:{autoStart:!0,enabled:!1,harvestTimeSeconds:60,preload:!1,sampling_rate:10,error_sampling_rate:100,collect_fonts:!1,inline_images:!1,fix_stylesheets:!0,mask_all_inputs:!0,get mask_text_selector(){return e.mask_selector},set mask_text_selector(t){i(t)?e.mask_selector="".concat(t,",").concat(u):""===t||null===t?e.mask_selector=u:(0,a.R)(5,t)},get block_class(){return"nr-block"},get ignore_class(){return"nr-ignore"},get mask_text_class(){return"nr-mask"},get block_selector(){return e.block_selector},set block_selector(t){i(t)?e.block_selector+=",".concat(t):""!==t&&(0,a.R)(6,t)},get mask_input_options(){return e.mask_input_options},set mask_input_options(t){t&&"object"==typeof t?e.mask_input_options={...t,password:!0}:(0,a.R)(7,t)}},session_trace:{enabled:!0,harvestTimeSeconds:10,autoStart:!0},soft_navigations:{enabled:!0,harvestTimeSeconds:10,autoStart:!0},spa:{enabled:!0,harvestTimeSeconds:10,autoStart:!0},ssl:void 0,user_actions:{enabled:!0}}},l={},f="All configuration objects require an agent identifier!";function h(e){if(!e)throw new Error(f);if(!l[e])throw new Error("Configuration for ".concat(e," was never set"));return l[e]}function p(e,t){if(!e)throw new Error(f);l[e]=(0,c.a)(t,d());const r=(0,s.nY)(e);r&&(r.init=l[e])}function g(e,t){if(!e)throw new Error(f);var r=h(e);if(r){for(var n=t.split("."),i=0;i {"use strict";r.d(t,{a:()=>c,o:()=>s});var n=r(384),i=r(8122);const o={accountID:void 0,trustKey:void 0,agentID:void 0,licenseKey:void 0,applicationID:void 0,xpid:void 0},a={};function s(e){if(!e)throw new Error("All loader-config objects require an agent identifier!");if(!a[e])throw new Error("LoaderConfig for ".concat(e," was never set"));return a[e]}function c(e,t){if(!e)throw new Error("All loader-config objects require an agent identifier!");a[e]=(0,i.a)(t,o);const r=(0,n.nY)(e);r&&(r.loader_config=a[e])}},3371:(e,t,r)=>{"use strict";r.d(t,{V:()=>f,f:()=>l});var n=r(8122),i=r(384),o=r(6154),a=r(9324);let s=0;const c={buildEnv:a.F3,distMethod:a.Xs,version:a.xv,originTime:o.WN},u={customTransaction:void 0,disabled:!1,isolatedBacklog:!1,loaderType:void 0,maxBytes:3e4,onerror:void 0,ptid:void 0,releaseIds:{},appMetadata:{},session:void 0,denyList:void 0,timeKeeper:void 0,obfuscator:void 0},d={};function l(e){if(!e)throw new Error("All runtime objects require an agent identifier!");if(!d[e])throw new Error("Runtime for ".concat(e," was never set"));return d[e]}function f(e,t){if(!e)throw new Error("All runtime objects require an agent identifier!");d[e]={...(0,n.a)(t,u),...c},Object.hasOwnProperty.call(d[e],"harvestCount")||Object.defineProperty(d[e],"harvestCount",{get:()=>++s});const r=(0,i.nY)(e);r&&(r.runtime=d[e])}},9324:(e,t,r)=>{"use strict";r.d(t,{F3:()=>i,Xs:()=>o,Yq:()=>a,xv:()=>n});const n="1.272.0",i="PROD",o="CDN",a="2.0.0-alpha.12"},6154:(e,t,r)=>{"use strict";r.d(t,{A4:()=>s,OF:()=>d,RI:()=>i,WN:()=>h,bv:()=>o,gm:()=>a,lR:()=>f,m:()=>u,mw:()=>c,sb:()=>l});var n=r(1863);const i="undefined"!=typeof window&&!!window.document,o="undefined"!=typeof WorkerGlobalScope&&("undefined"!=typeof self&&self instanceof WorkerGlobalScope&&self.navigator instanceof WorkerNavigator||"undefined"!=typeof globalThis&&globalThis instanceof WorkerGlobalScope&&globalThis.navigator instanceof WorkerNavigator),a=i?window:"undefined"!=typeof WorkerGlobalScope&&("undefined"!=typeof self&&self instanceof WorkerGlobalScope&&self||"undefined"!=typeof globalThis&&globalThis instanceof WorkerGlobalScope&&globalThis),s="complete"===a?.document?.readyState,c=Boolean("hidden"===a?.document?.visibilityState),u=""+a?.location,d=/iPad|iPhone|iPod/.test(a.navigator?.userAgent),l=d&&"undefined"==typeof SharedWorker,f=(()=>{const e=a.navigator?.userAgent?.match(/Firefox[/\s](\d+\.\d+)/);return Array.isArray(e)&&e.length>=2?+e[1]:0})(),h=Date.now()-(0,n.t)()},7295:(e,t,r)=>{"use strict";r.d(t,{Xv:()=>a,gX:()=>i,iW:()=>o});var n=[];function i(e){if(!e||o(e))return!1;if(0===n.length)return!0;for(var t=0;t 0?(o=r.substring(0,i),a=r.substring(i)):(o=r,a="");let[s]=o.split(":");n.push({hostname:s,pathname:a})}}function s(e,t){return!(e.length>t.length)&&t.indexOf(e)===t.length-e.length}function c(e,t){return 0===e.indexOf("/")&&(e=e.substring(1)),0===t.indexOf("/")&&(t=t.substring(1)),""===e||e===t}},1687:(e,t,r)=>{"use strict";r.d(t,{Ak:()=>c,Ze:()=>l,x3:()=>u});var n=r(7836),i=r(3606),o=r(860),a=r(2646);const s={};function c(e,t){const r={staged:!1,priority:o.P[t]||0};d(e),s[e].get(t)||s[e].set(t,r)}function u(e,t){e&&s[e]&&(s[e].get(t)&&s[e].delete(t),h(e,t,!1),s[e].size&&f(e))}function d(e){if(!e)throw new Error("agentIdentifier required");s[e]||(s[e]=new Map)}function l(e="",t="feature",r=!1){if(d(e),!e||!s[e].get(t)||r)return h(e,t);s[e].get(t).staged=!0,f(e)}function f(e){const t=Array.from(s[e]);t.every((([e,t])=>t.staged))&&(t.sort(((e,t)=>e[1].priority-t[1].priority)),t.forEach((([t])=>{s[e].delete(t),h(e,t)})))}function h(e,t,r=!0){const o=e?n.ee.get(e):n.ee,s=i.i.handlers;if(!o.aborted&&o.backlog&&s){if(r){const e=o.backlog[t],r=s[t];if(r){for(let t=0;e&&t {Object.values(t||{}).forEach((t=>{t[0]?.on&&t[0]?.context()instanceof a.y&&t[0].on(e,t[1])}))}))}}o.isolatedBacklog||delete s[t],o.backlog[t]=null,o.emit("drain-"+t,[])}}function p(e,t){var r=e[1];Object.values(t[r]||{}).forEach((t=>{var r=e[0];if(t[0]===r){var n=t[1],i=e[3],o=e[2];n.apply(i,o)}}))}},7836:(e,t,r)=>{"use strict";r.d(t,{P:()=>c,ee:()=>u});var n=r(384),i=r(8990),o=r(3371),a=r(2646),s=r(5607);const c="nr@context:".concat(s.W),u=function e(t,r){var n={},s={},d={},l=!1;try{l=16===r.length&&(0,o.f)(r).isolatedBacklog}catch(e){}var f={on:p,addEventListener:p,removeEventListener:function(e,t){var r=n[e];if(!r)return;for(var i=0;i {s[n]=t,t in r||(r[t]=[])}))},abort:function(){f._aborted=!0,Object.keys(f.backlog).forEach((e=>{delete f.backlog[e]}))},isBuffering:function(e){return!!v()[s[e]]},debugId:r,backlog:l?{}:t&&"object"==typeof t.backlog?t.backlog:{},isolatedBacklog:l};return Object.defineProperty(f,"aborted",{get:()=>{let e=f._aborted||!1;return e||(t&&(e=t.aborted),e)}}),f;function h(e){return e&&e instanceof a.y?e:e?(0,i.I)(e,c,(()=>new a.y(c))):new a.y(c)}function p(e,t){n[e]=g(e).concat(t)}function g(e){return n[e]||[]}function m(t){return d[t]=d[t]||e(f,t)}function v(){return f.backlog}}(void 0,"globalEE"),d=(0,n.Zm)();d.ee||(d.ee=u)},2646:(e,t,r)=>{"use strict";r.d(t,{y:()=>n});class n{constructor(e){this.contextId=e}}},9908:(e,t,r)=>{"use strict";r.d(t,{d:()=>n,p:()=>i});var n=r(7836).ee.get("handle");function i(e,t,r,i,o){o?(o.buffer([e],i),o.emit(e,t,r)):(n.buffer([e],i),n.emit(e,t,r))}},3606:(e,t,r)=>{"use strict";r.d(t,{i:()=>o});var n=r(9908);o.on=a;var i=o.handlers={};function o(e,t,r,o){a(o||n.d,i,e,t,r)}function a(e,t,r,i,o){o||(o="feature"),e||(e=n.d);var a=t[o]=t[o]||{};(a[r]=a[r]||[]).push([e,i])}},3878:(e,t,r)=>{"use strict";function n(e,t){return{capture:e,passive:!1,signal:t}}function i(e,t,r=!1,i){window.addEventListener(e,t,n(r,i))}function o(e,t,r=!1,i){document.addEventListener(e,t,n(r,i))}r.d(t,{DD:()=>o,jT:()=>n,sp:()=>i})},5607:(e,t,r)=>{"use strict";r.d(t,{W:()=>n});const n=(0,r(9566).bz)()},9566:(e,t,r)=>{"use strict";r.d(t,{LA:()=>s,ZF:()=>c,bz:()=>a,el:()=>u});var n=r(6154);const i="xxxxxxxx-xxxx-4xxx-yxxx-xxxxxxxxxxxx";function o(e,t){return e?15&e[t]:16*Math.random()|0}function a(){const e=n.gm?.crypto||n.gm?.msCrypto;let t,r=0;return e&&e.getRandomValues&&(t=e.getRandomValues(new Uint8Array(30))),i.split("").map((e=>"x"===e?o(t,r++).toString(16):"y"===e?(3&o()|8).toString(16):e)).join("")}function s(e){const t=n.gm?.crypto||n.gm?.msCrypto;let r,i=0;t&&t.getRandomValues&&(r=t.getRandomValues(new Uint8Array(e)));const a=[];for(var s=0;s {"use strict";r.d(t,{BB:()=>a,H3:()=>n,g:()=>u,iL:()=>c,tS:()=>s,uh:()=>i,wk:()=>o});const n="NRBA",i="SESSION",o=144e5,a=18e5,s={STARTED:"session-started",PAUSE:"session-pause",RESET:"session-reset",RESUME:"session-resume",UPDATE:"session-update"},c={SAME_TAB:"same-tab",CROSS_TAB:"cross-tab"},u={OFF:0,FULL:1,ERROR:2}},1863:(e,t,r)=>{"use strict";function n(){return Math.floor(performance.now())}r.d(t,{t:()=>n})},7485:(e,t,r)=>{"use strict";r.d(t,{D:()=>i});var n=r(6154);function i(e){if(0===(e||"").indexOf("data:"))return{protocol:"data"};try{const t=new URL(e,location.href),r={port:t.port,hostname:t.hostname,pathname:t.pathname,search:t.search,protocol:t.protocol.slice(0,t.protocol.indexOf(":")),sameOrigin:t.protocol===n.gm?.location?.protocol&&t.host===n.gm?.location?.host};return r.port&&""!==r.port||("http:"===t.protocol&&(r.port="80"),"https:"===t.protocol&&(r.port="443")),r.pathname&&""!==r.pathname?r.pathname.startsWith("/")||(r.pathname="/".concat(r.pathname)):r.pathname="/",r}catch(e){return{}}}},944:(e,t,r)=>{"use strict";function n(e,t){"function"==typeof console.debug&&console.debug("New Relic Warning: https://github.com/newrelic/newrelic-browser-agent/blob/main/docs/warning-codes.md#".concat(e),t)}r.d(t,{R:()=>n})},5284:(e,t,r)=>{"use strict";r.d(t,{t:()=>c,B:()=>s});var n=r(7836),i=r(6154);const o="newrelic";const a=new Set,s={};function c(e,t){const r=n.ee.get(t);s[t]??={},e&&"object"==typeof e&&(a.has(t)||(r.emit("rumresp",[e]),s[t]=e,a.add(t),function(e={}){try{i.gm.dispatchEvent(new CustomEvent(o,{detail:e}))}catch(e){}}({loaded:!0})))}},8990:(e,t,r)=>{"use strict";r.d(t,{I:()=>i});var n=Object.prototype.hasOwnProperty;function i(e,t,r){if(n.call(e,t))return e[t];var i=r();if(Object.defineProperty&&Object.keys)try{return Object.defineProperty(e,t,{value:i,writable:!0,enumerable:!1}),i}catch(e){}return e[t]=i,i}},6389:(e,t,r)=>{"use strict";function n(e,t=500,r={}){const n=r?.leading||!1;let i;return(...r)=>{n&&void 0===i&&(e.apply(this,r),i=setTimeout((()=>{i=clearTimeout(i)}),t)),n||(clearTimeout(i),i=setTimeout((()=>{e.apply(this,r)}),t))}}function i(e){let t=!1;return(...r)=>{t||(t=!0,e.apply(this,r))}}r.d(t,{J:()=>i,s:()=>n})},3304:(e,t,r)=>{"use strict";r.d(t,{A:()=>o});var n=r(7836);const i=()=>{const e=new WeakSet;return(t,r)=>{if("object"==typeof r&&null!==r){if(e.has(r))return;e.add(r)}return r}};function o(e){try{return JSON.stringify(e,i())??""}catch(e){try{n.ee.emit("internal-error",[e])}catch(e){}return""}}},5289:(e,t,r)=>{"use strict";r.d(t,{GG:()=>o,sB:()=>a});var n=r(3878);function i(){return"undefined"==typeof document||"complete"===document.readyState}function o(e,t){if(i())return e();(0,n.sp)("load",e,t)}function a(e){if(i())return e();(0,n.DD)("DOMContentLoaded",e)}},384:(e,t,r)=>{"use strict";r.d(t,{NT:()=>o,US:()=>d,Zm:()=>a,bQ:()=>c,dV:()=>s,nY:()=>u,pV:()=>l});var n=r(6154),i=r(1863);const o={beacon:"bam.nr-data.net",errorBeacon:"bam.nr-data.net"};function a(){return n.gm.NREUM||(n.gm.NREUM={}),void 0===n.gm.newrelic&&(n.gm.newrelic=n.gm.NREUM),n.gm.NREUM}function s(){let e=a();return e.o||(e.o={ST:n.gm.setTimeout,SI:n.gm.setImmediate,CT:n.gm.clearTimeout,XHR:n.gm.XMLHttpRequest,REQ:n.gm.Request,EV:n.gm.Event,PR:n.gm.Promise,MO:n.gm.MutationObserver,FETCH:n.gm.fetch,WS:n.gm.WebSocket}),e}function c(e,t){let r=a();r.initializedAgents??={},t.initializedAt={ms:(0,i.t)(),date:new Date},r.initializedAgents[e]=t}function u(e){let t=a();return t.initializedAgents?.[e]}function d(e,t){a()[e]=t}function l(){return function(){let e=a();const t=e.info||{};e.info={beacon:o.beacon,errorBeacon:o.errorBeacon,...t}}(),function(){let e=a();const t=e.init||{};e.init={...t}}(),s(),function(){let e=a();const t=e.loader_config||{};e.loader_config={...t}}(),a()}},2843:(e,t,r)=>{"use strict";r.d(t,{u:()=>i});var n=r(3878);function i(e,t=!1,r,i){(0,n.DD)("visibilitychange",(function(){if(t)return void("hidden"===document.visibilityState&&e());e(document.visibilityState)}),r,i)}},8139:(e,t,r)=>{"use strict";r.d(t,{u:()=>f});var n=r(7836),i=r(3434),o=r(8990),a=r(6154);const s={},c=a.gm.XMLHttpRequest,u="addEventListener",d="removeEventListener",l="nr@wrapped:".concat(n.P);function f(e){var t=function(e){return(e||n.ee).get("events")}(e);if(s[t.debugId]++)return t;s[t.debugId]=1;var r=(0,i.YM)(t,!0);function f(e){r.inPlace(e,[u,d],"-",p)}function p(e,t){return e[1]}return"getPrototypeOf"in Object&&(a.RI&&h(document,f),h(a.gm,f),h(c.prototype,f)),t.on(u+"-start",(function(e,t){var n=e[1];if(null!==n&&("function"==typeof n||"object"==typeof n)){var i=(0,o.I)(n,l,(function(){var e={object:function(){if("function"!=typeof n.handleEvent)return;return n.handleEvent.apply(n,arguments)},function:n}[typeof n];return e?r(e,"fn-",null,e.name||"anonymous"):n}));this.wrapped=e[1]=i}})),t.on(d+"-start",(function(e){e[1]=this.wrapped||e[1]})),t}function h(e,t,...r){let n=e;for(;"object"==typeof n&&!Object.prototype.hasOwnProperty.call(n,u);)n=Object.getPrototypeOf(n);n&&t(n,...r)}},3434:(e,t,r)=>{"use strict";r.d(t,{Jt:()=>o,YM:()=>c});var n=r(7836),i=r(5607);const o="nr@original:".concat(i.W);var a=Object.prototype.hasOwnProperty,s=!1;function c(e,t){return e||(e=n.ee),r.inPlace=function(e,t,n,i,o){n||(n="");const a="-"===n.charAt(0);for(let s=0;s {"use strict";r.d(t,{T:()=>n});const n=r(860).K.ajax},3333:(e,t,r)=>{"use strict";r.d(t,{TZ:()=>n,Zp:()=>i,mq:()=>s,nf:()=>a,qN:()=>o});const n=r(860).K.genericEvents,i=["auxclick","click","copy","keydown","paste","scrollend"],o=["focus","blur"],a=4,s=1e3},6774:(e,t,r)=>{"use strict";r.d(t,{T:()=>n});const n=r(860).K.jserrors},993:(e,t,r)=>{"use strict";r.d(t,{ET:()=>o,TZ:()=>a,p_:()=>i});var n=r(860);const i={ERROR:"ERROR",WARN:"WARN",INFO:"INFO",DEBUG:"DEBUG",TRACE:"TRACE"},o="log",a=n.K.logging},3785:(e,t,r)=>{"use strict";r.d(t,{R:()=>c,b:()=>u});var n=r(9908),i=r(1863),o=r(860),a=r(3969),s=r(993);function c(e,t,r={},c=s.p_.INFO){(0,n.p)(a.xV,["API/logging/".concat(c.toLowerCase(),"/called")],void 0,o.K.metrics,e),(0,n.p)(s.ET,[(0,i.t)(),t,r,c],void 0,o.K.logging,e)}function u(e){return"string"==typeof e&&Object.values(s.p_).some((t=>t===e.toUpperCase().trim()))}},3969:(e,t,r)=>{"use strict";r.d(t,{TZ:()=>n,XG:()=>s,rs:()=>i,xV:()=>a,z_:()=>o});const n=r(860).K.metrics,i="sm",o="cm",a="storeSupportabilityMetrics",s="storeEventMetrics"},6630:(e,t,r)=>{"use strict";r.d(t,{T:()=>n});const n=r(860).K.pageViewEvent},782:(e,t,r)=>{"use strict";r.d(t,{T:()=>n});const n=r(860).K.pageViewTiming},6344:(e,t,r)=>{"use strict";r.d(t,{BB:()=>d,G4:()=>o,Qb:()=>l,TZ:()=>i,Ug:()=>a,_s:()=>s,bc:()=>u,yP:()=>c});var n=r(2614);const i=r(860).K.sessionReplay,o={RECORD:"recordReplay",PAUSE:"pauseReplay",REPLAY_RUNNING:"replayRunning",ERROR_DURING_REPLAY:"errorDuringReplay"},a=.12,s={DomContentLoaded:0,Load:1,FullSnapshot:2,IncrementalSnapshot:3,Meta:4,Custom:5},c={[n.g.ERROR]:15e3,[n.g.FULL]:3e5,[n.g.OFF]:0},u={RESET:{message:"Session was reset",sm:"Reset"},IMPORT:{message:"Recorder failed to import",sm:"Import"},TOO_MANY:{message:"429: Too Many Requests",sm:"Too-Many"},TOO_BIG:{message:"Payload was too large",sm:"Too-Big"},CROSS_TAB:{message:"Session Entity was set to OFF on another tab",sm:"Cross-Tab"},ENTITLEMENTS:{message:"Session Replay is not allowed and will not be started",sm:"Entitlement"}},d=5e3,l={API:"api"}},5270:(e,t,r)=>{"use strict";r.d(t,{Aw:()=>c,CT:()=>u,SR:()=>s});var n=r(384),i=r(9417),o=r(7767),a=r(6154);function s(e){return!!(0,n.dV)().o.MO&&(0,o.V)(e)&&!0===(0,i.gD)(e,"session_trace.enabled")}function c(e){return!0===(0,i.gD)(e,"session_replay.preload")&&s(e)}function u(e,t){const r=t.correctAbsoluteTimestamp(e);return{originalTimestamp:e,correctedTimestamp:r,timestampDiff:e-r,originTime:a.WN,correctedOriginTime:t.correctedOriginTime,originTimeDiff:Math.floor(a.WN-t.correctedOriginTime)}}},3738:(e,t,r)=>{"use strict";r.d(t,{He:()=>i,Kp:()=>s,Lc:()=>u,Rz:()=>d,TZ:()=>n,bD:()=>o,d3:()=>a,jx:()=>l,uP:()=>c});const n=r(860).K.sessionTrace,i="bstResource",o="resource",a="-start",s="-end",c="fn"+a,u="fn"+s,d="pushState",l=1e3},3962:(e,t,r)=>{"use strict";r.d(t,{AM:()=>o,O2:()=>s,Qu:()=>c,TZ:()=>a,ih:()=>u,tC:()=>i});var n=r(860);const i=["click","keydown","submit"],o="api",a=n.K.softNav,s={INITIAL_PAGE_LOAD:"",ROUTE_CHANGE:1,UNSPECIFIED:2},c={INTERACTION:1,AJAX:2,CUSTOM_END:3,CUSTOM_TRACER:4},u={IP:"in progress",FIN:"finished",CAN:"cancelled"}},7378:(e,t,r)=>{"use strict";r.d(t,{$p:()=>x,BR:()=>b,Kp:()=>R,L3:()=>y,Lc:()=>c,NC:()=>o,SG:()=>d,TZ:()=>i,U6:()=>p,UT:()=>m,d3:()=>w,dT:()=>f,e5:()=>A,gx:()=>v,l9:()=>l,oW:()=>h,op:()=>g,rw:()=>u,tH:()=>E,uP:()=>s,wW:()=>T,xq:()=>a});var n=r(384);const i=r(860).K.spa,o=["click","submit","keypress","keydown","keyup","change"],a=999,s="fn-start",c="fn-end",u="cb-start",d="api-ixn-",l="remaining",f="interaction",h="spaNode",p="jsonpNode",g="fetch-start",m="fetch-done",v="fetch-body-",b="jsonp-end",y=(0,n.dV)().o.ST,w="-start",R="-end",x="-body",T="cb"+R,A="jsTime",E="fetch"},4234:(e,t,r)=>{"use strict";r.d(t,{W:()=>o});var n=r(7836),i=r(1687);class o{constructor(e,t){this.agentIdentifier=e,this.ee=n.ee.get(e),this.featureName=t,this.blocked=!1}deregisterDrain(){(0,i.x3)(this.agentIdentifier,this.featureName)}}},7767:(e,t,r)=>{"use strict";r.d(t,{V:()=>o});var n=r(9417),i=r(6154);const o=e=>i.RI&&!0===(0,n.gD)(e,"privacy.cookies_enabled")},425:(e,t,r)=>{"use strict";r.d(t,{j:()=>j});var n=r(860),i=r(2555),o=r(3371),a=r(9908),s=r(7836),c=r(1687),u=r(5289),d=r(6154),l=r(944),f=r(3969),h=r(384),p=r(6344);const g=["setErrorHandler","finished","addToTrace","addRelease","addPageAction","setCurrentRouteName","setPageViewName","setCustomAttribute","interaction","noticeError","setUserId","setApplicationVersion","start",p.G4.RECORD,p.G4.PAUSE,"log","wrapLogger"],m=["setErrorHandler","finished","addToTrace","addRelease"];var v=r(1863),b=r(2614),y=r(993),w=r(3785),R=r(2646),x=r(3434);function T(e,t,r,n){if("object"!=typeof t||!t||"string"!=typeof r||!r||"function"!=typeof t[r])return(0,l.R)(29);const i=function(e){return(e||s.ee).get("logger")}(e),o=(0,x.YM)(i),a=new R.y(s.P);return a.level=n.level,a.customAttributes=n.customAttributes,o.inPlace(t,[r],"wrap-logger-",a),i}function A(){const e=(0,h.pV)();g.forEach((t=>{e[t]=(...r)=>function(t,...r){let n=[];return Object.values(e.initializedAgents).forEach((e=>{e&&e.api?e.exposed&&e.api[t]&&n.push(e.api[t](...r)):(0,l.R)(38,t)})),n.length>1?n:n[0]}(t,...r)}))}const E={};var S=r(9417),N=r(5603),O=r(5284);const _=e=>{const t=e.startsWith("http");e+="/",r.p=t?e:"https://"+e};let I=!1;function j(e,t={},g,R){let{init:x,info:j,loader_config:P,runtime:C={},exposed:k=!0}=t;C.loaderType=g;const L=(0,h.pV)();j||(x=L.init,j=L.info,P=L.loader_config),(0,S.xN)(e.agentIdentifier,x||{}),(0,N.a)(e.agentIdentifier,P||{}),j.jsAttributes??={},d.bv&&(j.jsAttributes.isWorker=!0),(0,i.x1)(e.agentIdentifier,j);const H=(0,S.D0)(e.agentIdentifier),D=[j.beacon,j.errorBeacon];I||(H.proxy.assets&&(_(H.proxy.assets),D.push(H.proxy.assets)),H.proxy.beacon&&D.push(H.proxy.beacon),A(),(0,h.US)("activatedFeatures",O.B),e.runSoftNavOverSpa&&=!0===H.soft_navigations.enabled&&H.feature_flags.includes("soft_nav")),C.denyList=[...H.ajax.deny_list||[],...H.ajax.block_internal?D:[]],C.ptid=e.agentIdentifier,(0,o.V)(e.agentIdentifier,C),e.ee=s.ee.get(e.agentIdentifier),void 0===e.api&&(e.api=function(e,t,h=!1){t||(0,c.Ak)(e,"api");const g={};var R=s.ee.get(e),x=R.get("tracer");E[e]=b.g.OFF,R.on(p.G4.REPLAY_RUNNING,(t=>{E[e]=t}));var A="api-",S=A+"ixn-";function N(t,r,n,o){const a=(0,i.Vp)(e);return null===r?delete a.jsAttributes[t]:(0,i.x1)(e,{...a,jsAttributes:{...a.jsAttributes,[t]:r}}),I(A,n,!0,o||null===r?"session":void 0)(t,r)}function O(){}g.log=function(e,{customAttributes:t={},level:r=y.p_.INFO}={}){(0,a.p)(f.xV,["API/log/called"],void 0,n.K.metrics,R),(0,w.R)(R,e,t,r)},g.wrapLogger=(e,t,{customAttributes:r={},level:i=y.p_.INFO}={})=>{(0,a.p)(f.xV,["API/wrapLogger/called"],void 0,n.K.metrics,R),T(R,e,t,{customAttributes:r,level:i})},m.forEach((e=>{g[e]=I(A,e,!0,"api")})),g.addPageAction=I(A,"addPageAction",!0,n.K.genericEvents),g.setPageViewName=function(t,r){if("string"==typeof t)return"/"!==t.charAt(0)&&(t="/"+t),(0,o.f)(e).customTransaction=(r||"http://custom.transaction")+t,I(A,"setPageViewName",!0)()},g.setCustomAttribute=function(e,t,r=!1){if("string"==typeof e){if(["string","number","boolean"].includes(typeof t)||null===t)return N(e,t,"setCustomAttribute",r);(0,l.R)(40,typeof t)}else(0,l.R)(39,typeof e)},g.setUserId=function(e){if("string"==typeof e||null===e)return N("enduser.id",e,"setUserId",!0);(0,l.R)(41,typeof e)},g.setApplicationVersion=function(e){if("string"==typeof e||null===e)return N("application.version",e,"setApplicationVersion",!1);(0,l.R)(42,typeof e)},g.start=()=>{try{(0,a.p)(f.xV,["API/start/called"],void 0,n.K.metrics,R),R.emit("manual-start-all")}catch(e){(0,l.R)(23,e)}},g[p.G4.RECORD]=function(){(0,a.p)(f.xV,["API/recordReplay/called"],void 0,n.K.metrics,R),(0,a.p)(p.G4.RECORD,[],void 0,n.K.sessionReplay,R)},g[p.G4.PAUSE]=function(){(0,a.p)(f.xV,["API/pauseReplay/called"],void 0,n.K.metrics,R),(0,a.p)(p.G4.PAUSE,[],void 0,n.K.sessionReplay,R)},g.interaction=function(e){return(new O).get("object"==typeof e?e:{})};const _=O.prototype={createTracer:function(e,t){var r={},i=this,o="function"==typeof t;return(0,a.p)(f.xV,["API/createTracer/called"],void 0,n.K.metrics,R),h||(0,a.p)(S+"tracer",[(0,v.t)(),e,r],i,n.K.spa,R),function(){if(x.emit((o?"":"no-")+"fn-start",[(0,v.t)(),i,o],r),o)try{return t.apply(this,arguments)}catch(e){const t="string"==typeof e?new Error(e):e;throw x.emit("fn-err",[arguments,this,t],r),t}finally{x.emit("fn-end",[(0,v.t)()],r)}}}};function I(e,t,r,i){return function(){return(0,a.p)(f.xV,["API/"+t+"/called"],void 0,n.K.metrics,R),i&&(0,a.p)(e+t,[(0,v.t)(),...arguments],r?null:this,i,R),r?void 0:this}}function j(){r.e(478).then(r.bind(r,8778)).then((({setAPI:t})=>{t(e),(0,c.Ze)(e,"api")})).catch((e=>{(0,l.R)(27,e),R.abort()}))}return["actionText","setName","setAttribute","save","ignore","onEnd","getContext","end","get"].forEach((e=>{_[e]=I(S,e,void 0,h?n.K.softNav:n.K.spa)})),g.setCurrentRouteName=h?I(S,"routeName",void 0,n.K.softNav):I(A,"routeName",!0,n.K.spa),g.noticeError=function(t,r){"string"==typeof t&&(t=new Error(t)),(0,a.p)(f.xV,["API/noticeError/called"],void 0,n.K.metrics,R),(0,a.p)("err",[t,(0,v.t)(),!1,r,!!E[e]],void 0,n.K.jserrors,R)},d.RI?(0,u.GG)((()=>j()),!0):j(),g}(e.agentIdentifier,R,e.runSoftNavOverSpa)),void 0===e.exposed&&(e.exposed=k),I=!0}},8374:(e,t,r)=>{r.nc=(()=>{try{return document?.currentScript?.nonce}catch(e){}return""})()},860:(e,t,r)=>{"use strict";r.d(t,{K:()=>n,P:()=>i});const n={ajax:"ajax",genericEvents:"generic_events",jserrors:"jserrors",logging:"logging",metrics:"metrics",pageAction:"page_action",pageViewEvent:"page_view_event",pageViewTiming:"page_view_timing",sessionReplay:"session_replay",sessionTrace:"session_trace",softNav:"soft_navigations",spa:"spa"},i={[n.pageViewEvent]:1,[n.pageViewTiming]:2,[n.metrics]:3,[n.jserrors]:4,[n.spa]:5,[n.ajax]:6,[n.sessionTrace]:7,[n.softNav]:8,[n.sessionReplay]:9,[n.logging]:10,[n.genericEvents]:11}}},n={};function i(e){var t=n[e];if(void 0!==t)return t.exports;var o=n[e]={exports:{}};return r[e](o,o.exports,i),o.exports}i.m=r,i.d=(e,t)=>{for(var r in t)i.o(t,r)&&!i.o(e,r)&&Object.defineProperty(e,r,{enumerable:!0,get:t[r]})},i.f={},i.e=e=>Promise.all(Object.keys(i.f).reduce(((t,r)=>(i.f[r](e,t),t)),[])),i.u=e=>({212:"nr-spa-compressor",249:"nr-spa-recorder",478:"nr-spa"}[e]+"-1.272.0.min.js"),i.o=(e,t)=>Object.prototype.hasOwnProperty.call(e,t),e={},t="NRBA-1.272.0.PROD:",i.l=(r,n,o,a)=>{if(e[r])e[r].push(n);else{var s,c;if(void 0!==o)for(var u=document.getElementsByTagName("script"),d=0;d {s.onerror=s.onload=null,clearTimeout(p);var i=e[r];if(delete e[r],s.parentNode&&s.parentNode.removeChild(s),i&&i.forEach((e=>e(n))),t)return t(n)},p=setTimeout(h.bind(null,void 0,{type:"timeout",target:s}),12e4);s.onerror=h.bind(null,s.onerror),s.onload=h.bind(null,s.onload),c&&document.head.appendChild(s)}},i.r=e=>{"undefined"!=typeof Symbol&&Symbol.toStringTag&&Object.defineProperty(e,Symbol.toStringTag,{value:"Module"}),Object.defineProperty(e,"__esModule",{value:!0})},i.p="https://js-agent.newrelic.com/",(()=>{var e={38:0,788:0};i.f.j=(t,r)=>{var n=i.o(e,t)?e[t]:void 0;if(0!==n)if(n)r.push(n[2]);else{var o=new Promise(((r,i)=>n=e[t]=[r,i]));r.push(n[2]=o);var a=i.p+i.u(t),s=new Error;i.l(a,(r=>{if(i.o(e,t)&&(0!==(n=e[t])&&(e[t]=void 0),n)){var o=r&&("load"===r.type?"missing":r.type),a=r&&r.target&&r.target.src;s.message="Loading chunk "+t+" failed.\n("+o+": "+a+")",s.name="ChunkLoadError",s.type=o,s.request=a,n[1](s)}}),"chunk-"+t,t)}};var t=(t,r)=>{var n,o,[a,s,c]=r,u=0;if(a.some((t=>0!==e[t]))){for(n in s)i.o(s,n)&&(i.m[n]=s[n]);if(c)c(i)}for(t&&t(r);u {"use strict";i(8374);var e=i(944),t=i(6344),r=i(9566);class n{agentIdentifier;constructor(e=(0,r.LA)(16)){this.agentIdentifier=e}#e(t,...r){if("function"==typeof this.api?.[t])return this.api[t](...r);(0,e.R)(35,t)}addPageAction(e,t){return this.#e("addPageAction",e,t)}setPageViewName(e,t){return this.#e("setPageViewName",e,t)}setCustomAttribute(e,t,r){return this.#e("setCustomAttribute",e,t,r)}noticeError(e,t){return this.#e("noticeError",e,t)}setUserId(e){return this.#e("setUserId",e)}setApplicationVersion(e){return this.#e("setApplicationVersion",e)}setErrorHandler(e){return this.#e("setErrorHandler",e)}finished(e){return this.#e("finished",e)}addRelease(e,t){return this.#e("addRelease",e,t)}start(e){return this.#e("start",e)}recordReplay(){return this.#e(t.G4.RECORD)}pauseReplay(){return this.#e(t.G4.PAUSE)}addToTrace(e){return this.#e("addToTrace",e)}setCurrentRouteName(e){return this.#e("setCurrentRouteName",e)}interaction(){return this.#e("interaction")}log(e,t){return this.#e("log",e,t)}wrapLogger(e,t,r){return this.#e("wrapLogger",e,t,r)}}var o=i(860),a=i(9417);const s=Object.values(o.K);function c(e){const t={};return s.forEach((r=>{t[r]=function(e,t){return!0===(0,a.gD)(t,"".concat(e,".enabled"))}(r,e)})),t}var u=i(425);var d=i(1687),l=i(4234),f=i(5289),h=i(6154),p=i(5270),g=i(7767),m=i(6389);class v extends l.W{constructor(e,t,r=!0){super(e.agentIdentifier,t),this.auto=r,this.abortHandler=void 0,this.featAggregate=void 0,this.onAggregateImported=void 0,!1===e.init[this.featureName].autoStart&&(this.auto=!1),this.auto?(0,d.Ak)(e.agentIdentifier,t):this.ee.on("manual-start-all",(0,m.J)((()=>{(0,d.Ak)(e.agentIdentifier,this.featureName),this.auto=!0,this.importAggregator(e)})))}importAggregator(t,r={}){if(this.featAggregate||!this.auto)return;let n;this.onAggregateImported=new Promise((e=>{n=e}));const a=async()=>{let a;try{if((0,g.V)(this.agentIdentifier)){const{setupAgentSession:e}=await i.e(478).then(i.bind(i,6526));a=e(this.agentIdentifier)}}catch(t){(0,e.R)(20,t),this.ee.emit("internal-error",[t]),this.featureName===o.K.sessionReplay&&this.abortHandler?.()}try{if(t.sharedAggregator)await t.sharedAggregator;else{t.sharedAggregator=i.e(478).then(i.bind(i,5987));const{Aggregator:e}=await t.sharedAggregator;t.sharedAggregator=new e}if(!this.#t(this.featureName,a))return(0,d.Ze)(this.agentIdentifier,this.featureName),void n(!1);const{lazyFeatureLoader:e}=await i.e(478).then(i.bind(i,6103)),{Aggregate:o}=await e(this.featureName,"aggregate");this.featAggregate=new o(t,r),n(!0)}catch(t){(0,e.R)(34,t),this.abortHandler?.(),(0,d.Ze)(this.agentIdentifier,this.featureName,!0),n(!1),this.ee&&this.ee.abort()}};h.RI?(0,f.GG)((()=>a()),!0):a()}#t(e,t){switch(e){case o.K.sessionReplay:return(0,p.SR)(this.agentIdentifier)&&!!t;case o.K.sessionTrace:return!!t;default:return!0}}}var b=i(6630);class y extends v{static featureName=b.T;constructor(e,t=!0){super(e,b.T,t),this.importAggregator(e)}}var w=i(384);var R=i(9908),x=i(2843),T=i(3878),A=i(782),E=i(1863);class S extends v{static featureName=A.T;constructor(e,t=!0){super(e,A.T,t),h.RI&&((0,x.u)((()=>(0,R.p)("docHidden",[(0,E.t)()],void 0,A.T,this.ee)),!0),(0,T.sp)("pagehide",(()=>(0,R.p)("winPagehide",[(0,E.t)()],void 0,A.T,this.ee))),this.importAggregator(e))}}var N=i(3969);class O extends v{static featureName=N.TZ;constructor(e,t=!0){super(e,N.TZ,t),this.importAggregator(e)}}var _=i(6774),I=i(3304);class j{constructor(e,t,r,n,i){this.name="UncaughtError",this.message="string"==typeof e?e:(0,I.A)(e),this.sourceURL=t,this.line=r,this.column=n,this.__newrelic=i}}function P(e){return L(e)?e:new j(void 0!==e?.message?e.message:e,e?.filename||e?.sourceURL,e?.lineno||e?.line,e?.colno||e?.col,e?.__newrelic)}function C(e){const t="Unhandled Promise Rejection";if(!e?.reason)return;if(L(e.reason))try{return e.reason.message=t+": "+e.reason.message,P(e.reason)}catch(t){return P(e.reason)}const r=P(e.reason);return r.message=t+": "+r?.message,r}function k(e){if(e.error instanceof SyntaxError&&!/:\d+$/.test(e.error.stack?.trim())){const t=new j(e.message,e.filename,e.lineno,e.colno,e.error.__newrelic);return t.name=SyntaxError.name,t}return L(e.error)?e.error:P(e)}function L(e){return e instanceof Error&&!!e.stack}class H extends v{static featureName=_.T;#r=!1;constructor(e,r=!0){super(e,_.T,r);try{this.removeOnAbort=new AbortController}catch(e){}this.ee.on("internal-error",(e=>{this.abortHandler&&(0,R.p)("ierr",[P(e),(0,E.t)(),!0,{},this.#r],void 0,this.featureName,this.ee)})),this.ee.on(t.G4.REPLAY_RUNNING,(e=>{this.#r=e})),h.gm.addEventListener("unhandledrejection",(e=>{this.abortHandler&&(0,R.p)("err",[C(e),(0,E.t)(),!1,{unhandledPromiseRejection:1},this.#r],void 0,this.featureName,this.ee)}),(0,T.jT)(!1,this.removeOnAbort?.signal)),h.gm.addEventListener("error",(e=>{this.abortHandler&&(0,R.p)("err",[k(e),(0,E.t)(),!1,{},this.#r],void 0,this.featureName,this.ee)}),(0,T.jT)(!1,this.removeOnAbort?.signal)),this.abortHandler=this.#n,this.importAggregator(e)}#n(){this.removeOnAbort?.abort(),this.abortHandler=void 0}}var D=i(8990);let M=1;const K="nr@id";function U(e){const t=typeof e;return!e||"object"!==t&&"function"!==t?-1:e===h.gm?0:(0,D.I)(e,K,(function(){return M++}))}function V(e){if("string"==typeof e&&e.length)return e.length;if("object"==typeof e){if("undefined"!=typeof ArrayBuffer&&e instanceof ArrayBuffer&&e.byteLength)return e.byteLength;if("undefined"!=typeof Blob&&e instanceof Blob&&e.size)return e.size;if(!("undefined"!=typeof FormData&&e instanceof FormData))try{return(0,I.A)(e).length}catch(e){return}}}var G=i(8139),F=i(7836),B=i(3434);const W={},z=["open","send"];function q(t){var r=t||F.ee;const n=function(e){return(e||F.ee).get("xhr")}(r);if(W[n.debugId]++)return n;W[n.debugId]=1,(0,G.u)(r);var i=(0,B.YM)(n),o=h.gm.XMLHttpRequest,a=h.gm.MutationObserver,s=h.gm.Promise,c=h.gm.setInterval,u="readystatechange",d=["onload","onerror","onabort","onloadstart","onloadend","onprogress","ontimeout"],l=[],f=h.gm.XMLHttpRequest=function(t){const r=new o(t),a=n.context(r);try{n.emit("new-xhr",[r],a),r.addEventListener(u,(s=a,function(){var e=this;e.readyState>3&&!s.resolved&&(s.resolved=!0,n.emit("xhr-resolved",[],e)),i.inPlace(e,d,"fn-",y)}),(0,T.jT)(!1))}catch(t){(0,e.R)(15,t);try{n.emit("internal-error",[t])}catch(e){}}var s;return r};function p(e,t){i.inPlace(t,["onreadystatechange"],"fn-",y)}if(function(e,t){for(var r in e)t[r]=e[r]}(o,f),f.prototype=o.prototype,i.inPlace(f.prototype,z,"-xhr-",y),n.on("send-xhr-start",(function(e,t){p(e,t),function(e){l.push(e),a&&(g?g.then(b):c?c(b):(m=-m,v.data=m))}(t)})),n.on("open-xhr-start",p),a){var g=s&&s.resolve();if(!c&&!s){var m=1,v=document.createTextNode(m);new a(b).observe(v,{characterData:!0})}}else r.on("fn-end",(function(e){e[0]&&e[0].type===u||b()}));function b(){for(var e=0;e {r(J[ee],e,Y),r(Q[ee],e,Y)})),r(h.gm,"fetch",Z),t.on(Z+"end",(function(e,r){var n=this;if(r){var i=r.headers.get("content-length");null!==i&&(n.rxSize=i),t.emit(Z+"done",[null,r],n)}else t.emit(Z+"done",[e],n)})),t}var ne=i(7485),ie=i(5603);class oe{constructor(e){this.agentIdentifier=e}generateTracePayload(e){if(!this.shouldGenerateTrace(e))return null;var t=(0,ie.o)(this.agentIdentifier);if(!t)return null;var n=(t.accountID||"").toString()||null,i=(t.agentID||"").toString()||null,o=(t.trustKey||"").toString()||null;if(!n||!i)return null;var a=(0,r.ZF)(),s=(0,r.el)(),c=Date.now(),u={spanId:a,traceId:s,timestamp:c};return(e.sameOrigin||this.isAllowedOrigin(e)&&this.useTraceContextHeadersForCors())&&(u.traceContextParentHeader=this.generateTraceContextParentHeader(a,s),u.traceContextStateHeader=this.generateTraceContextStateHeader(a,c,n,i,o)),(e.sameOrigin&&!this.excludeNewrelicHeader()||!e.sameOrigin&&this.isAllowedOrigin(e)&&this.useNewrelicHeaderForCors())&&(u.newrelicHeader=this.generateTraceHeader(a,s,c,n,i,o)),u}generateTraceContextParentHeader(e,t){return"00-"+t+"-"+e+"-01"}generateTraceContextStateHeader(e,t,r,n,i){return i+"@nr=0-1-"+r+"-"+n+"-"+e+"----"+t}generateTraceHeader(e,t,r,n,i,o){if(!("function"==typeof h.gm?.btoa))return null;var a={v:[0,1],d:{ty:"Browser",ac:n,ap:i,id:e,tr:t,ti:r}};return o&&n!==o&&(a.d.tk=o),btoa((0,I.A)(a))}shouldGenerateTrace(e){return this.isDtEnabled()&&this.isAllowedOrigin(e)}isAllowedOrigin(e){var t=!1,r={};if((0,a.gD)(this.agentIdentifier,"distributed_tracing")&&(r=(0,a.D0)(this.agentIdentifier).distributed_tracing),e.sameOrigin)t=!0;else if(r.allowed_origins instanceof Array)for(var n=0;n (0,R.p)(e,t,r,n,this.ee);try{const e={xmlhttprequest:"xhr",fetch:"fetch",beacon:"beacon"};h.gm?.performance?.getEntriesByType("resource").forEach((t=>{if(t.initiatorType in e&&0!==t.responseStatus){const r={status:t.responseStatus},n={rxSize:t.transferSize,duration:Math.floor(t.duration),cbTime:0};he(r,t.name),this.handler("xhr",[r,n,t.startTime,t.responseEnd,e[t.initiatorType]],void 0,o.K.ajax)}}))}catch(e){}re(this.ee),q(this.ee),function(e,t,r,n){function i(e){var t=this;t.totalCbs=0,t.called=0,t.cbTime=0,t.end=x,t.ended=!1,t.xhrGuids={},t.lastSize=null,t.loadCaptureCalled=!1,t.params=this.params||{},t.metrics=this.metrics||{},e.addEventListener("load",(function(r){A(t,e)}),(0,T.jT)(!1)),h.lR||e.addEventListener("progress",(function(e){t.lastSize=e.loaded}),(0,T.jT)(!1))}function a(e){this.params={method:e[0]},he(this,e[1]),this.metrics={}}function s(t,r){e.loader_config.xpid&&this.sameOrigin&&r.setRequestHeader("X-NewRelic-ID",e.loader_config.xpid);var i=n.generateTracePayload(this.parsedOrigin);if(i){var o=!1;i.newrelicHeader&&(r.setRequestHeader("newrelic",i.newrelicHeader),o=!0),i.traceContextParentHeader&&(r.setRequestHeader("traceparent",i.traceContextParentHeader),i.traceContextStateHeader&&r.setRequestHeader("tracestate",i.traceContextStateHeader),o=!0),o&&(this.dt=i)}}function c(e,r){var n=this.metrics,i=e[0],o=this;if(n&&i){var a=V(i);a&&(n.txSize=a)}this.startTime=(0,E.t)(),this.body=i,this.listener=function(e){try{"abort"!==e.type||o.loadCaptureCalled||(o.params.aborted=!0),("load"!==e.type||o.called===o.totalCbs&&(o.onloadCalled||"function"!=typeof r.onload)&&"function"==typeof o.end)&&o.end(r)}catch(e){try{t.emit("internal-error",[e])}catch(e){}}};for(var s=0;s 1?e[1]=o:e.push(o)}}function s(e,t){var r=!1;return t.newrelicHeader&&(e.set("newrelic",t.newrelicHeader),r=!0),t.traceContextParentHeader&&(e.set("traceparent",t.traceContextParentHeader),t.traceContextStateHeader&&e.set("tracestate",t.traceContextStateHeader),r=!0),r}}function y(e,t){this.params={},this.metrics={},this.startTime=(0,E.t)(),this.dt=t,e.length>=1&&(this.target=e[0]),e.length>=2&&(this.opts=e[1]);var r,n=this.opts||{},i=this.target;"string"==typeof i?r=i:"object"==typeof i&&i instanceof de?r=i.url:h.gm?.URL&&"object"==typeof i&&i instanceof URL&&(r=i.href),he(this,r);var o=(""+(i&&i instanceof de&&i.method||n.method||"GET")).toUpperCase();this.params.method=o,this.body=n.body,this.txSize=V(n.body)||0}function w(e,t){if(this.endTime=(0,E.t)(),this.params||(this.params={}),(0,se.iW)(this.params))return;let n;this.params.status=t?t.status:0,"string"==typeof this.rxSize&&this.rxSize.length>0&&(n=+this.rxSize);const i={txSize:this.txSize,rxSize:n,duration:(0,E.t)()-this.startTime};r("xhr",[this.params,i,this.startTime,this.endTime,"fetch"],this,o.K.ajax)}function x(e){const t=this.params,n=this.metrics;if(!this.ended){this.ended=!0;for(let t=0;t {const t=e.getEntries();(0,R.p)(be,[t],void 0,o.K.sessionTrace,r)})),n.observe({type:ye,buffered:!0})}catch(e){}this.importAggregator(e,{resourceObserver:n})}}var Ne=i(2614);class Oe extends v{static featureName=t.TZ;#i;#o;constructor(e,r=!0){let n;super(e,t.TZ,r),this.replayRunning=!1,this.#o=e;try{n=JSON.parse(localStorage.getItem("".concat(Ne.H3,"_").concat(Ne.uh)))}catch(e){}(0,p.SR)(e.agentIdentifier)&&this.ee.on(t.G4.RECORD,(()=>this.#a())),this.#s(n)?(this.#i=n?.sessionReplayMode,this.#c()):this.importAggregator(e),this.ee.on("err",(e=>{this.replayRunning&&(this.errorNoticed=!0,(0,R.p)(t.G4.ERROR_DURING_REPLAY,[e],void 0,this.featureName,this.ee))})),this.ee.on(t.G4.REPLAY_RUNNING,(e=>{this.replayRunning=e}))}#s(e){return e&&(e.sessionReplayMode===Ne.g.FULL||e.sessionReplayMode===Ne.g.ERROR)||(0,p.Aw)(this.agentIdentifier)}#u=!1;async#c(e){if(!this.#u){this.#u=!0;try{const{Recorder:t}=await Promise.all([i.e(478),i.e(249)]).then(i.bind(i,2496));this.recorder??=new t({mode:this.#i,agentIdentifier:this.agentIdentifier,trigger:e,ee:this.ee}),this.recorder.startRecording(),this.abortHandler=this.recorder.stopRecording}catch(e){}this.importAggregator(this.#o,{recorder:this.recorder,errorNoticed:this.errorNoticed})}}#a(){this.featAggregate?this.featAggregate.mode!==Ne.g.FULL&&this.featAggregate.initializeRecording(Ne.g.FULL,!0):(this.#i=Ne.g.FULL,this.#c(t.Qb.API),this.recorder&&this.recorder.parent.mode!==Ne.g.FULL&&(this.recorder.parent.mode=Ne.g.FULL,this.recorder.stopRecording(),this.recorder.startRecording(),this.abortHandler=this.recorder.stopRecording))}}var _e=i(3962);class Ie extends v{static featureName=_e.TZ;constructor(e,t=!0){if(super(e,_e.TZ,t),!h.RI||!(0,w.dV)().o.MO)return;const r=me(this.ee),n=(0,G.u)(this.ee),i=()=>(0,R.p)("newURL",[(0,E.t)(),""+window.location],void 0,this.featureName,this.ee);r.on("pushState-end",i),r.on("replaceState-end",i);try{this.removeOnAbort=new AbortController}catch(e){}(0,T.sp)("popstate",(e=>(0,R.p)("newURL",[e.timeStamp,""+window.location],void 0,this.featureName,this.ee)),!0,this.removeOnAbort?.signal);let o=!1;const a=new((0,w.dV)().o.MO)(((e,t)=>{o||(o=!0,requestAnimationFrame((()=>{(0,R.p)("newDom",[(0,E.t)()],void 0,this.featureName,this.ee),o=!1})))})),s=(0,m.s)((e=>{(0,R.p)("newUIEvent",[e],void 0,this.featureName,this.ee),a.observe(document.body,{attributes:!0,childList:!0,subtree:!0,characterData:!0})}),100,{leading:!0});n.on("fn-start",(([e])=>{_e.tC.includes(e?.type)&&s(e)}));for(let e of _e.tC)document.addEventListener(e,(()=>{}));this.abortHandler=function(){this.removeOnAbort?.abort(),a.disconnect(),this.abortHandler=void 0},this.importAggregator(e,{domObserver:a})}}var je=i(7378);const Pe={},Ce=["appendChild","insertBefore","replaceChild"];function ke(e){const t=function(e){return(e||F.ee).get("jsonp")}(e);if(!h.RI||Pe[t.debugId])return t;Pe[t.debugId]=!0;var r=(0,B.YM)(t),n=/[?&](?:callback|cb)=([^&#]+)/,i=/(.*)\.([^.]+)/,o=/^(\w+)(\.|$)(.*)$/;function a(e,t){if(!e)return t;const r=e.match(o),n=r[1];return a(r[3],t[n])}return r.inPlace(Node.prototype,Ce,"dom-"),t.on("dom-start",(function(e){!function(e){if(!e||"string"!=typeof e.nodeName||"script"!==e.nodeName.toLowerCase())return;if("function"!=typeof e.addEventListener)return;var o=(s=e.src,c=s.match(n),c?c[1]:null);var s,c;if(!o)return;var u=function(e){var t=e.match(i);if(t&&t.length>=3)return{key:t[2],parent:a(t[1],window)};return{key:e,parent:window}}(o);if("function"!=typeof u.parent[u.key])return;var d={};function l(){t.emit("jsonp-end",[],d),e.removeEventListener("load",l,(0,T.jT)(!1)),e.removeEventListener("error",f,(0,T.jT)(!1))}function f(){t.emit("jsonp-error",[],d),t.emit("jsonp-end",[],d),e.removeEventListener("load",l,(0,T.jT)(!1)),e.removeEventListener("error",f,(0,T.jT)(!1))}r.inPlace(u.parent,[u.key],"cb-",d),e.addEventListener("load",l,(0,T.jT)(!1)),e.addEventListener("error",f,(0,T.jT)(!1)),t.emit("new-jsonp",[e.src],d)}(e[0])})),t}const Le={};function He(e){const t=function(e){return(e||F.ee).get("promise")}(e);if(Le[t.debugId])return t;Le[t.debugId]=!0;var r=t.context,n=(0,B.YM)(t),i=h.gm.Promise;return i&&function(){function e(r){var o=t.context(),a=n(r,"executor-",o,null,!1);const s=Reflect.construct(i,[a],e);return t.context(s).getCtx=function(){return o},s}h.gm.Promise=e,Object.defineProperty(e,"name",{value:"Promise"}),e.toString=function(){return i.toString()},Object.setPrototypeOf(e,i),["all","race"].forEach((function(r){const n=i[r];e[r]=function(e){let i=!1;[...e||[]].forEach((e=>{this.resolve(e).then(a("all"===r),a(!1))}));const o=n.apply(this,arguments);return o;function a(e){return function(){t.emit("propagate",[null,!i],o,!1,!1),i=i||!e}}}})),["resolve","reject"].forEach((function(r){const n=i[r];e[r]=function(e){const r=n.apply(this,arguments);return e!==r&&t.emit("propagate",[e,!0],r,!1,!1),r}})),e.prototype=i.prototype;const o=i.prototype.then;i.prototype.then=function(...e){var i=this,a=r(i);a.promise=i,e[0]=n(e[0],"cb-",a,null,!1),e[1]=n(e[1],"cb-",a,null,!1);const s=o.apply(this,e);return a.nextPromise=s,t.emit("propagate",[i,!0],s,!1,!1),s},i.prototype.then[B.Jt]=o,t.on("executor-start",(function(e){e[0]=n(e[0],"resolve-",this,null,!1),e[1]=n(e[1],"resolve-",this,null,!1)})),t.on("executor-err",(function(e,t,r){e[1](r)})),t.on("cb-end",(function(e,r,n){t.emit("propagate",[n,!0],this.nextPromise,!1,!1)})),t.on("propagate",(function(e,r,n){this.getCtx&&!r||(this.getCtx=function(){if(e instanceof Promise)var r=t.context(e);return r&&r.getCtx?r.getCtx():this})}))}(),t}const De={},Me="setTimeout",Ke="setInterval",Ue="clearTimeout",Ve="-start",Ge=[Me,"setImmediate",Ke,Ue,"clearImmediate"];function Fe(e){const t=function(e){return(e||F.ee).get("timer")}(e);if(De[t.debugId]++)return t;De[t.debugId]=1;var r=(0,B.YM)(t);return r.inPlace(h.gm,Ge.slice(0,2),Me+"-"),r.inPlace(h.gm,Ge.slice(2,3),Ke+"-"),r.inPlace(h.gm,Ge.slice(3),Ue+"-"),t.on(Ke+Ve,(function(e,t,n){e[0]=r(e[0],"fn-",null,n)})),t.on(Me+Ve,(function(e,t,n){this.method=n,this.timerDuration=isNaN(e[1])?0:+e[1],e[0]=r(e[0],"fn-",this,n)})),t}const Be={};function We(e){const t=function(e){return(e||F.ee).get("mutation")}(e);if(!h.RI||Be[t.debugId])return t;Be[t.debugId]=!0;var r=(0,B.YM)(t),n=h.gm.MutationObserver;return n&&(window.MutationObserver=function(e){return this instanceof n?new n(r(e,"fn-")):n.apply(this,arguments)},MutationObserver.prototype=n.prototype),t}const{TZ:ze,d3:qe,Kp:Ze,$p:Ye,wW:Xe,e5:Je,tH:Qe,uP:$e,rw:et,Lc:tt}=je;class rt extends v{static featureName=ze;constructor(e,t=!0){if(super(e,ze,t),!h.RI)return;try{this.removeOnAbort=new AbortController}catch(e){}let r,n=0;const i=this.ee.get("tracer"),o=ke(this.ee),a=He(this.ee),s=Fe(this.ee),c=q(this.ee),u=this.ee.get("events"),d=re(this.ee),l=me(this.ee),f=We(this.ee);function p(e,t){l.emit("newURL",[""+window.location,t])}function g(){n++,r=window.location.hash,this[$e]=(0,E.t)()}function m(){n--,window.location.hash!==r&&p(0,!0);var e=(0,E.t)();this[Je]=~~this[Je]+e-this[$e],this[tt]=e}function v(e,t){e.on(t,(function(){this[t]=(0,E.t)()}))}this.ee.on($e,g),a.on(et,g),o.on(et,g),this.ee.on(tt,m),a.on(Xe,m),o.on(Xe,m),this.ee.on("fn-err",((...t)=>{t[2]?.__newrelic?.[e.agentIdentifier]||(0,R.p)("function-err",[...t],void 0,this.featureName,this.ee)})),this.ee.buffer([$e,tt,"xhr-resolved"],this.featureName),u.buffer([$e],this.featureName),s.buffer(["setTimeout"+Ze,"clearTimeout"+qe,$e],this.featureName),c.buffer([$e,"new-xhr","send-xhr"+qe],this.featureName),d.buffer([Qe+qe,Qe+"-done",Qe+Ye+qe,Qe+Ye+Ze],this.featureName),l.buffer(["newURL"],this.featureName),f.buffer([$e],this.featureName),a.buffer(["propagate",et,Xe,"executor-err","resolve"+qe],this.featureName),i.buffer([$e,"no-"+$e],this.featureName),o.buffer(["new-jsonp","cb-start","jsonp-error","jsonp-end"],this.featureName),v(d,Qe+qe),v(d,Qe+"-done"),v(o,"new-jsonp"),v(o,"jsonp-end"),v(o,"cb-start"),l.on("pushState-end",p),l.on("replaceState-end",p),window.addEventListener("hashchange",p,(0,T.jT)(!0,this.removeOnAbort?.signal)),window.addEventListener("load",p,(0,T.jT)(!0,this.removeOnAbort?.signal)),window.addEventListener("popstate",(function(){p(0,n>1)}),(0,T.jT)(!0,this.removeOnAbort?.signal)),this.abortHandler=this.#n,this.importAggregator(e)}#n(){this.removeOnAbort?.abort(),this.abortHandler=void 0}}var nt=i(3333);class it extends v{static featureName=nt.TZ;constructor(e,t=!0){super(e,nt.TZ,t);const r=[e.init.page_action.enabled,e.init.performance.capture_marks,e.init.performance.capture_measures,e.init.user_actions.enabled];h.RI&&e.init.user_actions.enabled&&(nt.Zp.forEach((e=>(0,T.sp)(e,(e=>(0,R.p)("ua",[e],void 0,this.featureName,this.ee)),!0))),nt.qN.forEach((e=>(0,T.sp)(e,(e=>(0,R.p)("ua",[e],void 0,this.featureName,this.ee)))))),r.some((e=>e))?this.importAggregator(e):this.deregisterDrain()}}var ot=i(993),at=i(3785);class st extends v{static featureName=ot.TZ;constructor(e,t=!0){super(e,ot.TZ,t);const r=this.ee;this.ee.on("wrap-logger-end",(function([e]){const{level:t,customAttributes:n}=this;(0,at.R)(r,e,n,t)})),this.importAggregator(e)}}new class extends n{constructor(t,r){super(r),h.gm?(this.features={},(0,w.bQ)(this.agentIdentifier,this),this.desiredFeatures=new Set(t.features||[]),this.desiredFeatures.add(y),this.runSoftNavOverSpa=[...this.desiredFeatures].some((e=>e.featureName===o.K.softNav)),(0,u.j)(this,t,t.loaderType||"agent"),this.run()):(0,e.R)(21)}get config(){return{info:this.info,init:this.init,loader_config:this.loader_config,runtime:this.runtime}}run(){try{const t=c(this.agentIdentifier),r=[...this.desiredFeatures];r.sort(((e,t)=>o.P[e.featureName]-o.P[t.featureName])),r.forEach((r=>{if(!t[r.featureName]&&r.featureName!==o.K.pageViewEvent)return;if(this.runSoftNavOverSpa&&r.featureName===o.K.spa)return;if(!this.runSoftNavOverSpa&&r.featureName===o.K.softNav)return;const n=function(e){switch(e){case o.K.ajax:return[o.K.jserrors];case o.K.sessionTrace:return[o.K.ajax,o.K.pageViewEvent];case o.K.sessionReplay:return[o.K.sessionTrace];case o.K.pageViewTiming:return[o.K.pageViewEvent];default:return[]}}(r.featureName).filter((e=>!(e in this.features)));n.length>0&&(0,e.R)(36,{targetFeature:r.featureName,missingDependencies:n}),this.features[r.featureName]=new r(this)}))}catch(t){(0,e.R)(22,t);for(const e in this.features)this.features[e].abortHandler?.();const r=(0,w.Zm)();delete r.initializedAgents[this.agentIdentifier]?.api,delete r.initializedAgents[this.agentIdentifier]?.features,delete this.sharedAggregator;return r.ee.get(this.agentIdentifier).abort(),!1}}}({features:[fe,y,S,Se,Oe,O,H,it,st,Ie,rt],loaderType:"spa"})})()})();  window.NREUM||(NREUM={});NREUM.info = {"beacon":"bam.nr-data.net","errorBeacon":"bam.nr-data.net","licenseKey":"NRJS-bbf55bdee5f59ed6475","applicationID":"1567086821","transactionName":"MwAHY0oCWxFZV0RaXgpKJGRoTHENT1pcXFAALQRZXA9QEA==","queueTime":0,"applicationTime":110,"agent":"","atts":""}  (window.NREUM||(NREUM={})).init={privacy:{cookies_enabled:true},ajax:{deny_list:[]},distributed_tracing:{enabled:true}};(window.NREUM||(NREUM={})).loader_config={agentID:"1588829430",accountID:"3382936",trustKey:"2038175",xpid:"VwUPU19QDhABUVdVBwkBXlYA",licenseKey:"NRJS-bbf55bdee5f59ed6475",applicationID:"1567086821"};;/*! For license information please see nr-loader-spa-1.269.0.min.js.LICENSE.txt */
(()=>{var e,t,r={8122:(e,t,r)=>{"use strict";r.d(t,{a:()=>i});var n=r(944);function i(e,t){try{if(!e||"object"!=typeof e)return(0,n.R)(3);if(!t||"object"!=typeof t)return(0,n.R)(4);const r=Object.create(Object.getPrototypeOf(t),Object.getOwnPropertyDescriptors(t)),o=0===Object.keys(r).length?e:r;for(let a in o)if(void 0!==e[a])try{if(null===e[a]){r[a]=null;continue}Array.isArray(e[a])&&Array.isArray(t[a])?r[a]=Array.from(new Set([...e[a],...t[a]])):"object"==typeof e[a]&&"object"==typeof t[a]?r[a]=i(e[a],t[a]):r[a]=e[a]}catch(e){(0,n.R)(1,e)}return r}catch(e){(0,n.R)(2,e)}}},2555:(e,t,r)=>{"use strict";r.d(t,{Vp:()=>c,fn:()=>s,x1:()=>u});var n=r(384),i=r(8122);const o={beacon:n.NT.beacon,errorBeacon:n.NT.errorBeacon,licenseKey:void 0,applicationID:void 0,sa:void 0,queueTime:void 0,applicationTime:void 0,ttGuid:void 0,user:void 0,account:void 0,product:void 0,extra:void 0,jsAttributes:{},userAttributes:void 0,atts:void 0,transactionName:void 0,tNamePlain:void 0},a={};function s(e){try{const t=c(e);return!!t.licenseKey&&!!t.errorBeacon&&!!t.applicationID}catch(e){return!1}}function c(e){if(!e)throw new Error("All info objects require an agent identifier!");if(!a[e])throw new Error("Info for ".concat(e," was never set"));return a[e]}function u(e,t){if(!e)throw new Error("All info objects require an agent identifier!");a[e]=(0,i.a)(t,o);const r=(0,n.nY)(e);r&&(r.info=a[e])}},9417:(e,t,r)=>{"use strict";r.d(t,{D0:()=>h,gD:()=>p,xN:()=>g});var n=r(993);const i=e=>{if(!e||"string"!=typeof e)return!1;try{document.createDocumentFragment().querySelector(e)}catch{return!1}return!0};var o=r(2614),a=r(944),s=r(384),c=r(8122);const u="[data-nr-mask]",d=()=>{const e={mask_selector:"*",block_selector:"[data-nr-block]",mask_input_options:{color:!1,date:!1,"datetime-local":!1,email:!1,month:!1,number:!1,range:!1,search:!1,tel:!1,text:!1,time:!1,url:!1,week:!1,textarea:!1,select:!1,password:!0}};return{ajax:{deny_list:void 0,block_internal:!0,enabled:!0,harvestTimeSeconds:10,autoStart:!0},distributed_tracing:{enabled:void 0,exclude_newrelic_header:void 0,cors_use_newrelic_header:void 0,cors_use_tracecontext_headers:void 0,allowed_origins:void 0},feature_flags:[],generic_events:{enabled:!0,harvestTimeSeconds:30,autoStart:!0},harvest:{tooManyRequestsDelay:60},jserrors:{enabled:!0,harvestTimeSeconds:10,autoStart:!0},logging:{enabled:!0,harvestTimeSeconds:10,autoStart:!0,level:n.p_.INFO},metrics:{enabled:!0,autoStart:!0},obfuscate:void 0,page_action:{enabled:!0},user_actions:{enabled:!0},page_view_event:{enabled:!0,autoStart:!0},page_view_timing:{enabled:!0,harvestTimeSeconds:30,autoStart:!0},privacy:{cookies_enabled:!0},proxy:{assets:void 0,beacon:void 0},session:{expiresMs:o.wk,inactiveMs:o.BB},session_replay:{autoStart:!0,enabled:!1,harvestTimeSeconds:60,preload:!1,sampling_rate:10,error_sampling_rate:100,collect_fonts:!1,inline_images:!1,fix_stylesheets:!0,mask_all_inputs:!0,get mask_text_selector(){return e.mask_selector},set mask_text_selector(t){i(t)?e.mask_selector="".concat(t,",").concat(u):""===t||null===t?e.mask_selector=u:(0,a.R)(5,t)},get block_class(){return"nr-block"},get ignore_class(){return"nr-ignore"},get mask_text_class(){return"nr-mask"},get block_selector(){return e.block_selector},set block_selector(t){i(t)?e.block_selector+=",".concat(t):""!==t&&(0,a.R)(6,t)},get mask_input_options(){return e.mask_input_options},set mask_input_options(t){t&&"object"==typeof t?e.mask_input_options={...t,password:!0}:(0,a.R)(7,t)}},session_trace:{enabled:!0,harvestTimeSeconds:10,autoStart:!0},soft_navigations:{enabled:!0,harvestTimeSeconds:10,autoStart:!0},spa:{enabled:!0,harvestTimeSeconds:10,autoStart:!0},ssl:void 0}},l={},f="All configuration objects require an agent identifier!";function h(e){if(!e)throw new Error(f);if(!l[e])throw new Error("Configuration for ".concat(e," was never set"));return l[e]}function g(e,t){if(!e)throw new Error(f);l[e]=(0,c.a)(t,d());const r=(0,s.nY)(e);r&&(r.init=l[e])}function p(e,t){if(!e)throw new Error(f);var r=h(e);if(r){for(var n=t.split("."),i=0;i {"use strict";r.d(t,{a:()=>c,o:()=>s});var n=r(384),i=r(8122);const o={accountID:void 0,trustKey:void 0,agentID:void 0,licenseKey:void 0,applicationID:void 0,xpid:void 0},a={};function s(e){if(!e)throw new Error("All loader-config objects require an agent identifier!");if(!a[e])throw new Error("LoaderConfig for ".concat(e," was never set"));return a[e]}function c(e,t){if(!e)throw new Error("All loader-config objects require an agent identifier!");a[e]=(0,i.a)(t,o);const r=(0,n.nY)(e);r&&(r.loader_config=a[e])}},3371:(e,t,r)=>{"use strict";r.d(t,{V:()=>f,f:()=>l});var n=r(8122),i=r(384),o=r(6154),a=r(9324);let s=0;const c={buildEnv:a.F3,distMethod:a.Xs,version:a.xv,originTime:o.WN},u={customTransaction:void 0,disabled:!1,isolatedBacklog:!1,loaderType:void 0,maxBytes:3e4,onerror:void 0,origin:""+o.gm.location,ptid:void 0,releaseIds:{},appMetadata:{},session:void 0,denyList:void 0,timeKeeper:void 0,obfuscator:void 0},d={};function l(e){if(!e)throw new Error("All runtime objects require an agent identifier!");if(!d[e])throw new Error("Runtime for ".concat(e," was never set"));return d[e]}function f(e,t){if(!e)throw new Error("All runtime objects require an agent identifier!");d[e]={...(0,n.a)(t,u),...c},Object.hasOwnProperty.call(d[e],"harvestCount")||Object.defineProperty(d[e],"harvestCount",{get:()=>++s});const r=(0,i.nY)(e);r&&(r.runtime=d[e])}},9324:(e,t,r)=>{"use strict";r.d(t,{F3:()=>i,Xs:()=>o,Yq:()=>a,xv:()=>n});const n="1.269.0",i="PROD",o="CDN",a="2.0.0-alpha.12"},6154:(e,t,r)=>{"use strict";r.d(t,{A4:()=>s,OF:()=>d,RI:()=>i,Vr:()=>h,WN:()=>g,bv:()=>o,gm:()=>a,lR:()=>f,m:()=>u,mw:()=>c,sb:()=>l});var n=r(1863);const i="undefined"!=typeof window&&!!window.document,o="undefined"!=typeof WorkerGlobalScope&&("undefined"!=typeof self&&self instanceof WorkerGlobalScope&&self.navigator instanceof WorkerNavigator||"undefined"!=typeof globalThis&&globalThis instanceof WorkerGlobalScope&&globalThis.navigator instanceof WorkerNavigator),a=i?window:"undefined"!=typeof WorkerGlobalScope&&("undefined"!=typeof self&&self instanceof WorkerGlobalScope&&self||"undefined"!=typeof globalThis&&globalThis instanceof WorkerGlobalScope&&globalThis),s="complete"===a?.document?.readyState,c=Boolean("hidden"===a?.document?.visibilityState),u=""+a?.location,d=/iPad|iPhone|iPod/.test(a.navigator?.userAgent),l=d&&"undefined"==typeof SharedWorker,f=(()=>{const e=a.navigator?.userAgent?.match(/Firefox[/\s](\d+\.\d+)/);return Array.isArray(e)&&e.length>=2?+e[1]:0})(),h=!!a.navigator?.sendBeacon,g=Date.now()-(0,n.t)()},4777:(e,t,r)=>{"use strict";r.d(t,{J:()=>o});var n=r(944);const i={agentIdentifier:"",ee:void 0};class o{constructor(e){try{if("object"!=typeof e)return(0,n.R)(8);this.sharedContext={},Object.assign(this.sharedContext,i),Object.entries(e).forEach((([e,t])=>{Object.keys(i).includes(e)&&(this.sharedContext[e]=t)}))}catch(e){(0,n.R)(9,e)}}}},7295:(e,t,r)=>{"use strict";r.d(t,{Xv:()=>a,gX:()=>i,iW:()=>o});var n=[];function i(e){if(!e||o(e))return!1;if(0===n.length)return!0;for(var t=0;t 0?(o=r.substring(0,i),a=r.substring(i)):(o=r,a="");let[s]=o.split(":");n.push({hostname:s,pathname:a})}}function s(e,t){return!(e.length>t.length)&&t.indexOf(e)===t.length-e.length}function c(e,t){return 0===e.indexOf("/")&&(e=e.substring(1)),0===t.indexOf("/")&&(t=t.substring(1)),""===e||e===t}},1687:(e,t,r)=>{"use strict";r.d(t,{Ak:()=>c,Ze:()=>l,x3:()=>u});var n=r(7836),i=r(3606),o=r(860),a=r(2646);const s={};function c(e,t){const r={staged:!1,priority:o.P[t]||0};d(e),s[e].get(t)||s[e].set(t,r)}function u(e,t){e&&s[e]&&(s[e].get(t)&&s[e].delete(t),h(e,t,!1),s[e].size&&f(e))}function d(e){if(!e)throw new Error("agentIdentifier required");s[e]||(s[e]=new Map)}function l(e="",t="feature",r=!1){if(d(e),!e||!s[e].get(t)||r)return h(e,t);s[e].get(t).staged=!0,f(e)}function f(e){const t=Array.from(s[e]);t.every((([e,t])=>t.staged))&&(t.sort(((e,t)=>e[1].priority-t[1].priority)),t.forEach((([t])=>{s[e].delete(t),h(e,t)})))}function h(e,t,r=!0){const o=e?n.ee.get(e):n.ee,s=i.i.handlers;if(!o.aborted&&o.backlog&&s){if(r){const e=o.backlog[t],r=s[t];if(r){for(let t=0;e&&t {Object.values(t||{}).forEach((t=>{t[0]?.on&&t[0]?.context()instanceof a.y&&t[0].on(e,t[1])}))}))}}o.isolatedBacklog||delete s[t],o.backlog[t]=null,o.emit("drain-"+t,[])}}function g(e,t){var r=e[1];Object.values(t[r]||{}).forEach((t=>{var r=e[0];if(t[0]===r){var n=t[1],i=e[3],o=e[2];n.apply(i,o)}}))}},7836:(e,t,r)=>{"use strict";r.d(t,{P:()=>c,ee:()=>u});var n=r(384),i=r(8990),o=r(3371),a=r(2646),s=r(5607);const c="nr@context:".concat(s.W),u=function e(t,r){var n={},s={},d={},l=!1;try{l=16===r.length&&(0,o.f)(r).isolatedBacklog}catch(e){}var f={on:g,addEventListener:g,removeEventListener:function(e,t){var r=n[e];if(!r)return;for(var i=0;i {s[n]=t,t in r||(r[t]=[])}))},abort:function(){f._aborted=!0,Object.keys(f.backlog).forEach((e=>{delete f.backlog[e]}))},isBuffering:function(e){return!!v()[s[e]]},debugId:r,backlog:l?{}:t&&"object"==typeof t.backlog?t.backlog:{},isolatedBacklog:l};return Object.defineProperty(f,"aborted",{get:()=>{let e=f._aborted||!1;return e||(t&&(e=t.aborted),e)}}),f;function h(e){return e&&e instanceof a.y?e:e?(0,i.I)(e,c,(()=>new a.y(c))):new a.y(c)}function g(e,t){n[e]=p(e).concat(t)}function p(e){return n[e]||[]}function m(t){return d[t]=d[t]||e(f,t)}function v(){return f.backlog}}(void 0,"globalEE"),d=(0,n.Zm)();d.ee||(d.ee=u)},2646:(e,t,r)=>{"use strict";r.d(t,{y:()=>n});class n{constructor(e){this.contextId=e}}},9908:(e,t,r)=>{"use strict";r.d(t,{d:()=>n,p:()=>i});var n=r(7836).ee.get("handle");function i(e,t,r,i,o){o?(o.buffer([e],i),o.emit(e,t,r)):(n.buffer([e],i),n.emit(e,t,r))}},3606:(e,t,r)=>{"use strict";r.d(t,{i:()=>o});var n=r(9908);o.on=a;var i=o.handlers={};function o(e,t,r,o){a(o||n.d,i,e,t,r)}function a(e,t,r,i,o){o||(o="feature"),e||(e=n.d);var a=t[o]=t[o]||{};(a[r]=a[r]||[]).push([e,i])}},3878:(e,t,r)=>{"use strict";function n(e,t){return{capture:e,passive:!1,signal:t}}function i(e,t,r=!1,i){window.addEventListener(e,t,n(r,i))}function o(e,t,r=!1,i){document.addEventListener(e,t,n(r,i))}r.d(t,{DD:()=>o,jT:()=>n,sp:()=>i})},5607:(e,t,r)=>{"use strict";r.d(t,{W:()=>n});const n=(0,r(9566).bz)()},9566:(e,t,r)=>{"use strict";r.d(t,{LA:()=>s,ZF:()=>c,bz:()=>a,el:()=>u});var n=r(6154);const i="xxxxxxxx-xxxx-4xxx-yxxx-xxxxxxxxxxxx";function o(e,t){return e?15&e[t]:16*Math.random()|0}function a(){const e=n.gm?.crypto||n.gm?.msCrypto;let t,r=0;return e&&e.getRandomValues&&(t=e.getRandomValues(new Uint8Array(30))),i.split("").map((e=>"x"===e?o(t,r++).toString(16):"y"===e?(3&o()|8).toString(16):e)).join("")}function s(e){const t=n.gm?.crypto||n.gm?.msCrypto;let r,i=0;t&&t.getRandomValues&&(r=t.getRandomValues(new Uint8Array(e)));const a=[];for(var s=0;s {"use strict";r.d(t,{BB:()=>a,H3:()=>n,g:()=>u,iL:()=>c,tS:()=>s,uh:()=>i,wk:()=>o});const n="NRBA",i="SESSION",o=144e5,a=18e5,s={STARTED:"session-started",PAUSE:"session-pause",RESET:"session-reset",RESUME:"session-resume",UPDATE:"session-update"},c={SAME_TAB:"same-tab",CROSS_TAB:"cross-tab"},u={OFF:0,FULL:1,ERROR:2}},1863:(e,t,r)=>{"use strict";function n(){return Math.floor(performance.now())}r.d(t,{t:()=>n})},7485:(e,t,r)=>{"use strict";r.d(t,{D:()=>i});var n=r(6154);function i(e){if(0===(e||"").indexOf("data:"))return{protocol:"data"};try{const t=new URL(e,location.href),r={port:t.port,hostname:t.hostname,pathname:t.pathname,search:t.search,protocol:t.protocol.slice(0,t.protocol.indexOf(":")),sameOrigin:t.protocol===n.gm?.location?.protocol&&t.host===n.gm?.location?.host};return r.port&&""!==r.port||("http:"===t.protocol&&(r.port="80"),"https:"===t.protocol&&(r.port="443")),r.pathname&&""!==r.pathname?r.pathname.startsWith("/")||(r.pathname="/".concat(r.pathname)):r.pathname="/",r}catch(e){return{}}}},944:(e,t,r)=>{"use strict";function n(e,t){"function"==typeof console.debug&&console.debug("New Relic Warning: https://github.com/newrelic/newrelic-browser-agent/blob/main/docs/warning-codes.md#".concat(e),t)}r.d(t,{R:()=>n})},5284:(e,t,r)=>{"use strict";r.d(t,{t:()=>c,B:()=>s});var n=r(7836),i=r(6154);const o="newrelic";const a=new Set,s={};function c(e,t){const r=n.ee.get(t);s[t]??={},e&&"object"==typeof e&&(a.has(t)||(r.emit("rumresp",[e]),s[t]=e,a.add(t),function(e={}){try{i.gm.dispatchEvent(new CustomEvent(o,{detail:e}))}catch(e){}}({loaded:!0})))}},8990:(e,t,r)=>{"use strict";r.d(t,{I:()=>i});var n=Object.prototype.hasOwnProperty;function i(e,t,r){if(n.call(e,t))return e[t];var i=r();if(Object.defineProperty&&Object.keys)try{return Object.defineProperty(e,t,{value:i,writable:!0,enumerable:!1}),i}catch(e){}return e[t]=i,i}},6389:(e,t,r)=>{"use strict";function n(e,t=500,r={}){const n=r?.leading||!1;let i;return(...r)=>{n&&void 0===i&&(e.apply(this,r),i=setTimeout((()=>{i=clearTimeout(i)}),t)),n||(clearTimeout(i),i=setTimeout((()=>{e.apply(this,r)}),t))}}function i(e){let t=!1;return(...r)=>{t||(t=!0,e.apply(this,r))}}r.d(t,{J:()=>i,s:()=>n})},3304:(e,t,r)=>{"use strict";r.d(t,{A:()=>o});var n=r(7836);const i=()=>{const e=new WeakSet;return(t,r)=>{if("object"==typeof r&&null!==r){if(e.has(r))return;e.add(r)}return r}};function o(e){try{return JSON.stringify(e,i())??""}catch(e){try{n.ee.emit("internal-error",[e])}catch(e){}return""}}},5289:(e,t,r)=>{"use strict";r.d(t,{GG:()=>o,sB:()=>a});var n=r(3878);function i(){return"undefined"==typeof document||"complete"===document.readyState}function o(e,t){if(i())return e();(0,n.sp)("load",e,t)}function a(e){if(i())return e();(0,n.DD)("DOMContentLoaded",e)}},384:(e,t,r)=>{"use strict";r.d(t,{NT:()=>o,US:()=>d,Zm:()=>a,bQ:()=>c,dV:()=>s,nY:()=>u,pV:()=>l});var n=r(6154),i=r(1863);const o={beacon:"bam.nr-data.net",errorBeacon:"bam.nr-data.net"};function a(){return n.gm.NREUM||(n.gm.NREUM={}),void 0===n.gm.newrelic&&(n.gm.newrelic=n.gm.NREUM),n.gm.NREUM}function s(){let e=a();return e.o||(e.o={ST:n.gm.setTimeout,SI:n.gm.setImmediate,CT:n.gm.clearTimeout,XHR:n.gm.XMLHttpRequest,REQ:n.gm.Request,EV:n.gm.Event,PR:n.gm.Promise,MO:n.gm.MutationObserver,FETCH:n.gm.fetch,WS:n.gm.WebSocket}),e}function c(e,t){let r=a();r.initializedAgents??={},t.initializedAt={ms:(0,i.t)(),date:new Date},r.initializedAgents[e]=t}function u(e){let t=a();return t.initializedAgents?.[e]}function d(e,t){a()[e]=t}function l(){return function(){let e=a();const t=e.info||{};e.info={beacon:o.beacon,errorBeacon:o.errorBeacon,...t}}(),function(){let e=a();const t=e.init||{};e.init={...t}}(),s(),function(){let e=a();const t=e.loader_config||{};e.loader_config={...t}}(),a()}},2843:(e,t,r)=>{"use strict";r.d(t,{u:()=>i});var n=r(3878);function i(e,t=!1,r,i){(0,n.DD)("visibilitychange",(function(){if(t)return void("hidden"===document.visibilityState&&e());e(document.visibilityState)}),r,i)}},8139:(e,t,r)=>{"use strict";r.d(t,{u:()=>f});var n=r(7836),i=r(3434),o=r(8990),a=r(6154);const s={},c=a.gm.XMLHttpRequest,u="addEventListener",d="removeEventListener",l="nr@wrapped:".concat(n.P);function f(e){var t=function(e){return(e||n.ee).get("events")}(e);if(s[t.debugId]++)return t;s[t.debugId]=1;var r=(0,i.YM)(t,!0);function f(e){r.inPlace(e,[u,d],"-",g)}function g(e,t){return e[1]}return"getPrototypeOf"in Object&&(a.RI&&h(document,f),h(a.gm,f),h(c.prototype,f)),t.on(u+"-start",(function(e,t){var n=e[1];if(null!==n&&("function"==typeof n||"object"==typeof n)){var i=(0,o.I)(n,l,(function(){var e={object:function(){if("function"!=typeof n.handleEvent)return;return n.handleEvent.apply(n,arguments)},function:n}[typeof n];return e?r(e,"fn-",null,e.name||"anonymous"):n}));this.wrapped=e[1]=i}})),t.on(d+"-start",(function(e){e[1]=this.wrapped||e[1]})),t}function h(e,t,...r){let n=e;for(;"object"==typeof n&&!Object.prototype.hasOwnProperty.call(n,u);)n=Object.getPrototypeOf(n);n&&t(n,...r)}},3434:(e,t,r)=>{"use strict";r.d(t,{Jt:()=>o,YM:()=>c});var n=r(7836),i=r(5607);const o="nr@original:".concat(i.W);var a=Object.prototype.hasOwnProperty,s=!1;function c(e,t){return e||(e=n.ee),r.inPlace=function(e,t,n,i,o){n||(n="");const a="-"===n.charAt(0);for(let s=0;s {"use strict";r.d(t,{T:()=>n});const n=r(860).K.ajax},3333:(e,t,r)=>{"use strict";r.d(t,{TZ:()=>n,Zp:()=>i,mq:()=>s,nf:()=>a,qN:()=>o});const n=r(860).K.genericEvents,i=["auxclick","click","copy","keydown","paste","scrollend"],o=["focus","blur"],a=4,s=1e3},6774:(e,t,r)=>{"use strict";r.d(t,{T:()=>n});const n=r(860).K.jserrors},993:(e,t,r)=>{"use strict";r.d(t,{ET:()=>o,TZ:()=>a,p_:()=>i});var n=r(860);const i={ERROR:"ERROR",WARN:"WARN",INFO:"INFO",DEBUG:"DEBUG",TRACE:"TRACE"},o="log",a=n.K.logging},3785:(e,t,r)=>{"use strict";r.d(t,{R:()=>c,b:()=>u});var n=r(9908),i=r(1863),o=r(860),a=r(3969),s=r(993);function c(e,t,r={},c=s.p_.INFO){(0,n.p)(a.xV,["API/logging/".concat(c.toLowerCase(),"/called")],void 0,o.K.metrics,e),(0,n.p)(s.ET,[(0,i.t)(),t,r,c],void 0,o.K.logging,e)}function u(e){return"string"==typeof e&&Object.values(s.p_).some((t=>t===e.toUpperCase().trim()))}},3969:(e,t,r)=>{"use strict";r.d(t,{TZ:()=>n,XG:()=>s,rs:()=>i,xV:()=>a,z_:()=>o});const n=r(860).K.metrics,i="sm",o="cm",a="storeSupportabilityMetrics",s="storeEventMetrics"},6630:(e,t,r)=>{"use strict";r.d(t,{T:()=>n});const n=r(860).K.pageViewEvent},782:(e,t,r)=>{"use strict";r.d(t,{T:()=>n});const n=r(860).K.pageViewTiming},6344:(e,t,r)=>{"use strict";r.d(t,{BB:()=>d,G4:()=>o,Qb:()=>l,TZ:()=>i,Ug:()=>a,_s:()=>s,bc:()=>u,yP:()=>c});var n=r(2614);const i=r(860).K.sessionReplay,o={RECORD:"recordReplay",PAUSE:"pauseReplay",REPLAY_RUNNING:"replayRunning",ERROR_DURING_REPLAY:"errorDuringReplay"},a=.12,s={DomContentLoaded:0,Load:1,FullSnapshot:2,IncrementalSnapshot:3,Meta:4,Custom:5},c={[n.g.ERROR]:15e3,[n.g.FULL]:3e5,[n.g.OFF]:0},u={RESET:{message:"Session was reset",sm:"Reset"},IMPORT:{message:"Recorder failed to import",sm:"Import"},TOO_MANY:{message:"429: Too Many Requests",sm:"Too-Many"},TOO_BIG:{message:"Payload was too large",sm:"Too-Big"},CROSS_TAB:{message:"Session Entity was set to OFF on another tab",sm:"Cross-Tab"},ENTITLEMENTS:{message:"Session Replay is not allowed and will not be started",sm:"Entitlement"}},d=5e3,l={API:"api"}},5270:(e,t,r)=>{"use strict";r.d(t,{Aw:()=>c,CT:()=>u,SR:()=>s});var n=r(384),i=r(9417),o=r(7767),a=r(6154);function s(e){return!!(0,n.dV)().o.MO&&(0,o.V)(e)&&!0===(0,i.gD)(e,"session_trace.enabled")}function c(e){return!0===(0,i.gD)(e,"session_replay.preload")&&s(e)}function u(e,t){const r=t.correctAbsoluteTimestamp(e);return{originalTimestamp:e,correctedTimestamp:r,timestampDiff:e-r,originTime:a.WN,correctedOriginTime:t.correctedOriginTime,originTimeDiff:Math.floor(a.WN-t.correctedOriginTime)}}},3738:(e,t,r)=>{"use strict";r.d(t,{He:()=>i,Kp:()=>s,Lc:()=>u,Rz:()=>d,TZ:()=>n,bD:()=>o,d3:()=>a,jx:()=>l,uP:()=>c});const n=r(860).K.sessionTrace,i="bstResource",o="resource",a="-start",s="-end",c="fn"+a,u="fn"+s,d="pushState",l=1e3},3962:(e,t,r)=>{"use strict";r.d(t,{AM:()=>o,O2:()=>s,Qu:()=>c,TZ:()=>a,ih:()=>u,tC:()=>i});var n=r(860);const i=["click","keydown","submit"],o="api",a=n.K.softNav,s={INITIAL_PAGE_LOAD:"",ROUTE_CHANGE:1,UNSPECIFIED:2},c={INTERACTION:1,AJAX:2,CUSTOM_END:3,CUSTOM_TRACER:4},u={IP:"in progress",FIN:"finished",CAN:"cancelled"}},7378:(e,t,r)=>{"use strict";r.d(t,{$p:()=>R,BR:()=>b,Kp:()=>x,L3:()=>y,Lc:()=>c,NC:()=>o,SG:()=>d,TZ:()=>i,U6:()=>g,UT:()=>m,d3:()=>w,dT:()=>f,e5:()=>A,gx:()=>v,l9:()=>l,oW:()=>h,op:()=>p,rw:()=>u,tH:()=>E,uP:()=>s,wW:()=>T,xq:()=>a});var n=r(384);const i=r(860).K.spa,o=["click","submit","keypress","keydown","keyup","change"],a=999,s="fn-start",c="fn-end",u="cb-start",d="api-ixn-",l="remaining",f="interaction",h="spaNode",g="jsonpNode",p="fetch-start",m="fetch-done",v="fetch-body-",b="jsonp-end",y=(0,n.dV)().o.ST,w="-start",x="-end",R="-body",T="cb"+x,A="jsTime",E="fetch"},4234:(e,t,r)=>{"use strict";r.d(t,{W:()=>i});var n=r(7836);class i{constructor(e,t,r){this.agentIdentifier=e,this.aggregator=t,this.ee=n.ee.get(e),this.featureName=r,this.blocked=!1}}},7767:(e,t,r)=>{"use strict";r.d(t,{V:()=>o});var n=r(9417),i=r(6154);const o=e=>i.RI&&!0===(0,n.gD)(e,"privacy.cookies_enabled")},425:(e,t,r)=>{"use strict";r.d(t,{j:()=>j});var n=r(860),i=r(2555),o=r(3371),a=r(9908),s=r(7836),c=r(1687),u=r(5289),d=r(6154),l=r(944),f=r(3969),h=r(384),g=r(6344);const p=["setErrorHandler","finished","addToTrace","addRelease","addPageAction","setCurrentRouteName","setPageViewName","setCustomAttribute","interaction","noticeError","setUserId","setApplicationVersion","start",g.G4.RECORD,g.G4.PAUSE,"log","wrapLogger"],m=["setErrorHandler","finished","addToTrace","addRelease"];var v=r(1863),b=r(2614),y=r(993),w=r(3785),x=r(2646),R=r(3434);function T(e,t,r,n){if("object"!=typeof t||!t||"string"!=typeof r||!r||"function"!=typeof t[r])return(0,l.R)(29);const i=function(e){return(e||s.ee).get("logger")}(e),o=(0,R.YM)(i),a=new x.y(s.P);return a.level=n.level,a.customAttributes=n.customAttributes,o.inPlace(t,[r],"wrap-logger-",a),i}function A(){const e=(0,h.pV)();p.forEach((t=>{e[t]=(...r)=>function(t,...r){let n=[];return Object.values(e.initializedAgents).forEach((e=>{e&&e.api?e.exposed&&e.api[t]&&n.push(e.api[t](...r)):(0,l.R)(38,t)})),n.length>1?n:n[0]}(t,...r)}))}const E={};var S=r(9417),N=r(5603),O=r(5284);const I=e=>{const t=e.startsWith("http");e+="/",r.p=t?e:"https://"+e};let _=!1;function j(e,t={},p,x){let{init:R,info:j,loader_config:P,runtime:C={},exposed:k=!0}=t;C.loaderType=p;const L=(0,h.pV)();j||(R=L.init,j=L.info,P=L.loader_config),(0,S.xN)(e.agentIdentifier,R||{}),(0,N.a)(e.agentIdentifier,P||{}),j.jsAttributes??={},d.bv&&(j.jsAttributes.isWorker=!0),(0,i.x1)(e.agentIdentifier,j);const D=(0,S.D0)(e.agentIdentifier),H=[j.beacon,j.errorBeacon];_||(D.proxy.assets&&(I(D.proxy.assets),H.push(D.proxy.assets)),D.proxy.beacon&&H.push(D.proxy.beacon),A(),(0,h.US)("activatedFeatures",O.B),e.runSoftNavOverSpa&&=!0===D.soft_navigations.enabled&&D.feature_flags.includes("soft_nav")),C.denyList=[...D.ajax.deny_list||[],...D.ajax.block_internal?H:[]],C.ptid=e.agentIdentifier,(0,o.V)(e.agentIdentifier,C),e.ee=s.ee.get(e.agentIdentifier),void 0===e.api&&(e.api=function(e,t,h=!1){t||(0,c.Ak)(e,"api");const p={};var x=s.ee.get(e),R=x.get("tracer");E[e]=b.g.OFF,x.on(g.G4.REPLAY_RUNNING,(t=>{E[e]=t}));var A="api-",S=A+"ixn-";function N(t,r,n,o){const a=(0,i.Vp)(e);return null===r?delete a.jsAttributes[t]:(0,i.x1)(e,{...a,jsAttributes:{...a.jsAttributes,[t]:r}}),_(A,n,!0,o||null===r?"session":void 0)(t,r)}function O(){}p.log=function(e,{customAttributes:t={},level:r=y.p_.INFO}={}){(0,a.p)(f.xV,["API/log/called"],void 0,n.K.metrics,x),(0,w.R)(x,e,t,r)},p.wrapLogger=(e,t,{customAttributes:r={},level:i=y.p_.INFO}={})=>{(0,a.p)(f.xV,["API/wrapLogger/called"],void 0,n.K.metrics,x),T(x,e,t,{customAttributes:r,level:i})},m.forEach((e=>{p[e]=_(A,e,!0,"api")})),p.addPageAction=_(A,"addPageAction",!0,n.K.genericEvents),p.setPageViewName=function(t,r){if("string"==typeof t)return"/"!==t.charAt(0)&&(t="/"+t),(0,o.f)(e).customTransaction=(r||"http://custom.transaction")+t,_(A,"setPageViewName",!0)()},p.setCustomAttribute=function(e,t,r=!1){if("string"==typeof e){if(["string","number","boolean"].includes(typeof t)||null===t)return N(e,t,"setCustomAttribute",r);(0,l.R)(40,typeof t)}else(0,l.R)(39,typeof e)},p.setUserId=function(e){if("string"==typeof e||null===e)return N("enduser.id",e,"setUserId",!0);(0,l.R)(41,typeof e)},p.setApplicationVersion=function(e){if("string"==typeof e||null===e)return N("application.version",e,"setApplicationVersion",!1);(0,l.R)(42,typeof e)},p.start=()=>{try{(0,a.p)(f.xV,["API/start/called"],void 0,n.K.metrics,x),x.emit("manual-start-all")}catch(e){(0,l.R)(23,e)}},p[g.G4.RECORD]=function(){(0,a.p)(f.xV,["API/recordReplay/called"],void 0,n.K.metrics,x),(0,a.p)(g.G4.RECORD,[],void 0,n.K.sessionReplay,x)},p[g.G4.PAUSE]=function(){(0,a.p)(f.xV,["API/pauseReplay/called"],void 0,n.K.metrics,x),(0,a.p)(g.G4.PAUSE,[],void 0,n.K.sessionReplay,x)},p.interaction=function(e){return(new O).get("object"==typeof e?e:{})};const I=O.prototype={createTracer:function(e,t){var r={},i=this,o="function"==typeof t;return(0,a.p)(f.xV,["API/createTracer/called"],void 0,n.K.metrics,x),h||(0,a.p)(S+"tracer",[(0,v.t)(),e,r],i,n.K.spa,x),function(){if(R.emit((o?"":"no-")+"fn-start",[(0,v.t)(),i,o],r),o)try{return t.apply(this,arguments)}catch(e){const t="string"==typeof e?new Error(e):e;throw R.emit("fn-err",[arguments,this,t],r),t}finally{R.emit("fn-end",[(0,v.t)()],r)}}}};function _(e,t,r,i){return function(){return(0,a.p)(f.xV,["API/"+t+"/called"],void 0,n.K.metrics,x),i&&(0,a.p)(e+t,[(0,v.t)(),...arguments],r?null:this,i,x),r?void 0:this}}function j(){r.e(478).then(r.bind(r,8778)).then((({setAPI:t})=>{t(e),(0,c.Ze)(e,"api")})).catch((e=>{(0,l.R)(27,e),x.abort()}))}return["actionText","setName","setAttribute","save","ignore","onEnd","getContext","end","get"].forEach((e=>{I[e]=_(S,e,void 0,h?n.K.softNav:n.K.spa)})),p.setCurrentRouteName=h?_(S,"routeName",void 0,n.K.softNav):_(A,"routeName",!0,n.K.spa),p.noticeError=function(t,r){"string"==typeof t&&(t=new Error(t)),(0,a.p)(f.xV,["API/noticeError/called"],void 0,n.K.metrics,x),(0,a.p)("err",[t,(0,v.t)(),!1,r,!!E[e]],void 0,n.K.jserrors,x)},d.RI?(0,u.GG)((()=>j()),!0):j(),p}(e.agentIdentifier,x,e.runSoftNavOverSpa)),void 0===e.exposed&&(e.exposed=k),_=!0}},8374:(e,t,r)=>{r.nc=(()=>{try{return document?.currentScript?.nonce}catch(e){}return""})()},860:(e,t,r)=>{"use strict";r.d(t,{K:()=>n,P:()=>i});const n={ajax:"ajax",genericEvents:"generic_events",jserrors:"jserrors",logging:"logging",metrics:"metrics",pageAction:"page_action",pageViewEvent:"page_view_event",pageViewTiming:"page_view_timing",sessionReplay:"session_replay",sessionTrace:"session_trace",softNav:"soft_navigations",spa:"spa"},i={[n.pageViewEvent]:1,[n.pageViewTiming]:2,[n.metrics]:3,[n.jserrors]:4,[n.spa]:5,[n.ajax]:6,[n.sessionTrace]:7,[n.softNav]:8,[n.sessionReplay]:9,[n.logging]:10,[n.genericEvents]:11}}},n={};function i(e){var t=n[e];if(void 0!==t)return t.exports;var o=n[e]={exports:{}};return r[e](o,o.exports,i),o.exports}i.m=r,i.d=(e,t)=>{for(var r in t)i.o(t,r)&&!i.o(e,r)&&Object.defineProperty(e,r,{enumerable:!0,get:t[r]})},i.f={},i.e=e=>Promise.all(Object.keys(i.f).reduce(((t,r)=>(i.f[r](e,t),t)),[])),i.u=e=>({212:"nr-spa-compressor",249:"nr-spa-recorder",478:"nr-spa"}[e]+"-1.269.0.min.js"),i.o=(e,t)=>Object.prototype.hasOwnProperty.call(e,t),e={},t="NRBA-1.269.0.PROD:",i.l=(r,n,o,a)=>{if(e[r])e[r].push(n);else{var s,c;if(void 0!==o)for(var u=document.getElementsByTagName("script"),d=0;d {s.onerror=s.onload=null,clearTimeout(g);var i=e[r];if(delete e[r],s.parentNode&&s.parentNode.removeChild(s),i&&i.forEach((e=>e(n))),t)return t(n)},g=setTimeout(h.bind(null,void 0,{type:"timeout",target:s}),12e4);s.onerror=h.bind(null,s.onerror),s.onload=h.bind(null,s.onload),c&&document.head.appendChild(s)}},i.r=e=>{"undefined"!=typeof Symbol&&Symbol.toStringTag&&Object.defineProperty(e,Symbol.toStringTag,{value:"Module"}),Object.defineProperty(e,"__esModule",{value:!0})},i.p="https://js-agent.newrelic.com/",(()=>{var e={38:0,788:0};i.f.j=(t,r)=>{var n=i.o(e,t)?e[t]:void 0;if(0!==n)if(n)r.push(n[2]);else{var o=new Promise(((r,i)=>n=e[t]=[r,i]));r.push(n[2]=o);var a=i.p+i.u(t),s=new Error;i.l(a,(r=>{if(i.o(e,t)&&(0!==(n=e[t])&&(e[t]=void 0),n)){var o=r&&("load"===r.type?"missing":r.type),a=r&&r.target&&r.target.src;s.message="Loading chunk "+t+" failed.\n("+o+": "+a+")",s.name="ChunkLoadError",s.type=o,s.request=a,n[1](s)}}),"chunk-"+t,t)}};var t=(t,r)=>{var n,o,[a,s,c]=r,u=0;if(a.some((t=>0!==e[t]))){for(n in s)i.o(s,n)&&(i.m[n]=s[n]);if(c)c(i)}for(t&&t(r);u {"use strict";i(8374);var e=i(944),t=i(6344),r=i(9566);class n{agentIdentifier;constructor(e=(0,r.LA)(16)){this.agentIdentifier=e}#e(t,...r){if("function"==typeof this.api?.[t])return this.api[t](...r);(0,e.R)(35,t)}addPageAction(e,t){return this.#e("addPageAction",e,t)}setPageViewName(e,t){return this.#e("setPageViewName",e,t)}setCustomAttribute(e,t,r){return this.#e("setCustomAttribute",e,t,r)}noticeError(e,t){return this.#e("noticeError",e,t)}setUserId(e){return this.#e("setUserId",e)}setApplicationVersion(e){return this.#e("setApplicationVersion",e)}setErrorHandler(e){return this.#e("setErrorHandler",e)}finished(e){return this.#e("finished",e)}addRelease(e,t){return this.#e("addRelease",e,t)}start(e){return this.#e("start",e)}recordReplay(){return this.#e(t.G4.RECORD)}pauseReplay(){return this.#e(t.G4.PAUSE)}addToTrace(e){return this.#e("addToTrace",e)}setCurrentRouteName(e){return this.#e("setCurrentRouteName",e)}interaction(){return this.#e("interaction")}log(e,t){return this.#e("log",e,t)}wrapLogger(e,t,r){return this.#e("wrapLogger",e,t,r)}}var o=i(860),a=i(9417);const s=Object.values(o.K);function c(e){const t={};return s.forEach((r=>{t[r]=function(e,t){return!0===(0,a.gD)(t,"".concat(e,".enabled"))}(r,e)})),t}var u=i(425);var d=i(1687),l=i(4234),f=i(5289),h=i(6154),g=i(5270),p=i(7767),m=i(6389);class v extends l.W{constructor(e,t,r,n=!0){super(e,t,r),this.auto=n,this.abortHandler=void 0,this.featAggregate=void 0,this.onAggregateImported=void 0,!1===(0,a.gD)(this.agentIdentifier,"".concat(this.featureName,".autoStart"))&&(this.auto=!1),this.auto?(0,d.Ak)(e,r):this.ee.on("manual-start-all",(0,m.J)((()=>{(0,d.Ak)(this.agentIdentifier,this.featureName),this.auto=!0,this.importAggregator()})))}importAggregator(t={}){if(this.featAggregate||!this.auto)return;let r;this.onAggregateImported=new Promise((e=>{r=e}));const n=async()=>{let n;try{if((0,p.V)(this.agentIdentifier)){const{setupAgentSession:e}=await i.e(478).then(i.bind(i,6526));n=e(this.agentIdentifier)}}catch(t){(0,e.R)(20,t),this.ee.emit("internal-error",[t]),this.featureName===o.K.sessionReplay&&this.abortHandler?.()}try{if(!this.#t(this.featureName,n))return(0,d.Ze)(this.agentIdentifier,this.featureName),void r(!1);const{lazyFeatureLoader:e}=await i.e(478).then(i.bind(i,6103)),{Aggregate:o}=await e(this.featureName,"aggregate");this.featAggregate=new o(this.agentIdentifier,this.aggregator,t),r(!0)}catch(t){(0,e.R)(34,t),this.abortHandler?.(),(0,d.Ze)(this.agentIdentifier,this.featureName,!0),r(!1),this.ee&&this.ee.abort()}};h.RI?(0,f.GG)((()=>n()),!0):n()}#t(e,t){switch(e){case o.K.sessionReplay:return(0,g.SR)(this.agentIdentifier)&&!!t;case o.K.sessionTrace:return!!t;default:return!0}}}var b=i(6630);class y extends v{static featureName=b.T;constructor(e,t,r=!0){super(e,t,b.T,r),this.importAggregator()}}var w=i(4777);class x extends w.J{constructor(e){super(e),this.aggregatedData={}}store(e,t,r,n,i){var o=this.getBucket(e,t,r,i);return o.metrics=function(e,t){t||(t={count:0});return t.count+=1,Object.entries(e||{}).forEach((([e,r])=>{t[e]=R(r,t[e])})),t}(n,o.metrics),o}merge(e,t,r,n,i){var o=this.getBucket(e,t,n,i);if(o.metrics){var a=o.metrics;a.count+=r.count,Object.keys(r||{}).forEach((e=>{if("count"!==e){var t=a[e],n=r[e];n&&!n.c?a[e]=R(n.t,t):a[e]=function(e,t){if(!t)return e;t.c||(t=T(t.t));return t.min=Math.min(e.min,t.min),t.max=Math.max(e.max,t.max),t.t+=e.t,t.sos+=e.sos,t.c+=e.c,t}(n,a[e])}}))}else o.metrics=r}storeMetric(e,t,r,n){var i=this.getBucket(e,t,r);return i.stats=R(n,i.stats),i}getBucket(e,t,r,n){this.aggregatedData[e]||(this.aggregatedData[e]={});var i=this.aggregatedData[e][t];return i||(i=this.aggregatedData[e][t]={params:r||{}},n&&(i.custom=n)),i}get(e,t){return t?this.aggregatedData[e]&&this.aggregatedData[e][t]:this.aggregatedData[e]}take(e){for(var t={},r="",n=!1,i=0;i t.max&&(t.max=e),e (0,E.p)("docHidden",[(0,I.t)()],void 0,O.T,this.ee)),!0),(0,N.sp)("pagehide",(()=>(0,E.p)("winPagehide",[(0,I.t)()],void 0,O.T,this.ee))),this.importAggregator())}}var j=i(3969);class P extends v{static featureName=j.TZ;constructor(e,t,r=!0){super(e,t,j.TZ,r),this.importAggregator()}}var C=i(6774),k=i(3304);class L{constructor(e,t,r,n,i){this.name="UncaughtError",this.message="string"==typeof e?e:(0,k.A)(e),this.sourceURL=t,this.line=r,this.column=n,this.__newrelic=i}}function D(e){return K(e)?e:new L(void 0!==e?.message?e.message:e,e?.filename||e?.sourceURL,e?.lineno||e?.line,e?.colno||e?.col,e?.__newrelic)}function H(e){let t="Unhandled Promise Rejection";if(K(e?.reason))try{return e.reason.message=t+": "+e.reason.message,D(e.reason)}catch(t){return D(e.reason)}if(void 0===e.reason)return D(t);const r=D(e.reason);return r.message=t+": "+r?.message,r}function M(e){if(e.error instanceof SyntaxError&&!/:\d+$/.test(e.error.stack?.trim())){const t=new L(e.message,e.filename,e.lineno,e.colno,e.error.__newrelic);return t.name=SyntaxError.name,t}return K(e.error)?e.error:D(e)}function K(e){return e instanceof Error&&!!e.stack}class U extends v{static featureName=C.T;#r=!1;constructor(e,r,n=!0){super(e,r,C.T,n);try{this.removeOnAbort=new AbortController}catch(e){}this.ee.on("internal-error",(e=>{this.abortHandler&&(0,E.p)("ierr",[D(e),(0,I.t)(),!0,{},this.#r],void 0,this.featureName,this.ee)})),this.ee.on(t.G4.REPLAY_RUNNING,(e=>{this.#r=e})),h.gm.addEventListener("unhandledrejection",(e=>{this.abortHandler&&(0,E.p)("err",[H(e),(0,I.t)(),!1,{unhandledPromiseRejection:1},this.#r],void 0,this.featureName,this.ee)}),(0,N.jT)(!1,this.removeOnAbort?.signal)),h.gm.addEventListener("error",(e=>{this.abortHandler&&(0,E.p)("err",[M(e),(0,I.t)(),!1,{},this.#r],void 0,this.featureName,this.ee)}),(0,N.jT)(!1,this.removeOnAbort?.signal)),this.abortHandler=this.#n,this.importAggregator()}#n(){this.removeOnAbort?.abort(),this.abortHandler=void 0}}var V=i(5603),G=i(8990);let B=1;const F="nr@id";function W(e){const t=typeof e;return!e||"object"!==t&&"function"!==t?-1:e===h.gm?0:(0,G.I)(e,F,(function(){return B++}))}function z(e){if("string"==typeof e&&e.length)return e.length;if("object"==typeof e){if("undefined"!=typeof ArrayBuffer&&e instanceof ArrayBuffer&&e.byteLength)return e.byteLength;if("undefined"!=typeof Blob&&e instanceof Blob&&e.size)return e.size;if(!("undefined"!=typeof FormData&&e instanceof FormData))try{return(0,k.A)(e).length}catch(e){return}}}var q=i(8139),Z=i(7836),Y=i(3434);const X={},J=["open","send"];function Q(t){var r=t||Z.ee;const n=function(e){return(e||Z.ee).get("xhr")}(r);if(X[n.debugId]++)return n;X[n.debugId]=1,(0,q.u)(r);var i=(0,Y.YM)(n),o=h.gm.XMLHttpRequest,a=h.gm.MutationObserver,s=h.gm.Promise,c=h.gm.setInterval,u="readystatechange",d=["onload","onerror","onabort","onloadstart","onloadend","onprogress","ontimeout"],l=[],f=h.gm.XMLHttpRequest=function(t){const r=new o(t),a=n.context(r);try{n.emit("new-xhr",[r],a),r.addEventListener(u,(s=a,function(){var e=this;e.readyState>3&&!s.resolved&&(s.resolved=!0,n.emit("xhr-resolved",[],e)),i.inPlace(e,d,"fn-",y)}),(0,N.jT)(!1))}catch(t){(0,e.R)(15,t);try{n.emit("internal-error",[t])}catch(e){}}var s;return r};function g(e,t){i.inPlace(t,["onreadystatechange"],"fn-",y)}if(function(e,t){for(var r in e)t[r]=e[r]}(o,f),f.prototype=o.prototype,i.inPlace(f.prototype,J,"-xhr-",y),n.on("send-xhr-start",(function(e,t){g(e,t),function(e){l.push(e),a&&(p?p.then(b):c?c(b):(m=-m,v.data=m))}(t)})),n.on("open-xhr-start",g),a){var p=s&&s.resolve();if(!c&&!s){var m=1,v=document.createTextNode(m);new a(b).observe(v,{characterData:!0})}}else r.on("fn-end",(function(e){e[0]&&e[0].type===u||b()}));function b(){for(var e=0;e {r(ne[oe],e,te),r(ie[oe],e,te)})),r(h.gm,"fetch",ee),t.on(ee+"end",(function(e,r){var n=this;if(r){var i=r.headers.get("content-length");null!==i&&(n.rxSize=i),t.emit(ee+"done",[null,r],n)}else t.emit(ee+"done",[e],n)})),t}var ce=i(7485);class ue{constructor(e){this.agentIdentifier=e}generateTracePayload(e){if(!this.shouldGenerateTrace(e))return null;var t=(0,V.o)(this.agentIdentifier);if(!t)return null;var n=(t.accountID||"").toString()||null,i=(t.agentID||"").toString()||null,o=(t.trustKey||"").toString()||null;if(!n||!i)return null;var a=(0,r.ZF)(),s=(0,r.el)(),c=Date.now(),u={spanId:a,traceId:s,timestamp:c};return(e.sameOrigin||this.isAllowedOrigin(e)&&this.useTraceContextHeadersForCors())&&(u.traceContextParentHeader=this.generateTraceContextParentHeader(a,s),u.traceContextStateHeader=this.generateTraceContextStateHeader(a,c,n,i,o)),(e.sameOrigin&&!this.excludeNewrelicHeader()||!e.sameOrigin&&this.isAllowedOrigin(e)&&this.useNewrelicHeaderForCors())&&(u.newrelicHeader=this.generateTraceHeader(a,s,c,n,i,o)),u}generateTraceContextParentHeader(e,t){return"00-"+t+"-"+e+"-01"}generateTraceContextStateHeader(e,t,r,n,i){return i+"@nr=0-1-"+r+"-"+n+"-"+e+"----"+t}generateTraceHeader(e,t,r,n,i,o){if(!("function"==typeof h.gm?.btoa))return null;var a={v:[0,1],d:{ty:"Browser",ac:n,ap:i,id:e,tr:t,ti:r}};return o&&n!==o&&(a.d.tk=o),btoa((0,k.A)(a))}shouldGenerateTrace(e){return this.isDtEnabled()&&this.isAllowedOrigin(e)}isAllowedOrigin(e){var t=!1,r={};if((0,a.gD)(this.agentIdentifier,"distributed_tracing")&&(r=(0,a.D0)(this.agentIdentifier).distributed_tracing),e.sameOrigin)t=!0;else if(r.allowed_origins instanceof Array)for(var n=0;n (0,E.p)(e,t,r,n,this.ee);try{const e={xmlhttprequest:"xhr",fetch:"fetch",beacon:"beacon"};h.gm?.performance?.getEntriesByType("resource").forEach((t=>{if(t.initiatorType in e&&0!==t.responseStatus){const r={status:t.responseStatus},n={rxSize:t.transferSize,duration:Math.floor(t.duration),cbTime:0};ve(r,t.name),this.handler("xhr",[r,n,t.startTime,t.responseEnd,e[t.initiatorType]],void 0,o.K.ajax)}}))}catch(e){}se(this.ee),Q(this.ee),function(e,t,r,n){function i(e){var t=this;t.totalCbs=0,t.called=0,t.cbTime=0,t.end=x,t.ended=!1,t.xhrGuids={},t.lastSize=null,t.loadCaptureCalled=!1,t.params=this.params||{},t.metrics=this.metrics||{},e.addEventListener("load",(function(r){R(t,e)}),(0,N.jT)(!1)),h.lR||e.addEventListener("progress",(function(e){t.lastSize=e.loaded}),(0,N.jT)(!1))}function a(e){this.params={method:e[0]},ve(this,e[1]),this.metrics={}}function s(t,r){var i=(0,V.o)(e);i.xpid&&this.sameOrigin&&r.setRequestHeader("X-NewRelic-ID",i.xpid);var o=n.generateTracePayload(this.parsedOrigin);if(o){var a=!1;o.newrelicHeader&&(r.setRequestHeader("newrelic",o.newrelicHeader),a=!0),o.traceContextParentHeader&&(r.setRequestHeader("traceparent",o.traceContextParentHeader),o.traceContextStateHeader&&r.setRequestHeader("tracestate",o.traceContextStateHeader),a=!0),a&&(this.dt=o)}}function c(e,r){var n=this.metrics,i=e[0],o=this;if(n&&i){var a=z(i);a&&(n.txSize=a)}this.startTime=(0,I.t)(),this.body=i,this.listener=function(e){try{"abort"!==e.type||o.loadCaptureCalled||(o.params.aborted=!0),("load"!==e.type||o.called===o.totalCbs&&(o.onloadCalled||"function"!=typeof r.onload)&&"function"==typeof o.end)&&o.end(r)}catch(e){try{t.emit("internal-error",[e])}catch(e){}}};for(var s=0;s 1?e[1]=o:e.push(o)}}function s(e,t){var r=!1;return t.newrelicHeader&&(e.set("newrelic",t.newrelicHeader),r=!0),t.traceContextParentHeader&&(e.set("traceparent",t.traceContextParentHeader),t.traceContextStateHeader&&e.set("tracestate",t.traceContextStateHeader),r=!0),r}}function y(e,t){this.params={},this.metrics={},this.startTime=(0,I.t)(),this.dt=t,e.length>=1&&(this.target=e[0]),e.length>=2&&(this.opts=e[1]);var r,n=this.opts||{},i=this.target;"string"==typeof i?r=i:"object"==typeof i&&i instanceof ge?r=i.url:h.gm?.URL&&"object"==typeof i&&i instanceof URL&&(r=i.href),ve(this,r);var o=(""+(i&&i instanceof ge&&i.method||n.method||"GET")).toUpperCase();this.params.method=o,this.body=n.body,this.txSize=z(n.body)||0}function w(e,t){if(this.endTime=(0,I.t)(),this.params||(this.params={}),(0,le.iW)(this.params))return;let n;this.params.status=t?t.status:0,"string"==typeof this.rxSize&&this.rxSize.length>0&&(n=+this.rxSize);const i={txSize:this.txSize,rxSize:n,duration:(0,I.t)()-this.startTime};r("xhr",[this.params,i,this.startTime,this.endTime,"fetch"],this,o.K.ajax)}function x(e){const t=this.params,n=this.metrics;if(!this.ended){this.ended=!0;for(let t=0;t {const t=e.getEntries();(0,E.p)(Re,[t],void 0,o.K.sessionTrace,n)})),i.observe({type:Te,buffered:!0})}catch(e){}this.importAggregator({resourceObserver:i})}}var je=i(2614);class Pe extends v{static featureName=t.TZ;#i;constructor(e,r,n=!0){let i;super(e,r,t.TZ,n),this.replayRunning=!1;try{i=JSON.parse(localStorage.getItem("".concat(je.H3,"_").concat(je.uh)))}catch(e){}(0,g.SR)(e)&&this.ee.on(t.G4.RECORD,(()=>this.#o())),this.#a(i)?(this.#i=i?.sessionReplayMode,this.#s()):this.importAggregator(),this.ee.on("err",(e=>{this.replayRunning&&(this.errorNoticed=!0,(0,E.p)(t.G4.ERROR_DURING_REPLAY,[e],void 0,this.featureName,this.ee))})),this.ee.on(t.G4.REPLAY_RUNNING,(e=>{this.replayRunning=e}))}#a(e){return e&&(e.sessionReplayMode===je.g.FULL||e.sessionReplayMode===je.g.ERROR)||(0,g.Aw)(this.agentIdentifier)}#c=!1;async#s(e){if(!this.#c){this.#c=!0;try{const{Recorder:t}=await Promise.all([i.e(478),i.e(249)]).then(i.bind(i,2496));this.recorder??=new t({mode:this.#i,agentIdentifier:this.agentIdentifier,trigger:e,ee:this.ee}),this.recorder.startRecording(),this.abortHandler=this.recorder.stopRecording}catch(e){}this.importAggregator({recorder:this.recorder,errorNoticed:this.errorNoticed})}}#o(){this.featAggregate?this.featAggregate.mode!==je.g.FULL&&this.featAggregate.initializeRecording(je.g.FULL,!0):(this.#i=je.g.FULL,this.#s(t.Qb.API),this.recorder&&this.recorder.parent.mode!==je.g.FULL&&(this.recorder.parent.mode=je.g.FULL,this.recorder.stopRecording(),this.recorder.startRecording(),this.abortHandler=this.recorder.stopRecording))}}var Ce=i(3962);class ke extends v{static featureName=Ce.TZ;constructor(e,t,r=!0){if(super(e,t,Ce.TZ,r),!h.RI||!(0,A.dV)().o.MO)return;const n=we(this.ee),i=(0,q.u)(this.ee),o=()=>(0,E.p)("newURL",[(0,I.t)(),""+window.location],void 0,this.featureName,this.ee);n.on("pushState-end",o),n.on("replaceState-end",o);try{this.removeOnAbort=new AbortController}catch(e){}(0,N.sp)("popstate",(e=>(0,E.p)("newURL",[e.timeStamp,""+window.location],void 0,this.featureName,this.ee)),!0,this.removeOnAbort?.signal);let a=!1;const s=new((0,A.dV)().o.MO)(((e,t)=>{a||(a=!0,requestAnimationFrame((()=>{(0,E.p)("newDom",[(0,I.t)()],void 0,this.featureName,this.ee),a=!1})))})),c=(0,m.s)((e=>{(0,E.p)("newUIEvent",[e],void 0,this.featureName,this.ee),s.observe(document.body,{attributes:!0,childList:!0,subtree:!0,characterData:!0})}),100,{leading:!0});i.on("fn-start",(([e])=>{Ce.tC.includes(e?.type)&&c(e)}));for(let e of Ce.tC)document.addEventListener(e,(()=>{}));this.abortHandler=function(){this.removeOnAbort?.abort(),s.disconnect(),this.abortHandler=void 0},this.importAggregator({domObserver:s})}}var Le=i(7378);const De={},He=["appendChild","insertBefore","replaceChild"];function Me(e){const t=function(e){return(e||Z.ee).get("jsonp")}(e);if(!h.RI||De[t.debugId])return t;De[t.debugId]=!0;var r=(0,Y.YM)(t),n=/[?&](?:callback|cb)=([^&#]+)/,i=/(.*)\.([^.]+)/,o=/^(\w+)(\.|$)(.*)$/;function a(e,t){if(!e)return t;const r=e.match(o),n=r[1];return a(r[3],t[n])}return r.inPlace(Node.prototype,He,"dom-"),t.on("dom-start",(function(e){!function(e){if(!e||"string"!=typeof e.nodeName||"script"!==e.nodeName.toLowerCase())return;if("function"!=typeof e.addEventListener)return;var o=(s=e.src,c=s.match(n),c?c[1]:null);var s,c;if(!o)return;var u=function(e){var t=e.match(i);if(t&&t.length>=3)return{key:t[2],parent:a(t[1],window)};return{key:e,parent:window}}(o);if("function"!=typeof u.parent[u.key])return;var d={};function l(){t.emit("jsonp-end",[],d),e.removeEventListener("load",l,(0,N.jT)(!1)),e.removeEventListener("error",f,(0,N.jT)(!1))}function f(){t.emit("jsonp-error",[],d),t.emit("jsonp-end",[],d),e.removeEventListener("load",l,(0,N.jT)(!1)),e.removeEventListener("error",f,(0,N.jT)(!1))}r.inPlace(u.parent,[u.key],"cb-",d),e.addEventListener("load",l,(0,N.jT)(!1)),e.addEventListener("error",f,(0,N.jT)(!1)),t.emit("new-jsonp",[e.src],d)}(e[0])})),t}const Ke={};function Ue(e){const t=function(e){return(e||Z.ee).get("promise")}(e);if(Ke[t.debugId])return t;Ke[t.debugId]=!0;var r=t.context,n=(0,Y.YM)(t),i=h.gm.Promise;return i&&function(){function e(r){var o=t.context(),a=n(r,"executor-",o,null,!1);const s=Reflect.construct(i,[a],e);return t.context(s).getCtx=function(){return o},s}h.gm.Promise=e,Object.defineProperty(e,"name",{value:"Promise"}),e.toString=function(){return i.toString()},Object.setPrototypeOf(e,i),["all","race"].forEach((function(r){const n=i[r];e[r]=function(e){let i=!1;[...e||[]].forEach((e=>{this.resolve(e).then(a("all"===r),a(!1))}));const o=n.apply(this,arguments);return o;function a(e){return function(){t.emit("propagate",[null,!i],o,!1,!1),i=i||!e}}}})),["resolve","reject"].forEach((function(r){const n=i[r];e[r]=function(e){const r=n.apply(this,arguments);return e!==r&&t.emit("propagate",[e,!0],r,!1,!1),r}})),e.prototype=i.prototype;const o=i.prototype.then;i.prototype.then=function(...e){var i=this,a=r(i);a.promise=i,e[0]=n(e[0],"cb-",a,null,!1),e[1]=n(e[1],"cb-",a,null,!1);const s=o.apply(this,e);return a.nextPromise=s,t.emit("propagate",[i,!0],s,!1,!1),s},i.prototype.then[Y.Jt]=o,t.on("executor-start",(function(e){e[0]=n(e[0],"resolve-",this,null,!1),e[1]=n(e[1],"resolve-",this,null,!1)})),t.on("executor-err",(function(e,t,r){e[1](r)})),t.on("cb-end",(function(e,r,n){t.emit("propagate",[n,!0],this.nextPromise,!1,!1)})),t.on("propagate",(function(e,r,n){this.getCtx&&!r||(this.getCtx=function(){if(e instanceof Promise)var r=t.context(e);return r&&r.getCtx?r.getCtx():this})}))}(),t}const Ve={},Ge="setTimeout",Be="setInterval",Fe="clearTimeout",We="-start",ze=[Ge,"setImmediate",Be,Fe,"clearImmediate"];function qe(e){const t=function(e){return(e||Z.ee).get("timer")}(e);if(Ve[t.debugId]++)return t;Ve[t.debugId]=1;var r=(0,Y.YM)(t);return r.inPlace(h.gm,ze.slice(0,2),Ge+"-"),r.inPlace(h.gm,ze.slice(2,3),Be+"-"),r.inPlace(h.gm,ze.slice(3),Fe+"-"),t.on(Be+We,(function(e,t,n){e[0]=r(e[0],"fn-",null,n)})),t.on(Ge+We,(function(e,t,n){this.method=n,this.timerDuration=isNaN(e[1])?0:+e[1],e[0]=r(e[0],"fn-",this,n)})),t}const Ze={};function Ye(e){const t=function(e){return(e||Z.ee).get("mutation")}(e);if(!h.RI||Ze[t.debugId])return t;Ze[t.debugId]=!0;var r=(0,Y.YM)(t),n=h.gm.MutationObserver;return n&&(window.MutationObserver=function(e){return this instanceof n?new n(r(e,"fn-")):n.apply(this,arguments)},MutationObserver.prototype=n.prototype),t}const{TZ:Xe,d3:Je,Kp:Qe,$p:$e,wW:et,e5:tt,tH:rt,uP:nt,rw:it,Lc:ot}=Le;class at extends v{static featureName=Xe;constructor(e,t,r=!0){if(super(e,t,Xe,r),!h.RI)return;try{this.removeOnAbort=new AbortController}catch(e){}let n,i=0;const o=this.ee.get("tracer"),a=Me(this.ee),s=Ue(this.ee),c=qe(this.ee),u=Q(this.ee),d=this.ee.get("events"),l=se(this.ee),f=we(this.ee),g=Ye(this.ee);function p(e,t){f.emit("newURL",[""+window.location,t])}function m(){i++,n=window.location.hash,this[nt]=(0,I.t)()}function v(){i--,window.location.hash!==n&&p(0,!0);var e=(0,I.t)();this[tt]=~~this[tt]+e-this[nt],this[ot]=e}function b(e,t){e.on(t,(function(){this[t]=(0,I.t)()}))}this.ee.on(nt,m),s.on(it,m),a.on(it,m),this.ee.on(ot,v),s.on(et,v),a.on(et,v),this.ee.on("fn-err",((...t)=>{t[2]?.__newrelic?.[e]||(0,E.p)("function-err",[...t],void 0,this.featureName,this.ee)})),this.ee.buffer([nt,ot,"xhr-resolved"],this.featureName),d.buffer([nt],this.featureName),c.buffer(["setTimeout"+Qe,"clearTimeout"+Je,nt],this.featureName),u.buffer([nt,"new-xhr","send-xhr"+Je],this.featureName),l.buffer([rt+Je,rt+"-done",rt+$e+Je,rt+$e+Qe],this.featureName),f.buffer(["newURL"],this.featureName),g.buffer([nt],this.featureName),s.buffer(["propagate",it,et,"executor-err","resolve"+Je],this.featureName),o.buffer([nt,"no-"+nt],this.featureName),a.buffer(["new-jsonp","cb-start","jsonp-error","jsonp-end"],this.featureName),b(l,rt+Je),b(l,rt+"-done"),b(a,"new-jsonp"),b(a,"jsonp-end"),b(a,"cb-start"),f.on("pushState-end",p),f.on("replaceState-end",p),window.addEventListener("hashchange",p,(0,N.jT)(!0,this.removeOnAbort?.signal)),window.addEventListener("load",p,(0,N.jT)(!0,this.removeOnAbort?.signal)),window.addEventListener("popstate",(function(){p(0,i>1)}),(0,N.jT)(!0,this.removeOnAbort?.signal)),this.abortHandler=this.#n,this.importAggregator()}#n(){this.removeOnAbort?.abort(),this.abortHandler=void 0}}var st=i(3333);class ct extends v{static featureName=st.TZ;constructor(e,t,r=!0){super(e,t,st.TZ,r);const n=(0,a.D0)(this.agentIdentifier),i=[n.page_action.enabled,n.user_actions.enabled];h.RI&&n.user_actions.enabled&&(st.Zp.forEach((e=>(0,N.sp)(e,(e=>(0,E.p)("ua",[e],void 0,this.featureName,this.ee)),!0))),st.qN.forEach((e=>(0,N.sp)(e,(e=>(0,E.p)("ua",[e],void 0,this.featureName,this.ee)))))),i.some((e=>e))?this.importAggregator():(0,d.x3)(this.agentIdentifier,this.featureName)}}var ut=i(993),dt=i(3785);class lt extends v{static featureName=ut.TZ;constructor(e,t,r=!0){super(e,t,ut.TZ,r);const n=this.ee;this.ee.on("wrap-logger-end",(function([e]){const{level:t,customAttributes:r}=this;(0,dt.R)(n,e,r,t)})),this.importAggregator()}}new class extends n{constructor(t,r){super(r),h.gm?(this.sharedAggregator=new x({agentIdentifier:this.agentIdentifier}),this.features={},(0,A.bQ)(this.agentIdentifier,this),this.desiredFeatures=new Set(t.features||[]),this.desiredFeatures.add(y),this.runSoftNavOverSpa=[...this.desiredFeatures].some((e=>e.featureName===o.K.softNav)),(0,u.j)(this,t,t.loaderType||"agent"),this.run()):(0,e.R)(21)}get config(){return{info:this.info,init:this.init,loader_config:this.loader_config,runtime:this.runtime}}run(){try{const t=c(this.agentIdentifier),r=[...this.desiredFeatures];r.sort(((e,t)=>o.P[e.featureName]-o.P[t.featureName])),r.forEach((r=>{if(!t[r.featureName]&&r.featureName!==o.K.pageViewEvent)return;if(this.runSoftNavOverSpa&&r.featureName===o.K.spa)return;if(!this.runSoftNavOverSpa&&r.featureName===o.K.softNav)return;const n=function(e){switch(e){case o.K.ajax:return[o.K.jserrors];case o.K.sessionTrace:return[o.K.ajax,o.K.pageViewEvent];case o.K.sessionReplay:return[o.K.sessionTrace];case o.K.pageViewTiming:return[o.K.pageViewEvent];default:return[]}}(r.featureName).filter((e=>!(e in this.features)));n.length>0&&(0,e.R)(36,{targetFeature:r.featureName,missingDependencies:n}),this.features[r.featureName]=new r(this.agentIdentifier,this.sharedAggregator)}))}catch(t){(0,e.R)(22,t);for(const e in this.features)this.features[e].abortHandler?.();const r=(0,A.Zm)();delete r.initializedAgents[this.agentIdentifier]?.api,delete r.initializedAgents[this.agentIdentifier]?.features,delete this.sharedAggregator;return r.ee.get(this.agentIdentifier).abort(),!1}}}({features:[me,y,_,_e,Pe,P,U,ct,lt,ke,at],loaderType:"spa"})})()})();  Codings created by RQDA.  Created by  RQDA  at 2021-02-03 08:33:06  
 Annual Plan   Attitude   Biodegradable waste   Budget allocation   Check list   Collection   Discussion   Environmental effect    Follow up by Governemnt    HCWM officer    Hospital permises    PPE set supply    Periodic waste collection   Pharmaceutical waste   Placenta Pit   Policy    Provision of salary   Purchasing Mechanism    Salary   Segregation   Social Security   Staff Transfer   Train manpower   Transportation    Vaccine   disposal   facilitation for training    hazardious waste   infectious waste   insurance   knowledge   laboratory waste   lack of supervision   manpower   member for annual planing    planing implimentation    post exposure service   recording    solid waste    staff hiring    stakeholder   sterilization    storage   use of color bucket       2 Codings of  "Annual Plan"  from 2 files.       KII 3 [7125:7187]     There is routine for waste management but not the annual plan.  Back      KII 4 [5036:5144]     Annual plan is not prepared. But we are managing wastes daily. It would be better if we prepare daily plans.  Back       4 Codings of  "Attitude"  from 2 files.       KII 5 [3959:4045]     behavior change and the disclipline of the top to bottom workers is equally important.  Back      KII 6 [2581:2692]     Yes, there is. If staffs shows positive attitude and take responsibility it will be easier in waste management.  Back      KII 6 [2902:2949]     Attitude is very poor towards waste management.  Back      KII 6 [6749:6888]     n Terai, tobacco is commonly used which is one of the major source of waste. People using tobacco should be told to dispose waste properly.  Back       11 Codings of  "Biodegradable waste"  from 9 files.       KII 1 [ 240: 258]     papers, food waste  Back      KII 10 [ 456: 461]     paper  Back      KII 10 [1053:1061]     Placenta  Back      KII 2 [2238:2244]     papers  Back      KII 3 [ 573: 588]      water soluble   Back      KII 3 [ 840: 887]     paper and foods are normal and degradable waste  Back      KII 4 [ 733: 748]     foods, biscuits  Back      KII 5 [ 240: 251]     food wastes  Back      KII 6 [ 360: 366]     Papers  Back      KII 7 [ 262: 267]     paper  Back      KII 8 [ 364: 374]     food waste  Back       16 Codings of  "Budget allocation"  from 8 files.       KII 1 [ 5740: 5773]     allocate budgets to the hospital.  Back      KII 1 [ 5848: 5876]     budgets for human resources.  Back      KII 1 [ 1390: 1426]     Another big issue is lack of budget.  Back      KII 2 [11492:11506]      one is budget  Back      KII 2 [ 5898: 6022]     We also need extra budget for that, Hospital does not have its own budget which is in municipality. Budget has one problem;   Back      KII 3 [ 4818: 4873]     other problem is lack of sufficient finance and budget.  Back      KII 4 [ 3864: 4040]     Hospital development committee has recruited the sweepers but the committee has no money. It is difficult to pay money to the sweepers though they are working in minimum salary  Back      KII 5 [ 1972: 2015]     budget inefficiencies for waste management.  Back      KII 5 [ 2321: 2340]     budget to implement  Back      KII 5 [ 5896: 5967]     Thus we expect allocation of the budget from the provincial government.  Back      KII 7 [ 3582: 3652]      We expect proper guidelines and routine budget for waste management.   Back      KII 8 [ 3425: 3498]     allocating budgets would help in implementing waste management guidelines  Back      KII 8 [ 5398: 5461]      local government can help in logistics and budget allocation .  Back      KII 8 [ 2912: 2967]     Also, there is lack of budget to implement guidelines.   Back      KII 9 [ 6526: 6638]      if hospital get a Yearly budget categorization, we have somehow managed 15% fund to spend on waste management.   Back      KII 9 [10005:10129]     In the context of our hospital our municipality has budgeted of 25% for waste management which is really a good thing to do.  Back       5 Codings of  "Check list"  from 5 files.       KII 3 [5669:5692]     No, it is not practiced  Back      KII 4 [3589:3632]     But there is no manpower so it is not done.  Back      KII 5 [3094:3137]     No, we don’t have any checklist or logbook.  Back      KII 7 [2666:2709]     we don’t have any checklist for monitoring.  Back      KII 8 [3876:3963]      do have checklist for the sweepers who mark the checklist after every duty completion   Back       5 Codings of  "Collection"  from 5 files.       KII 10 [3054:3112]      As there is collection of waste, issues has been raised.   Back      KII 2 [3519:3616]     It was segregate and not used. Expect taking them to municipality they are dumping in same place.  Back      KII 3 [1398:1563]     If the waste is not graded well, they also feel problem in performing their duties and we collect the waste at one place which is carried by vehicle of Municipality.  Back      KII 7 [ 526: 548]     related to collection   Back      KII 9 [1457:1502]      there is a problem in center collection side  Back       1 Coding of  "Discussion"  from 1 file.       KII 6 [3270:3412]     It is very important to organize meetings to mitigate waste management problems because patients will get affected if hospital is not cleaned.  Back       2 Codings of  "Environmental effect "  from 1 file.       KII 6 [5405:5493]     Unhygienic environment in hospital may transmit various diseases to patients and staffs.  Back      KII 6 [5523:5601]     Dirty environment in hospital creates negative impression on patients as well.  Back       1 Coding of  "Follow up by Governemnt "  from 1 file.       KII 2 [11584:11650]     If follow up made the government then it will be fully implemented  Back       5 Codings of  "HCWM officer "  from 4 files.       KII 10 [5699:5959]     we all one responsible for waste management in hospital. So, we all supervise that waste management process. No one has assigned for monitoring, In our hospital one sister observed the waste management process. But no one has officially assigned for that jobs.  Back      KII 4 [1839:2007]     We have published an advertisement for the post of medical superintendent for healthcare waste management. But no one applied for the post and our advertisement failed.  Back      KII 4 [2979:3098]     He assists medical superintendent, gives presentation. But the post is vacant as no one responded to the advertisement.  Back      KII 6 [1334:1353]     lack of proper site  Back      KII 7 [1924:2041]     For waste management we can council the patient and their parties. For this the hospital can appoint  a focal person.  Back       5 Codings of  "Hospital permises "  from 3 files.       KII 3 [2205:2260]     shortage of place all activities could not perform well  Back      KII 3 [3063:3216]     Waste is littered 24 hours which should be collected and now premises is so large, in which time and how much time is required to check is not possible.   Back      KII 3 [4751:4786]     The main problems are lack of space  Back      KII 4 [7125:7205]     Lack of space and proper delivery are the problem in managing healthcare wastes.  Back      KII 5 [1084:1167]     Also, the placenta pit is also filled and we dont have budget for new placenta pit.  Back       7 Codings of  "PPE set supply "  from 7 files.       KII 1 [3818:3924]     use of masks, boots and gloves during waste collection should be conducted for occupational health safety.  Back      KII 2 [8459:8539]     By wearing mask, gloves, grown, boot by the nurse who are working in emergency.   Back      KII 3 [5794:5939]      Personal protective equipment such as gloves, shoes etc are provided to the waste handlers. Health workers take care of themselves by using PPE.  Back      KII 5 [3210:3278]     We cannot afford any of the measures for occupational health safety.  Back      KII 6 [4348:4418]     So they are provided with universal precautions such as masks, gloves.  Back      KII 7 [2785:2831]     We provide them with boots, glovers and masks.  Back      KII 8 [4101:4188]     Sweepers are on high risk from the waste management thus we provide PPEs, masks gloves   Back       2 Codings of  "Periodic waste collection"  from 2 files.       KII 1 [5687:5730]     collect the waste at least 3-4 times a week  Back      KII 4 [1591:1646]     municipality picks up the wastes at least once a week.   Back       5 Codings of  "Pharmaceutical waste"  from 4 files.       KII 10 [ 838: 845]     expired  Back      KII 2 [1947:1966]     medicine can expire  Back      KII 6 [ 368: 388]     various drugs we use  Back      KII 8 [ 580: 613]     pharmacy waste are non infectious  Back      KII 8 [ 677: 695]     expired medicines   Back       2 Codings of  "Placenta Pit"  from 1 file.       KII 2 [3850:3940]     Till now our placenta’s placenta pit is not created. The new one has these kinds of issue.  Back      KII 2 [5423:5496]     Placenta pit also in a process of producing, like it is not produced yet,  Back       22 Codings of  "Policy "  from 10 files.       KII 1 [ 5496: 5571]     National level training for the sweepers should be included in annual plan.  Back      KII 1 [ 5086: 5176]     Infact, we have sent one of our workers in the training but she got transferred elsewhere.  Back      KII 1 [ 5336: 5419]     We expect planning of training from the provincial and central government annually.  Back      KII 10 [ 2386: 2520]     Persons doing monitoring might have confused and less knowledge regarding monitoring, lack of understanding due to temporary placement  Back      KII 10 [ 2522: 2583]     Most of them are not permanent and posted for only one year.   Back      KII 10 [ 9331: 9492]     waste handlers training can be schedule and conducted by government of Nepal. Protocol can be developed and managed. We should also initiate some steps for this.  Back      KII 10 [10481:10578]     Sweeper should also be permanent employee aand should be known about protocol of waste management  Back      KII 2 [10371:10557]     They should be rewarded if they do good work, we can also do monthly meeting to reward them and if someone don't know how to work then they should give proper training or supervise them.  Back      KII 3 [ 1995: 2163]     And how the Municipality is disposing, what effects are happened to environment, we couldn't check. If these activities were also under our control, it would be better.  Back      KII 4 [ 1052: 1111]      government has given less concern towards waste management  Back      KII 4 [ 6511: 6545]     It will be very good if it happens  Back      KII 5 [ 2834: 2924]     We don’t have any guideline for the waste management nor we follow the national standards.  Back      KII 5 [ 4829: 4956]     It is very important to schedule trainings to others diseases for all the workers from medical superintendent to waste workers.  Back      KII 5 [ 5760: 5846]     Central government should atleast provide training to all the workers of the hospital.  Back      KII 5 [ 6068: 6158]     The local government transfers the fund of for the health care management to other sectors  Back      KII 5 [  830:  875]     There is no proper waste management strategy.  Back      KII 5 [ 1020: 1083]     We aren’t allowed to burn the waste which we used to do before.  Back      KII 6 [ 1358: 1375]     specific planning  Back      KII 6 [ 2169: 2286]     There are two things: policy making and implementation. If policy cannot be implemented properly it creates problems.  Back      KII 7 [ 3326: 3404]     Yes we can manage trainings but proper timing and manual should be published .  Back      KII 8 [ 5267: 5392]     Central government should help in implementing guidelines for waste management and help in the monitoring of waste management  Back      KII 9 [10619:10710]     Major issues are we need to have a good policy, and furthermore we need to have a resource.  Back       1 Coding of  "Provision of salary"  from 1 file.       KII 3 [9931:10066]     Government should provide monthly salary to staffs in time. in my opinion, proper policy will bring a good system in waste management.   Back       3 Codings of  "Purchasing Mechanism "  from 3 files.       KII 10 [2230:2262]     procurement is also done at time  Back      KII 6 [2372:2537]     In governmental hospital processing is very slow. Budget and procurement, ill mentality towards waste management are also problems. Also there are problem in tender.  Back      KII 7 [2043:2129]     Also, store keeper, management are also responsible for providing equiipments in time.  Back       2 Codings of  "Salary"  from 2 files.       KII 6 [2796:2901]     But government must understand that the salary is very low which demotivates staffs in managing waste is.  Back      KII 9 [6043:6289]     For example, we are doing work in pandemic, if someone work in the time of corona pandemic, government should provide allowances and 75% to 50% extra salary is provided in different places. These kinds of things should be done by our government.   Back       12 Codings of  "Segregation"  from 6 files.       KII 1 [  507:  552]      different colors coding for different wastes  Back      KII 1 [  897: 1072]     First issues are the segregation of the waste. Patient parties mix up different nature of the healthcare waste together like mixing needles, cottons and blood cottons together  Back      KII 10 [ 4678: 4783]     These who are non-health person have little bit difficulty during waste segregation for waste management.  Back      KII 10 [ 5401: 5628]     Nick simon monitored and evaluated the waste management process. It is one of the part of waste management along with we also monitor and evaluate. As we have a general command waste management, activities going as per command.  Back      KII 2 [ 5685: 5788]     Water bottles which were taken by patient we have separating bucket for that but it is harder to manage  Back      KII 2 [ 2744: 2903]     At first what kind of waste, we separate from solid waste management, we provide that. And another one is their status, if we get some scent then they can mix.  Back      KII 2 [ 3114: 3353]     We are keeping bucket of different color, patient is not keeping those, health workers and train manpower are not able to use them properly. That issue was arising once. Patient used to collect those buckets in one, this is the first issue  Back      KII 3 [ 1144: 1316]      We face so many difficulties to manage the waste. For instance, people unknowingly put any type of waste in the containers placed ate different site for different purpose.  Back      KII 3 [ 4438: 4580]     As I already said there are separate dustbins for waste segregation. But due to lack of awareness people mix the wastes in different dustbins.  Back      KII 8 [ 2506: 2676]     If the people help in segregation of the waste from the source, it would be lot more easier for waste management. But we do lack awareness in local level in this subject.  Back      KII 9 [10367:10492]      By making the budget they need to launch an awareness program in local level, after training they might help in segregation.  Back      KII 9 [ 2638: 2739]     We have done how much we can but till when we have our segregation kit, we cannot manage those issue.  Back       3 Codings of  "Social Security"  from 2 files.       KII 6 [4731:4834]     Government should occupational health safety of the staffs and take responsibility if they get injured.  Back      KII 9 [5050:5190]     Till now we haven’t seen these kinds of issues, but if we had these kinds of issues, we will go for a treatment which was from Hospital side  Back      KII 9 [5286:5487]     If someone suffered with HIV then, what do you think about the medicine if you can get those from the hospital itself.  that would be good if we could get that but till now this service are not served.  Back       1 Coding of  "Staff Transfer"  from 1 file.       KII 5 [5223:5416]     Only one nurse get training in a year. And when she get transferred, no one in the hospital has any idea about the campaign. Thus, every staff should equally get training and awareness program.  Back       1 Coding of  "Train manpower"  from 1 file.       KII 7 [1159:1264]     FIrst is the timely training that must be given to the workers and sweepers to guarantee skillful workers  Back       5 Codings of  "Transportation "  from 5 files.       KII 10 [ 3558: 3817]     we have enough trolley for transportation. There is a dumping site (back side) of hospital. Waste are not transported through patient way as there is issues regarding this, we keep waste separate like health waste are kept separately and other waste are kept.  Back      KII 3 [ 3433: 3571]     Wastes are collected in one place and are collected by municipality but sometimes municipality delays in collecting the waste for 2 weeks.  Back      KII 6 [ 1318: 1332]     transportation  Back      KII 8 [  851:  866]     transportation   Back      KII 9 [10130:10245]     We need to buy auto lay and for that we need a big space and we can do segregation and even we can allocate people.  Back       5 Codings of  "Vaccine"  from 4 files.       KII 1 [4170:4225]     We provide immunization for Hepatitis B to the workers.  Back      KII 1 [4279:4350]     We do provide TT vaccines for the patients, ART for prophylaxis and HIV  Back      KII 7 [2839:2883]     we provide timely vaccination to the staffs.  Back      KII 8 [4197:4300]     provide guidelines for treatment incase of any sort of exposure and T T injections are timely provided.  Back      KII 9 [5213:5284]     they should establish the Vaccination program, TT, Hepatitis B Vaccine.  Back       12 Codings of  "disposal"  from 8 files.       KII 1 [ 655: 808]     All the waste is disposed only after passing them through the autoclave while the needles are subjected to cut off into different pieces before disposal.  Back      KII 10 [1544:1728]     But due to same  misunderstanding or unable to maintain segregation protocol, waste management not done properly and this is being one of the arising issues in hospital waste disposal.  Back      KII 2 [3431:3518]     Yeah, in disposal. If we segregate but at time of disposal it was dumping in one place.  Back      KII 3 [1857:1993]     which is further carried by Municipality vehicle and disposal of that waste also done by them. All the work is done by government itself  Back      KII 3 [2262:2308]     At some places, the waste is put into the pits  Back      KII 3 [1564:1621]     They carry the waste and dumped at specific dumping site.  Back      KII 3 [5177:5258]     The problem is the accumulation of wastes outside the hospital for the long time.  Back      KII 5 [1210:1276]     We do collect waste separately but are disposed all together only.  Back      KII 6 [ 964: 984]     And proper disposal,  Back      KII 6 [6962:7121]     Government should sensitize staffs and general public through trainings, awareness programs, lectures, and demonstrations. Proper disposal site should be made.  Back      KII 8 [ 870: 882]     disposal sit  Back      KII 9 [1426:1446]      issue with disposal  Back       17 Codings of  "facilitation for training "  from 8 files.       KII 1 [ 3990: 4099]     These waste management programs is conducted by low income workers thus awareness program is highly required.  Back      KII 1 [ 5182: 5238]      it important to conduct training for the waste handlers  Back      KII 10 [ 8990: 9171]     This training programme is very important in cleaning staffs. To ensure participation of every staff, training program should be conduct frequently (2-3 participants at one session)  Back      KII 2 [10824:10950]     We can also do it by gathering everyone in the hospital. These waste handlers training should be given to the sweepers anyhow.  Back      KII 2 [11024:11080]     national training center provide training once in a year  Back      KII 2 [11102:11170]     we can give training to the consultant medical officer time to time.  Back      KII 2 [ 8943: 9088]     First, we should educate our staff that what kinds of disease we can get through different waste? We have not talk about training and instrument.  Back      KII 2 [11652:11730]     Waste handlers, all staff members and specially sweepers should give training.  Back      KII 3 [ 6544: 6736]     Awareness programs help secure occupational health. The more frequently awareness program is organized, the more the staffs are secured. More the awareness, staffs practice more safe measures.  Back      KII 3 [ 7845: 8086]     If the training is held in the hospital, all staff can participate. But if it happens outside hospital, committee will decide a person to participate the program. He will make report in 10 days and give feedback and training to other staffs.  Back      KII 5 [ 3532: 3600]     But the main issue we need is proper training and awareness program.  Back      KII 6 [ 3513: 3785]     Training on using different colored buckets for collecting different type of wastes can be given to sweepers. I think municipality should provide such trainings to aware sweepers to understand and  use different buckets for plastics, infected urine, degradable wastes etc.  Back      KII 6 [ 4243: 4347]     They are exposed to waste containing various contagious diseases such hepatitis B, hepatitis C, HIV etc.  Back      KII 6 [ 5602: 5702]     To keep hospital clean and healthy, all the staffs should be trained how to dispose waste properly.   Back      KII 7 [ 3130: 3251]     timely training and awareness for waste handlers is very important which ultimately helps in implementing the guidelines.  Back      KII 9 [ 4195: 4341]     We are giving training time to time and UNICEF also provide training related to segregation, waste management but it is really difficult to manage  Back      KII 9 [ 7892: 8351]     Till now we are getting training by UNICEF, their training is good and effective also Employee   who are working on waste management are down level people. that’s why they have an important role because they are active. By doing work if they are lack of education then we should help that how to handle everything. if we don’t let them learn then they might not work, they might face difficulties and they might have lung cancer that’s why they need training.  Back       11 Codings of  "hazardious waste"  from 8 files.       KII 1 [277:297]     needles and syringes  Back      KII 10 [847:899]     chemicals use during preparing the slide in the lab,  Back      KII 2 [980:987]     needles  Back      KII 3 [896:922]     waste containing dangerous  Back      KII 3 [927:977]     infectious contents are similar to those, isn't it  Back      KII 4 [753:776]     noodles for the patient  Back      KII 5 [261:281]     needles and syringes  Back      KII 6 [214:259]     sharp instruments are generated from hospital  Back      KII 6 [636:673]     Sharp instruments are also hazardous.  Back      KII 6 [759:776]     sharp instruments  Back      KII 9 [247:255]      needles  Back       25 Codings of  "infectious waste"  from 9 files.       KII 1 [ 454: 482]     containing blood and tissues  Back      KII 10 [ 212: 224]     cotton swab,  Back      KII 10 [ 590: 698]     saliva, blood, other liquid solution which is discharge from patient body during procedure and cotton swabs.  Back      KII 10 [ 900: 938]     sputum and specimen container, blood ,  Back      KII 2 [ 779: 787]     placenta  Back      KII 2 [ 851: 870]     pus containing swab  Back      KII 2 [ 872: 898]     materials used in dressing  Back      KII 2 [ 917: 937]     waste generate in OT  Back      KII 2 [ 989:1007]     infected materials  Back      KII 2 [1305:1311]     sputum  Back      KII 2 [3686:3698]     spectrum T.B  Back      KII 3 [1030:1058]     Some are pus containing swab  Back      KII 3 [1060:1068]     OT blood  Back      KII 4 [ 473: 584]     there is possibility of transmission of various diseases through these wastes. Syringes are disposed separately  Back      KII 4 [ 753: 776]     noodles for the patient  Back      KII 4 [ 862: 900]     the wastes which can transmit diseases  Back      KII 5 [  92: 204]     We have started operation procedure in the hospital thus blood mixed waste are one of the major waste generated.  Back      KII 5 [ 253: 259]     gloves  Back      KII 5 [ 625: 640]      infectious pus  Back      KII 6 [ 188: 212]     placenta during delivery  Back      KII 6 [ 390: 397]     tissues  Back      KII 6 [ 570: 634]     Infected urine, sputum, and placenta are the source of infection  Back      KII 7 [ 292: 329]     biological materials, placenta and OT  Back      KII 9 [ 257: 275]     blood mixed cotton  Back      KII 9 [ 527: 536]      placenta  Back       4 Codings of  "insurance"  from 3 files.       KII 10 [7161:7352]     Government should manage facility of health insurance to them. Employees are on the basis of daily wages, if health insurance facility is provided then, they would be motivated towards work.   Back      KII 5 [3279:3393]     But we do expect our government to allow insurance to the workers as well as free checkup to the infected workers.  Back      KII 6 [4419:4598]     If anything happens to them, faculties should be managed. System of using various buckets for collecting sharp wastes, gloves, plastics should be implemented to minimize injuries.  Back      KII 6 [5053:5174]     Yes, hospital can do these. But, I mean government should also support in these conditions and provide medical insurance.  Back       10 Codings of  "knowledge"  from 7 files.       KII 10 [1344: 1415]      But the personal works in waste disposal, they do not know about this.  Back      KII 2 [6067: 6139]     Another thing is we have lack of education training on waste management.  Back      KII 2 [9999:10263]     We talk about training and awareness. what is needed for sweeper regrading training related to health and awareness? They know about the waste, how to manage different kinds of waste, how to work by protecting ourself by the waste. And for that they need training.  Back      KII 4 [4770: 4873]      participants are able to understand how the waste can be managed and proper way of disposal of wastes.  Back      KII 5 [3816: 3952]     We do have guidelines but cannot implement anything. Thus, everyone should get knowledge regarding waste management and the proceedings.  Back      KII 5 [4402: 4521]     It is very important but we don’t have adequate knowledge and awareness. We do have documents but never implement that.  Back      KII 6 [6251: 6432]      Trainings and awareness provide knowledge of dealing with wastes and segregating at site to staffs directly dealing with waste such as sweepers, lab technicians and health workers.  Back      KII 8 [4662: 4856]      I guess training is required for all sectors. It would be great if we could provide training to all the staffs , sweepers management teams sothat they can learn more about the waste management.  Back      KII 9 [2164: 2237]     Sick people do not have a proper knowledge to throw which waste to where.  Back      KII 9 [4602: 4802]     Firstly, we are giving them a knowledge about hazardous waste, also we told them about the problems. They are taking universal precaution while they work, we followed them, also we are accepting them.  Back       1 Coding of  "laboratory waste"  from 1 file.       KII 2 [3738:3850]     These kinds of issues are facing by us. Chemical stool or blood which are disposed by lab are harder to manage.   Back       1 Coding of  "lack of supervision"  from 1 file.       KII 2 [5827:5861]     we need monitoring and evaluation   Back       20 Codings of  "manpower"  from 9 files.       KII 1 [ 2419: 2444]     major problem is manpower  Back      KII 1 [ 5642: 5682]     Local government should provide manpower  Back      KII 1 [ 1080: 1188]     another major issue is the lack of proper manpower. Hospital doesn’t have efficient number of waste handlers  Back      KII 1 [ 3225: 3387]     We do not have proper manpower so that we can allocated focal person. Our anesthesia is looking for store and administration. This is the reality of the hospital.  Back      KII 10 [10227:10479]     I think a hospital should appoint permanent person who will be responsible for waste management if these is temporary employee then, we have to explain provide councelling regarding protocol to them time and gain only the waste will be managed properly  Back      KII 10 [10626:10738]     I and other staffs to instruct them for this. Hospital should appointee healthcare manager for effectiveness.     Back      KII 2 [ 5136: 5251]     We need manpower and autoclave also. while disposing gloves and needle you have plastics while autoclave, am I righ  Back      KII 2 [ 6022: 6065]     we also have problem with the manpower also  Back      KII 3 [ 1317: 1397]     Even more, we have not sufficient manpower; just one or two persons are deputed.  Back      KII 3 [ 2164: 2203]     Now due to problem of manpower shortage  Back      KII 3 [ 2963: 3062]     And for implementation of guidelines for performing the work as required, no manpower is available.  Back      KII 3 [ 3911: 4015]     The problem is not the lack of buckets but lack of manpower who can utilize these resources efficiently.  Back      KII 3 [ 4791: 4810]     inadequate manpower  Back      KII 4 [ 1272: 1315]     there is lack of sufficient waste handlers.  Back      KII 4 [ 1528: 1559]     not providing sufficient staffs  Back      KII 5 [ 2308: 2316]     manpower  Back      KII 7 [  811:  908]     Another problem is related to lack of proper manpower. We don’t have adequate number of sweepers.  Back      KII 8 [ 2773: 2911]     First of all the issues is of the manpower. We only have 3 sweepers who work on shift basis thus segregation of the waste is not possible.  Back      KII 9 [ 9699: 9837]     we need a good manpower. Those people who worked actively for department in any shift that segregation, this is what I am seeing right now  Back      KII 9 [10711:10768]     We need more employee for motivation program and training  Back       2 Codings of  "member for annual planing "  from 2 files.       KII 10 [7648:7697]     there are manager, me (MS) and nursing in-charge.  Back      KII 4 [5278:5426]     If so we will include emergency incharge, heads of all departments, indoor incharge, medical recorder, lab incharge, administration and finance head  Back       9 Codings of  "planing implimentation "  from 6 files.       KII 1 [1669:1794]     We have made a healthcare waste management plan but due to inadequate budget and manpower, implementation is a big challenge.  Back      KII 1 [4422:4533]     We plan the annual healthcare waste management strategy according to the budget allocated for waste management.  Back      KII 10 [2783:2966]     there is a hospital strengthening program in hospital under which we plan for waste management activities. One nurse in-charge is head and under her waste management planning is done.  Back      KII 3 [6992:7059]     Overall it can be said that decision making processes is happening.  Back      KII 3 [2705:2841]     No, none planning is not happen, planning is done. Now days in every organization, cleanliness is must which are immediate seen by eyes.  Back      KII 5 [1879:1922]      Also  the control of the pandemic is also   Back      KII 7 [2998:3055]     We don’t have any annual plan in waste management sector.  Back      KII 9 [6854:6932]     While we make yearly planning according to the government norms, we have 15%.   Back      KII 9 [7278:7517]     we haven’t done meeting, haven’t planning yearly planning, we discussed in committee meeting, in the committee meeting we have medical officer and members from another department and we discussed in the meeting related to waste management.  Back       3 Codings of  "post exposure service"  from 2 files.       KII 3 [6129:6455]     There is emergency service for the accidental and post exposure to wastes. There are separate departs for cure of exposure to infectious bloods such as HIV. For the hospital staffs, treatment is done without any formality. If such issues occurred in hospital during duty time, hospital provides even higher treatment facility.  Back      KII 9 [5492:5681]     There is nayarani hospital, you can contact them if you need medicine. we have a good relation with them. They are for safety if needed for medicine they would contact and ask for medicine.  Back      KII 9 [5683:5842]     Yes, medicine is available there, You may not get every medicine but there are HIV medicine available and for Hepatitis B medicine you have to visit Kathmandu.  Back       2 Codings of  "recording "  from 2 files.       KII 1 [3629:3712]     we do not have any checklist and log books for the waste generated by the hospital.  Back      KII 7 [1266:1394]     Other is effective working manpowers and checklist of work division is also hindering effectiveness of guideline implementation.  Back       9 Codings of  "solid waste "  from 3 files.       KII 2 [1313:1318]     stool  Back      KII 2 [2191:2204]     water bottles  Back      KII 2 [2206:2214]     plastics  Back      KII 4 [ 149: 153]     pads  Back      KII 4 [ 197: 219]      cotton bandage, gauge  Back      KII 4 [ 389: 393]     pads  Back      KII 4 [ 395: 409]     gauge bandages  Back      KII 4 [ 411: 419]     syringes  Back      KII 5 [ 418: 431]     Gauge pieces,  Back       2 Codings of  "staff hiring "  from 2 files.       KII 1 [1321:1389]     HR department which is failing to appoint workers in contract basis.  Back      KII 3 [9657:9759]     And I would recommend central and province government to recruit manpower for proper waste management.  Back       9 Codings of  "stakeholder"  from 5 files.       KII 1 [1873:2041]     Basically, ward in-charges are mainly related to management of the issues related to healthcare waste management. Also, sweepers are also related directly to the issue.  Back      KII 1 [2106:2317]     The role of community is negligible given the amount of support they can give. In case of municipality, some hospitals are helped by them by allocating budgets, providing vehicles for waste collection and so on.  Back      KII 1 [2818:3070]     We do organize meetings but waste management is a big challenge in district hospital. We do hear news about condition of government hospital. But the people and society should be aware for keeping the hospital clean and also helping in waste management  Back      KII 2 [6266:6380]     It would be easy If the municipality has given budget on time. You mean one of the problems is municipality? Yes!!  Back      KII 2 [9350:9470]     Yes!! While planning for the yearly plan we need to choose community team from where we need to choose one focal person.  Back      KII 2 [9471:9670]     We need to plan how to manage waste and we should give work to all emergency, delivery room and ask for feedback and follow up. After observing and follow up we can know that how that works was done.  Back      KII 5 [1342:1576]     Medical superintendent is the primary person responsible with waste management plan. Besides, staffs of waste management, hospital management team, ward staffs, nurses emergency staffs, paramedics, sweeper are also equally responsible  Back      KII 8 [2084:2202]     Medical superintendent, sweepers and waste management committee are the main stakeholders related to waste management.  Back      KII 9 [4343:4437]     As a chairperson we monitor these things but I personally think it is not effective till date.  Back       6 Codings of  "sterilization "  from 4 files.       KII 2 [5157:5206]     autoclave also. while disposing gloves and needle  Back      KII 2 [5386:5422]     We haven't waste by doing autoclave.  Back      KII 5 [1168:1209]     We dont have any autoclave for the waste.  Back      KII 6 [7122:7153]     Autoclaving should be improved.  Back      KII 9 [ 963:1083]     Yes, we are doing segregation but other things like blood mix and they need to autoclave, we are not able to send them.   Back      KII 9 [3813:4022]     now it needs to autoclave, we needs to disinfect then it can help, we also need setup for that and our province government municipality are working for it, proper setup might help otherwise it is not possible.  Back       6 Codings of  "storage"  from 4 files.       KII 2 [3358:3430]     another issue is because of the management they are mixing in one place.  Back      KII 3 [1814:1856]      storage we collect the waste at one place  Back      KII 3 [1716:1758]     Well sir, what are the storage facilities.  Back      KII 4 [1115:1154]      inadequate space for disposal of waste  Back      KII 4 [1156:1227]     It is difficult to manage healthcare wastes due to insufficient spaces;  Back      KII 7 [ 909:1026]     Also, segregation of waste, lack of awareness of disposal of waste and other waste disposal plant is also issue here.  Back       1 Coding of  "use of color bucket "  from 1 file.       KII 2 [7751:7784]     using bucket with different color  Back   
