# Supplementary material for: Knowledge about handling hazardous materials as factors associated with adherence to healthcare waste management practices among waste handlers at government district hospitals of Madhesh province, Nepal: A quantitative-qualitative methods study
Source: PLOS Glob Public Health. 2024 Dec 5;4(12):e0002028. doi: 10.1371/journal.pgph.0002028 (PMC11620432; doi:10.1371/journal.pgph.0002028)
Supplement: S1 File — (DOCX) [file pgph.0002028.s003.docx]

**KII 1**

**I. What are the major types of hospital waste generated by the hospital?**

R: District hospital generates healthcare wastes like Gauge Bandages, cottons and bandages from operation procedures. Besides this, papers, food wastes plastic bottles, needles and syringes and also different chemical liquids are generated.

**I: How do you classify waste as general hospital waste and hazardous hospital waste?**

R: Healthcare waste containing blood and tissues are separated. There are different colors coding for different wastes generated. Also, dustbins are categorized according to the nature of the waste as green, yellow, red. All the waste is disposed only after passing them through the autoclave while the needles are subjected to cut off into different pieces before disposal.

**I : What are the issues related to healthcare waste management in district hospital?**

R: First issues are the segregation of the waste. Patient parties mix up different nature of the healthcare waste together like mixing needles, cottons and blood cottons together. Also, another major issue is the lack of proper manpower. Hospital doesn’t have efficient number of waste handlers also management of the available manpower’s is difficult due to politics among the workers. Also the problem is also related to the HR department which is failing to appoint workers in contract basis. Another big issue is lack of budget. Fortunately, this year the budget is allocated for healthcare waste management also, thus we expect biomedical engineers working in healthcare waste management soon.

**I: Is there any problems related to specific planning’s in the hospital?**

R: We have made a healthcare waste management plan but due to inadequate budget and manpower, implementation is a big challenge.

**I: Who are the responsible persons related to healthcare waste management?**

R: Basically, ward in-charges are mainly related to management of the issues related to healthcare waste management. Also, sweepers are also related directly to the issue.

**I: What are the roles of community members and municipality?**

R: The role of community is negligible given the amount of support they can give. In case of municipality, some hospitals are helped by them by allocating budgets, providing vehicles for waste collection and so on.

**I: What are the problems related in implementation of healthcare waste management guidelines?**

R: The major problem is manpower and changing their mindset towards the guidelines.

**I: How do you monitor healthcare waste management?**

R: The healthcare waste management is inspected by hospital superintendent, administration and waste handlers in charge in every one- or two-week’s interval.

**I: Do you have an administrative flow chart deciding the roles of superintendent, in charges in the hospital?**

R: We do organize meetings but waste management is a big challenge in district hospital. We do hear news about condition of government hospital. But the people and society should be aware for keeping the hospital clean and also helping in waste management.

**I: Have you allocated someone as the focal person to monitor the healthcare waste management in the hospital?**

R: We do have budget issues in this case. We do not have proper manpower so that we can allocated focal person. Our anesthesia is looking for store and administration. This is the reality of the hospital.

**I: There are different monitoring plans like arranging checklist on marking which department conducted waste management and also preparing log book noting the total waste generated. Does district hospital apply any of the procedure?**

R: No, we do not have any checklist and log books for the waste generated by the hospital.

**I: How do you ensure the occupational health safety of the workers in the hospital?**

R: Regular checkups, use of masks, boots and gloves during waste collection should be conducted for occupational health safety.

**I: Do you organize trainings and awareness campaigns as well?**

R: These waste management programs is conducted by low income workers thus awareness program is highly required.

**I: Do you provide post exposure services for the injured workers?**

R: We provide immunization for Hepatitis B to the workers.

**I: Do you have vaccination programme in hospital?**

R: We do provide TT vaccines for the patients, ART for prophylaxis and HIV.

**I: How do you conduct annual plan for healthcare waste management?**

R: We plan the annual healthcare waste management strategy according to the budget allocated for waste management.

**I: Do you plan healthcare waste management strategies beforehand?**

R: No, plans are made only after budget allocation.

**I: Who are the persons responsible for preparing annual plans?**

R: Medical superintendent, administration and in charges sit together and plan the annual plans accordingly.

**I: Do you involve healthcare waste handlers and house keepings during planning’s?**

R: We do include them during planning.

**I: How much do you think is it necessary to conduct training related to health and safety to the healthcare waste?**

R: It is very important. Infact, we have sent one of our workers in the training but she got transferred elsewhere.

**I: Is it important to conduct training for the waste handlers?**

R: Yes, it is very important.

**I: How can you manage the trainings that are required to them?**

R: We expect planning of training from the provincial and central government annually.

**I: How can provincial and central government plan the training programs?**

R: National level training for the sweepers should be included in annual plan.

**I: How do you think waste management can be conducted efficiently?**

R: Local government should provide manpower and collect the waste at least 3-4 times a week and also allocate budgets to the hospital.

Provincial government and central government should provide training and budgets for human resources.

**KII 2**

Ok Let's Start

Hello Doctor

I am from patan health science bureau, student of

MPH. And I am preparing my thesis on the topic of health

management. My title is “Among waste handlers at province no.2. For

that I am now concerned with KII. And I hope you will help me for

carrot. Doctor, could you please introduce yourself?

Me from bardibas hospital working as a medical

officer since last 2 years. Before that I worked at jaleshwor for 2 years.

Now I am working here.

You are focal person here. right?

Yes, yes yes

Let's get started

JNP: What kinds of are produced in district and district level Hospitals?

DRP: Now, there are some solid healthcare waste produce in hospital. These are generate in delivery room i.e. placenta. Certain healthcare waste is also generated in emergency room, pus containing swab, materials used in dressing. Other healthcare waste generate in OT. And some are also called hazardous like needles, infected materials means blood wound while washing waste.

Yes! And what is about liquid waste? How much liquid waste produced in that hospital?

Liquid healthcare waste also generate in hospital. When some things related with body and health instruments clean at laboratory. i.e. lab water, urine for investigation, sputum, stool.

Yes, from lab waste urine, sputum, and stool. These are one category, which is also called solid waste and Liquid waste. And another category is like you said Hazardous, infectious, non-infectious.

JNP: what, we can understand by general health related garbage which are

produced in the hospital?

General what?, What we can understand by general health related garbage?

DRP: Hospital may have some kinds of waste like Urine, stool, pathological

waste, waste which can come from different labs in hospital or the

kind of waste which come from emergency or dressing room. Another

kind of waste is pharmaceutical waste. Like medicine can expire or we

have to manage in short index or chemicals which are used in labs. You

we can take example of our lab. As a whole like you just said this

garbage is hazardous and infectious right?

And the water boiled by sick people, water bottles, plastics.

JNP: And what about papers?

DRP: Yeah, they are also a kind of waste in the hospital.

JNP: Ok I want to go further now.

What are the waste management issues that are connected to

organization issue, what is happening there?

Would you like to add something in this topic?

What kind of issue sir?

Like hospital produce waste and these waste needs to manage. What

kinds issues you are facing while managing these you may have facing

transportation issue, site issue, lack of planning issue, do you have any

other issues?

DRP: At first what kind of waste, we separate from solid waste

management, we provide that. And another one is their status, if we

get some scent then they can mix.

JNP: What are the management to control these in your hospital?

How you are seeing these?

DRP: Situation is we just shifted to the new and the issue is still existing and

we are trying to solve those issues.

We are keeping bucket of different color, patient is not keeping those,

health workers and train manpower are not able to use them properly.

That issue was arising once. Patient used to collect those buckets in

one, this is the first issue and another issue is because of the

management they are mixing in one place. Yeah, in disposal.

If we segregate but at time of disposal it was dumping in one place. It

was segregate and not used. Expect taking them to municipality they

are dumping in same place. And another thing is for doing waste

management, how to manage them, spectrum T.B which come to lab.

How to manage them? These kinds of issues are facing by us.

Chemical stool or blood which are disposed by lab are harder to

manage. Till now our placenta’s placenta pit is not created. The new

one has these kinds of issue.

JNP: Have you seen waste guideline of 2014?

Yes!!

These kinds of guideline are too many by the Nepal government, the

2014 was the latest guideline.

But we have another guideline called “Hand Pustika” which was

published in the year 2020. But maybe they are not circulating in

district.

It was recently published, 2 or 3 months. I think 3 or 4 months ago,

how it was implemented? Was that any kinds of guideline issue or

not?

DRP: It is a big issue like according to the new guideline expect doing

incinerate collecting water bottles are saying in one one process.

According to the latest guideline there is a process of making needle

by cement.

JNP: Yes! In this priority what is given to the priority. Do you have any issue

related to this?

Pardon please?

JNP: Health care is the most priority thing in the hospital. What is your first

priority? You have priority on Emergency services or how to improve

emergency services?

JNP: Likewise, for Labour room we have priority to good delivery. Likewise,

how much your hospital has priority for Health care waste?

Health care is the most prior topic, our hospital was new which was

shifted to the new place that's why we are planning for that. We need

manpower and autoclave also. while disposing gloves and needle you

have plastics while autoclave, am I right?

Hmm…. Yeah, this is also a priority for us. We are slowly working on it.

Now it is our first priority, how is the situation there? We haven't

waste by doing autoclave. Placenta pit also in a process of producing,

like it is not produced yet, Remaining things are like we are keeping

needles in a pit box. We are keeping plastics materials, sharp items,

gauge piece like cleaned wound which are using daily are keeping

separately. Water bottles which were taken by patient we have

separating bucket for that but it is harder to manage. As I am adding

organizational issue, we need monitoring and evaluation while

observing by the organization. We also need extra budget for that,

Hospital does not have its own budget which is in municipality. Budget

has one problem; we also have problem with the manpower also.

Another thing is we have lack of education training on waste

management.

JNP: Who is the responsible person for lack of manpower, lack of training?

and the current issue faced by the hospital?

DRP:It would be easy If the municipality has given budget on time.

You mean one of the problems is municipality?

Yes!!

It was the problem before but now it is process and maybe we are in

a process to receive some fund. That means not the responsible

person is municipality?

There is a community level person, also a person from our

administration, he manages the administration part.

Ok let's move toward to another question.

There is a guideline on waste which was made on 2014, there are

some important problems which might cause due to implementing

the guideline.

That was implemented but the fund which needs to be provided to

the District and regional hospital was going for laundering.

Yes!! By following this guideline what are the things which caused this kind of

issues?

According to the guidelines it is said that for waste management we

will get manpower, budget, training. Also, how to manage different

kinds of waste. I think there are 3 or 4 problems.

How to identify the issue?

By requesting the budget from municipality, by providing training to

all staff and giving responsibility to them, by implementing the law and

supervise them, by doing monthly and weekly meeting about the

progress of their work. I think after following these we are able to

identify and fix the issues.

You mean to say it will be implemented after waste management

guideline?

Yes sir!!

JNP: Ok let's move towards another topic.

How you monitor the healthcare waste management?

For that we are using bucket with different color, By Observing Sharp

tools, disposable, and non-disposable waste were dump to

pathological waste department.

How chemical was managed?

how emergency waste, dressing, placenta in delivery is out to the

different department?

DRP: That can be known by doing meeting. Who is the person to know this

kind of follow up? Who are doing following about these issues?

There is different person from different department. Like nursing,

emergency, medical officer, and administration.

JNP: Shall we observe by making the checklist or not?

DRP: Yes!

JNP: Can we make log book also?

DRP: Yes yes!!

JNP: Ok let's move to another topic.

How employees can secure their occupational health?

By wearing mask, gloves, grown, boot by the nurse who are working

in emergency. It is the pandemic time so they need to wear gloves.

Specially how to secure waste handlers?

Pardon Please?

DRP: Waste handlers should wear mask, gloves, grown. Yeah, they have to

wear that. What about equipment, PPE, Sputum, also we need to

disinfect by putting them to the tank. Now for T.T we can vaccinate

needle.

JNP: What is your role in awareness training program? Did it affect

anything?

DRP: First, we should educate our staff that what kinds of disease we can

get through different waste?

We have not talk about training and instrument.

JNP: Okay let's talk about another topic.

As I know about you, you were working as a medical officer since last

4 year.

Yes!! 2 years in government and 2 year for loksewa. What I want to know is

what we can do for the health care waste? Yearly planning?

DRP: Yes!! While planning for the yearly plan we need to choose community

team from where we need to choose one focal person. We need to

plan how to manage waste and we should give work to all emergency,

delivery room and ask for feedback and follow up.

After observing and follow up we can know that how that works was done.

JNP: How to implement further plan?

We need to bring goods and materials, see if the dustbin was used

different color or not. need to know the status of autoclave, placenta

pit, need to know about the management for those materials.

Likewise, shall we make time table for that?

Yes, Yes, we can do that.

Ok let's move forward.

We talk about training and awareness. what is needed for sweeper

regrading training related to health and awareness?

They know about the waste, how to manage different kinds of waste,

how to work by protecting ourself by the waste. And for that they

need training.

JNP: can we involve employee to the training?

How waste handlers involve in training, what we need to do?

They should be rewarded if they do good work, we can also do

monthly meeting to reward them and if someone don't know how to

work then they should give proper training or supervise them.

JNP: I want to add something in this topic. NTVC provide certain training

related to disease for nursing management. Many of them are proving

training like this. can we provide train sweepers by the gov. training

programme to the national level, local level?

DRP: We can also do it by gathering everyone in the hospital. These waste

handlers training should be given to the sweepers anyhow.

They need to motivate. Yeah, we can do that but I want to say is what

if national training center provide training once in a year?

We can do that sir; we can give training to the consultant medical

officer time to time.

Can we work like this?

Ok we are almost done with the today's interview. So, one last

question sir?

JNP: What you would like have from your Nepal government, local

government, your organization?

DRP: We are getting from province level. They should give training to our

all staff related to waste management, and another one is budget and

follow up about the training, whether the training was effective or

not? If follow up made the government then it will be fully

implemented. Waste handlers, all staff members and specially

sweepers should give training. They need more motivation, I think.

Ok thank you

Have a good day

Okay Thank You

**KII 3**

Namaste! My self-Jot Narayan Patel, student of MPH at Patan Academy of Health Science. At present I am Pursuing this research for Master's Thesis. As my research area is province 2, hence I have to go through KII interview by 10 hospital medical superintendent. So, I very humbly wish to get your co-operation in this regard.

Namaste! I am ----------. At Present Medical Officer, District Hospital.

**JN: Well doctor, what are the different types of healthcare waste in this hospital?**

**BM**: There are many types of healthcare waste in which some are water soluble and others hard and non-water soluble.

**JN: So, what is meant by healthcare waste? We have understood one normal and other infectious.**

**BM**: No, we should not say infectious and non-infectious. Waste can be degradable or non-degradable.

**JN: It means like paper and foods are normal and degradable waste and the waste containing dangerous and infectious contents are similar to those, isn't it?**

**BM**: Yes, you're right. Other are like paper, food. Some are pus containing swab, OT blood,

**JN: Next, what are the normal issues faces by this district hospital?**

**BM**: We face so many difficulties to manage the waste. For instance, people unknowingly put any type of waste in the containers placed ate different site for different purpose. Even more, we have not sufficient manpower; just one or two persons are deputed. If the waste is not graded well, they also feel problem in performing their duties and we collect the waste at one place which is carried by vehicle of Municipality. They carry the waste and dumped at specific dumping site.

**JN: So, for you, disposal of waste is major issue or not? If there is any transport problem?**

**Well sir, what are the storage facilities. Is there proper place for storage in hospital?**

**BM**: For storage we collect the waste at one place which is further carried by Municipality vehicle and disposal of that waste also done by them. All the work is done by government itself.

And how the Municipality is disposing, what effects are happened to environment, we couldn't check. If these activities were also under our control, it would be better. Now due to problem of manpower shortage, shortage of place all activities could not perform well. At some places, the waste is put into the pits. Now onwards we are adopting waste management system by which waste can be process through Autoclave machine and that sterilized waste would be less hazardous.

**JN: Apart from waste management, are there any other issues also? How do we plan and how guidelines are properly implemented? There may be some places where no importance is given to waste management. What are your comments please?**

**BM**: No, none planning is not happen, planning is done. Now days in every organization, cleanliness is must which are immediate seen by eyes. The clean place is an attraction also. If someone sees the littering waste, he/she must say "Why so littering of waste?”

And for implementation of guidelines for performing the work as required, no manpower is available. Waste is littered 24 hours which should be collected and now premises is so large, in which time and how much time is required to check is not possible. All problems are due to shortage of manpower.

**JN: Do you mean there is inadequate manpower for monitoring and evaluation of wastes?**

**BM**: One person cannot monitor and evaluate whole area. So extra manpower is needed. Wastes are collected in one place and are collected by municipality but sometimes municipality delays in collecting the waste for 2 weeks. Financial problem also exist. Sometimes sweepers are at strike and wastes last on the road for about 20 days. It is difficult to find workers as well.

**JN: Is there issues such as problem in buying soaps, buckets and other resources?**

**BM:** We don’t know about the past but nowadays there is separate budget for waste management in hospital. The problem is not the lack of buckets but lack of manpower who can utilize these resources efficiently.

**JN: Who do you think are the responsible person for monitoring, transportation, segregation?**

**BM:** The responsibility for waste management is given to the head of various departments. Store looks for compound, management of indoor waste. There is committee for management of wastes. There are incharges for various areas. So, everyone is responsible.

**JN: How much do committee members and municipality is responsible?**

**BM:** As I already said there are separate dustbins for waste segregation. But due to lack of awareness people mix the wastes in different dustbins. I think this problem will also be solved with time.

**JN: What are the difficulties in implementing healthcare waste management guideline and how will you solve this?**

**BM:** The main problems are lack of space and inadequate manpower. Also, other problem is lack of sufficient finance and budget.

**JN: In your opinion, how can the coordination and monitoring are done in waste management?**

**BM:** Coordination and monitoring is very important. It may be the problem for big hospitals but not for small hospitals with around 50 to 60 staffs. In small hospitals, staffs share the problems and solve it. The problem is the accumulation of wastes outside the hospital for the long time.

**JN: You have experience in monitoring of waste management. How do you evaluate during waste management? Whom do you give responsibilities?**

**BM:** Store submits the report of waste and the report is monitored and evaluated. Otherwise, we conduct a meeting and find out the solution for the problem.

**JN: Have you practiced using checklists and logbooks for each ward whether they are disposing wastes or not?**

**BM:** No, it is not practiced.

**JN: What can be done to secure occupational health especially for healthcare waste management?**

**BM:** Personal protective equipment such as gloves, shoes etc are provided to the waste handlers. Health workers take care of themselves by using PPE. So there not direct exposure to wastes.

**JN: What types of facilities are available for post exposure such as cuts with glass, exposure to infected bloods and what are the preventions?**

**BM:** There is emergency service for the accidental and post exposure to wastes. There are separate departs for cure of exposure to infectious bloods such as HIV. For the hospital staffs, treatment is done without any formality. If such issues occurred in hospital during duty time, hospital provides even higher treatment facility.

**JN: How does awareness programs help secure occupational health safety?**

**BM:** Obviously, Awareness programs help secure occupational health. The more frequently awareness program is organized, the more the staffs are secured. More the awareness, staffs practice more safe measures.

**JN: How do you prepare annual plan for management of specific hospital waste?**

**BM:** We don’t have such annual plans. However there is routine and budget for management of wastes. Hospital itself manages internal resource for waste management and manpower. Overall it can be said that decision making processes is happening.

**JN: Is there any separate annual plan for waste management?**

**BM:** There is routine for waste management but not the annual plan. If there is 5 sweepers and we have to recruit more 5 staffs for waste management, plan may fail. We are opening tender for waste management. After this there will be changes in manpower and we have to plan accordingly. So we have not prepared the annual plan but separated budget for healthcare waste management.

**JN: Why are training and awareness programs are important?**

**BM:** As I already said, more the repetition, more the people remember and make priority. It refreshes the minds of the staffs. If government stops promoting COVID, people will stop washing hands with soap.

**JN: How can you make staffs participate in healthcare waste management?**

**BM:** If the training is held in the hospital, all staff can participate. But if it happens outside hospital, committee will decide a person to participate the program. He will make report in 10 days and give feedback and training to other staffs.

**JN: National training center is organizing various trainings each year. How will it be if they prepare schedule of training on healthcare waste management for waste handlers? Does everyone fit for the training?**

**BM**: It is difficult to define waste handler. Are they street sweeper or hospital waste managing staff, Few staff can be sent to the training. Also a consultant can be hired and organize training in the hospital so that everyone can participate. This will be more effective.

**JN: it is different for moderators and sweepers. As you said it cannot be done in one place. For example in 2/3 sites training can be organized for half from lahan and half from siraha twice so that it won’t affect the work. Is it possible to do so?**

**BM:** Yes, it can be done by organizing 2-3 days program for hospitals. If the training involves all level of staffs such as sweeper, moderators, and other health workers, it will be effective rather than giving training separately.

**JN: It won’t be effective when separate training is given, will it?**

**BM:** Yes, it won’t be effective. One can understand their importance when together.

**JN: How do awareness programs brings decline in production of waste generation?**

**BM:** Due to awareness, people reuse the reusable waste and help reduce waste.

**JN: Recycle and reuse can be done when you have knowledge, can’t it?**

**BM:** obviously.

**JN: What will you recommend government: central, province and local in management of waste?**

**BM:** local government should manage dumping sites, manage health care hazardous waste, and manage hospital waste. And I would recommend central and province government to recruit manpower for proper waste management. And government should give right to hospitals to recruit staffs for waste management. It will be easy for hospitals if there is an effective guideline and extra manpower. Government should provide monthly salary to staffs in time. in my opinion, proper policy will bring a good system in waste management.

**KII 4**

**I: What is the health wastes generated from district hospital?**

R: Waste specially generated from maternity ward such as syringes, pads, and waste generated from emergency such as cotton bandage, gauge and plastics are health waste.

**I: What type of wastes can be considered as general waste and dangerous waste? How can we differentiate?**

R: The waste with blood such as pads, gauge bandages, syringes are dangerous and must be disposed carefully because there is possibility of transmission of various diseases through these wastes. Syringes are disposed separately.

**I: What are the sources of general wastes such as paper, plastics, food wastes etc.?**

R: The main source of general wastes is visitors. They bring foods, biscuits and noodles for the patient and throw plastics.

**I: What are dangerous and infectious health wastes?**

R: They are the wastes which can transmit diseases. We manage these wastes regularly and carefully.

**I: What do you think are the waste management issues for district hospital?**

R: The main issue is that government has given less concern towards waste management and inadequate space for disposal of waste. It is difficult to manage healthcare wastes due to insufficient spaces; however we are managing these wastes. Also, there is lack of sufficient waste handlers.

**I: As you said the issues are lack of manpower and spaces. What are the issues related with organization such as transportation and disposal?**

R: Though municipality is responsible for waste management, they are not providing sufficient staffs. The waste would be managed if municipality picks up the wastes at least once a week.

**I: Though hospitals are managing health care waste, weaknesses can be seen in planning and implementation of guidelines. What is the situation in your hospital?**

R: Weaknesses are everywhere. We have published an advertisement for the post of medical superintendent for healthcare waste management. But no one applied for the post and our advertisement failed.

**I: It seems that monitoring and supervision is not done in healthcare waste management. What do you think?**

R: It is not done anywhere.

**I: Does procurement have affected the healthcare waste management in the hospital?**

R: No, it hasn’t.

**I: Who are responsible for the management of waste management in district hospital?**

R: Mainly the hospital staffs. Many community persons are not needed.

**I: What is the role of municipality in managing hospital wastes?**

R: I don’t know the role of municipality in managing hospital waste.

**I: What can be the problems in implementing the healthcare waste management guideline of Nepal?**

R: It is good if there are not any problems after implementation of the guidelines. In current situation there are some problems in implementing the guidelines.

Problems are everywhere. Medical superintendent, officers have direct touch with minister. Though there are problems, we have to work.

There is a separate study of hospital management. He assists medical superintendent, gives presentation. But the post is vacant as no one responded to the advertisement.

**I: What may be the solutions to these problems?**

R: Everyone should put their helping hand for the management of waste and manpower should be supplied.

**I: How is the hospital doing MIE of healthcare waste management?**

R: There is a committee for this. It evaluates the workings of indoor incharge, emergency incharge and sweeper.

**I: Is there system of using checklists, log books in recording the production and disposal of waste in hospital?**

R: It would be done if manpower is available. But there is no manpower so it is not done.

**I: How can the health of the workers of hospital such as healthcare waste handlers, sweepers can be secured?**

R: We can’t say anything on this.

**I: There are sweepers in hospitals. What do you understand by work related health?**

R: Hospital development committee has recruited the sweepers but the committee has no money. It is difficult to pay money to the sweepers though they are working in minimum salary.

**I: How can they be protected from hazardous and infectious healthcare waste?**

R: Personal protective equipment (PPE) can be provided but their growth cannot be considered.

**I: We are focusing only in health not in social aspect. Things you have said belongs to social aspect.**

R: We are providing PPE such as masks, gloves in daily basis possibly. Training on waste disposal like use of different colored buckets or dustbins for different types of wastes is done once a year. However, sometimes lack of manpower created some problems.

**I: That is a good thing. Is there any positive changes can be seen in sweepers after providing trainings and CME?**

R: Yes, there is

**I: What type of effects can be seen?**

R: From the training, participants are able to understand how the waste can be managed and proper way of disposal of wastes. Regular training reminds the workers in proper management of waste.

**I: How do you prepare annual plan for the management of healthcare waste in the hospital?**

R: Annual plan is not prepared. But we are managing wastes daily. It would be better if we prepare daily plans.

**I: Whom will you include in preparing annual plan if you have to make the annual plan for healthcare waste management in future?**

R: If so we will include emergency incharge, heads of all departments, indoor incharge, medical recorder, lab incharge, administration and finance head.

**I: What will happen if sweepers are included in annual healthcare waste management plan? And how much important is it?**

R: it is important to include them in preparing the annual plan. It is because they will understand working system and there will be pressure for them to know how to do.

**I: While preparing annual plan, how much it is important to make separate budgeting, define responsibilities of the workers and time table for buying?**

R: It is a very important task after forming committee.

**I: Is there any awareness program held to aware healthcare waste handlers?**

R: yes, awareness is organized in regularly. And it is important.

**I: How can you make healthcare waste handlers (Sweepers) to participate in district hospital and national level?**

R: We have prepared criteria in district hospital. But we don’t know about national and province level. Planning must be done by related stakeholders.

**I: National training center is organizing various trainings each year. How will it be if they prepare schedule of training on healthcare waste management for waste handlers?**

R: It will be very good if it happens.

**I: Is it possible to organize trainings by hospital itself by hiring certain consultant?**

R: We don’t have capacity to hire consultant. However, our indoor incharge gives training each year.

**I: How much decline in production of waste can be seen because of awareness?**

R: Awareness brings decline in production of healthcare waste. Through awareness and training people understand the waste handling and managing techniques. Wastes can be recycled and reused.

**I: How can the healthcare wastes can be managed properly?**

R: Malangawa hospital lies in the center of the market. Lack of space and proper delivery are the problem in managing healthcare wastes. If it is burnt, smoke will affect the market. If a suitable public land can be used, healthcare waste can be managed properly.

**KII 5**

**I. What are the major types of hospital healthcare waste generated by the hospital?**

R: We have started operation procedure in the hospital thus bloods mixed waste are one of the major wastes generated. Also, chemical waste from the lab, food wastes, gloves, needles and syringes from the wards and waste water from the bathroom are the waste generated.

**I: What are the general healthcare wastes generated from the hospital?**

R: Gauge pieces, syringes are the general healthcare waste generated.

**I: What are the hazardous healthcare wastes generated?**

R: The chances of contamination from the operation of patients with communicable diseases like HIV and the infectious pus of the patients are the hazardous wastes.

**I: What are the issues related to healthcare waste management in the hospital?**

R: There are a lot of issues related to healthcare waste management in district hospital. There is no proper healthcare waste management strategy. But this time, social development ministry of province 2 has allocated budget for healthcare waste management. Thus, healthcare waste management plan is in pipeline. We aren’t allowed to burn the healthcare waste which we used to do before. Also, the placenta pit is also filled and we don’t have budget for new placenta pit.

We don’t have any autoclave for the waste. We do collect healthcare waste separately but are disposed all together only.

**I: Who are the persons responsible with waste management plan?**

R: Medical superintendent is the primary person responsible with waste management plan. Besides, staffs of waste management, hospital management team, ward staffs, nurses, emergency staffs, paramedics, waste handlers are also equally responsible.

**I: What is the condition of the specific planning implementation? Is Healthcare waste management a low priority for the hospital?**

R: Health care waste management is always a concern of priority to the hospital. Proper waste management helps us to avoid the transmission of the infectious diseases. Also, the control of the pandemic is related to healthcare waste management. But we are always in budget inefficiencies for healthcare waste management. This is the first time the provincial government has allocated budget for this purpose. We are not able to work efficiently in this sector.

**I: What are the major issues and challenges that is hindering implementation of National Standard of Waste Healthcare management?**

R: We are in lack of manpower and budget to implement healthcare waste management guideline 2014. We do not have autoclave. Also, we are now here near segregation of waste as well as reuse recycle of the waste.

**I: Is there any problem related to staff communication, monitoring and supply of the material?**

R: Yes, definitely these issues have significantly affected us in every sector. But in case of our hospital amid of minimum resources we are giving our 100% to the work thus this is not a main issue here.

**I: How do you monitor the healthcare waste management of the hospital?**

R: We don’t have any guideline for the waste management nor we follow the national standards. We the management committee head and me myself monitor the hospital and look after the healthcare waste management.

**I: Do you have any checklist or logbook to keep the record for healthcare waste cleanness and disposal?**

R: No, we don’t have any checklist or logbook.

**I: How do you ensure occupational health of the waste handlers?**

R: We cannot afford any of the measures for occupational health safety. But we do expect our government to allow insurance to the workers as well as free checkup to the infected workers.

**I: Do you provide PPE, masks, gloves to the waste handlers in the hospital?**

R: We can provide PPEs, masks and gloves to the waste handlers. But the main issue we need is proper training and awareness program.

**I: How important is training and awareness program to the healthcare waste handlers?**

R: It is very important to conduct training and awareness program to all the workers in the hospital from the superintendent to the sweeper staffs. We do have guidelines but cannot implement anything. Thus, everyone should get knowledge regarding waste management and the proceedings. Also, behavior change and the disciplines of the top to bottom workers is equally important.

**I: Do you have annual planning's?**

R; No we don’t have any annual planning.

**I: How do you utilize the strengthening funds (Suddhirikaran)?**

R: We don’t have any funds this year. We all the staffs collect certain fund and buy essential equipment like dustbins for waste management.

**I: How important is the training and awareness programs for waste management?**

R: It is very important but we don’t have adequate knowledge and awareness. We do have documents but never implement that. Everyone should follow their duties accordingly and should be active in their own work.

**I: How do you include waste workers in healthcare waste management training?**

R: We do include them in all the works in healthcare waste management training.

**I: Do you think the government should schedule trainings to other diseases as well?**

R: It is very important to schedule trainings to others diseases for all the workers from medical superintendent to waste workers. Superintendent is the main responsible person for healthcare management and workers occupational health safety thus training is very important for them as well.

**I: How do you relay the awareness campaign to all the workers and also review the training campaign?**

R: Only one nurse get training in a year. And when she gets transferred, no one in the hospital has any idea about the campaign. Thus, every staff should equally get training and awareness program. The cost is somehow equal on comparing with TA, DA, and other allowances.

**I: Can the hospital provide training by hiring the consultant for 2-3 days for half days?**

R: It is definitely possible as well as a good idea that include all staffs in the training.

**I: What do you think is the role of three level governments in the waste management?**

R: Central government should at least provide training to all the workers of the hospital.

Our hospital is under the provincial government. Thus we expect allocation of the budget from the provincial government. Also, the provincial government should allow managing proper staffs and manpower for the hospital.

The local government transfers the fund of for the health care management to other sectors. We don’t expect that from them. Also, the local government only provides us vehicles for waste management. Thus we have very low expectations with them. The mayor and the local government officials should also be given awareness program on hospital waste management.

**KII 6**

I: What is the healthcare waste generated from district hospital?

R: Waste is related with the people visiting the hospital. Cotton, gloves, instruments, placenta during delivery, sharp instruments are generated from hospital. Some waste is produced during sterilization.

I: What do you mean by general healthcare wastes?

R: Papers, various drugs we use, tissues, gauge piece, cotton, clothes that we daily use which are not disposed properly are general healthcare wastes.

I: What are hazardous and infectious wastes in hospital?

R: Infected urine, sputum, and placenta are the source of infection. Sharp instruments are also hazardous. Waste generated from patient, lab, delivery and OT. Various wastes such as plastics, sharp instruments, and infected wastes are generated in lab.

I: What are the issues related to waste management in district hospital?

R: In case of waste management cleaning is very less in the hospital. And proper disposal,

Creating safe site for waste management are also a big issue for hospital. We are planning open tender for waste management in the hospital.

I: Dr. Bipin, there are six steps in healthcare waste management like segregation, transportation, annual planning. Are the issues related with these?

R: All issues are there such problem in transportation, lack of proper site and specific planning. We will open tender for all.

I: Who are responsible persons related with these issues?

R: All the persons including visitors, patients, and staff should be responsible. One cannot solve these problems even he is head. Everyone should help to reduce waste. Staffs and patients together can and must reduce waste from their side.

I: Is mayor of the municipality responsible person in waste management?

R: If municipality collect deposited waste in time it would be easier managing waste.

I: All organizations including district hospital lie inside a municipality. So, are these the issue of concern for municipality?

R: Yes, it is. If the hospital is clean, people of the municipality get benefited.

I: What are the problems in implementing health care waste management directory 2014?

R: There are two things: policy making and implementation. If policy cannot be implemented properly it creates problems. I have been looking for healthcare waste management but budget is recently released. In governmental hospital processing is very slow. Budget and procurement, ill mentality towards waste management are also problems. Also there are problem in tender.

I: Have you felt problems in attitude?

R: Yes, there is. If staffs shows positive attitude and take responsibility it will be easier in waste management.

I: What can you do to solve these problems?

R: First we should do counseling and go for punishment. But government must understand that the salary is very low which demotivates staffs in managing waste is. Attitude is very poor towards waste management. It will certainly change in near future.

I: How can you monitor and evaluate waste management in district hospital?

R: In my opinion, there should be separate department for this. One supervisor should be recruited so that he can monitor and evaluate weekly or monthly and report.

I: What about meetings and other?

R: It is very important to organize meetings to mitigate waste management problems because patients will get affected if hospital is not cleaned.

I: Dr. Bipin, Is there system of making checklist for monitoring sweepers?

R: Yes, it can be done. Training on using different colored buckets for collecting different type of wastes can be given to sweepers. I think municipality should provide such trainings to aware sweepers to understand and use different buckets for plastics, infected urine, degradable wastes etc.

I: Can a logbook be put to examine the daily waste production?

R: It is important to monitor management of waste rather than examining waste production.

I: It would be better if checklist is prepared to monitor waste.

R: Yes, it is.

I: JN: How can you ensure occupational health safety of hospital staffs especially sweepers?

R: Sweepers are at high risk zone in hospital as they have to deal with management of various infected wastes such as syringes. They are exposed to waste containing various contagious diseases such hepatitis B, hepatitis C, HIV etc. So they are provided with universal precautions such as masks, gloves. If anything happens to them, faculties should be managed. System of using various buckets for collecting sharp wastes, gloves, plastics should be implemented to minimize injuries.

I: Some may be aware of PPE and some may not. Despite use of PPE staffs may get injured. What can be done for post prophalaxis?

R: Government should occupational health safety of the staffs and take responsibility if they get injured.

I: You mean government should provide insurance for injuries. For some injuries, contaminations and exposure to various diseases, hospital itself can provide vaccinations and treatment to injured staffs, can’t it?

R: Yes, hospital can do these. But, I mean government should also support in these conditions and provide medical insurance.

I: Have you prepared annual plan? If yes, how do you prepare?

R: I have worked in PHC before. I haven’t prepared annual plan till now.

I: Why do you think trainings and awareness is important for healthcare waste management?

R: Unhygienic environment in hospital may transmit various diseases to patients and staffs. So hospital must be cleaned. Dirty environment in hospital creates negative impression on patients as well. To keep hospital clean and healthy, all the staffs should be trained how to dispose waste properly.

I: How can you make cleaning staffs participate in healthcare waste management?

R: Cleaning staffs must be include and separate training should be given.

I: Government has prepared schedule in their policy. How will it be if sweepers are provided with healthcare waste management schedule?

R: It will be better because most of them do not have idea. It is included in curriculum of MBBS and MD as well. It will be better if training is given and implemented.

I: How do trainings and awareness programs help reduce healthcare waste management?

R: Trainings and awareness provide knowledge of dealing with wastes and segregating at site to staffs directly dealing with waste such as sweepers, lab technicians and health workers. It will increase awareness of using different buckets for different wastes.

I: As a medical superintendent, what will you recommend all level of government (federal, province and central) for proper management of healthcare waste?

R: First of all, change in attitude towards waste should be brought among people. In Terai, tobacco is commonly used which is one of the major source of waste. People using tobacco should be told to dispose waste properly. Also, dustbins should be put in various locations for collecting wastes. Government should sensitize staffs and general public through trainings, awareness programs, lectures, and demonstrations. Proper disposal site should be made.

Autoclaving should be improved. Proper budgeting should be done for waste management. Hospital should open tender for managing hospital waste. Time to time assessment should be done. All level of government should workout in managing waste. Awareness is the most important task should be accomplished. Then, we should put our hand on waste management.

**KII 7**

I. What are the major types of hospital waste generated by the hospital?

R: Major waste are generated from the wards while treatment procedure like bottles, canula, foles, catheters bandage, gauge and other medical waste. Also, paper and plastic bottles and biological materials, placenta and OT wastes are major waste of the hospital. Thus can be categorized as general waste and hazardous wastes.

I: What are the issues related to waste management in the hospital?

R: The first problem is related to collection of the waste. The number of flow of patient directly increase waste generation. Also, large proportion of waste is generated by patient parties and visitors .Also, other issues are related to management and proper disposal of waste by patient and patient parties. Another problem is related to lack of proper manpower. We don’t have adequate number of sweepers. Also, segregation of waste, lack of awareness of disposal of waste and other waste disposal plant is also issue here.

I: What is the factor that is hindering the implementation of healthcare guideline?

R: There are two three factors related to this. FIrst is the timely training that must be given to the workers and sweepers to guarantee skillful workers. Other is effective working manpowers and checklist of work division is also hindering effectiveness of guideline implementation.

I: How will this affect the procurement by the management?

r: We have been providing equipments like phenol and harpics for the workers for proper health care management .

I: Who are the major stakeholders related to waste management?

R: FIrst responsible person are patient and patient party. If they manage waste efficiently half of the work is done. Secondly, sweepers are also responsible for the duty responsibility. Other than different wards and sections of the hospital are also equally responsible for waste management. For waste management we can council the patient and their parties. For this the hospital can appoint a focal person.

Also, store keeper, management are also responsible for providing equiipments in time. The head of the hospital is also responsible in providing proper working environment for the workers.

I: Why cant the hospital implement Healthcare waste management guideline 2014?

R: I am unaware about the guideline thus cannot speak about it.

I:How do you monitor the waste management of the hospital?

R: We have formed the committee which look after the waste management of the hospital which time and often inspect the hospital and order the staffs about the status.

I: Do you have any checklist or log book for monitoring?

R: No, we don’t have any checklist for monitoring.

I: How do you ensure the healthcare facility of the waste worker staffs?

R: We provide them with boots, glovers and masks. Also, we provide timely vaccination to the staffs. In case of exposure medical procurement is also practiced.

I: How do you plan annual plan for waste management?

R: We don’t have any annual plan in waste management sector.

I: Do you think trainings are important for waste health workers?

R: Yes, timely training and awareness for waste handlers is very important which ultimately helps in implementing the guidelines.

I: Is it possible to hire a consultant for waste management trainings?

R: Yes we can manage trainings but proper timing and manual should be published .

I: Is it possible to include workers in national level trainings?

R: Yes it is possible.

I: What are the roles of central, federal and local government for waste management?

R: We expect proper guidelines and routine budget for waste management. Also, training should be organized time and often. Also, helping in the recruitment of adequate number of sweeper should be managed. Also, we expect logisitic support from the local government.

**KII 8**

I. What are the major types of hospital waste generated by the hospital?

R: Basically the two types of waste generated are infected and non infectious waste which are generated from different sectors of the hospital like different departments, OPDs and Labs. Also, waste from emergency and indoor dressing. Non infected waste includes food waste from patients and also bottle waters and also gutkhas while infected included that of generated from emergency, O.T., delivery rooms and labs.

I: How do you categorize waste from the pharmacy?

R: Basically pharmacy waste are non infectious which may be hazardous as well as non hazardous like cartoons, expired medicines and so on.

I: : What are the issues related to waste management in the hospital?

R: We do have waste management team of 4-5 peoples. We do have issues with transportation and disposal sit. We also lack coordination with local government for waste management.

I: How do you plan waste management?

R: We plan waste management in 1or 1 and half month interval through meeting by waste management team.

I: How do you prioritize health care waste ?

R: Heath care waste management is neglected by most of the hospitals due to budget or implementation crisis. But, we have been doing well I guess. Like we have built 3 room building for waste management. we don’t have a concrete plant but we are planning it for upcoming year. We do have buckets for different waste management and different chambers for different waste collection. We are planning to generate gas from waste in upcoming year.

I: What are issues related to monitoring and evaluation supervision?

R:We do have health care management committee including different staffs and nurses. We do have incharge for monitoring and evaluation which is also inspected by municipality and hospital administration.

I: What are the issues related to equipment's for waste management?

R: we do have budget allocated for waste management which allocate amount for equipment.

I: Who are the responsible stakeholders related to waste management?

R: Medical superintendent, sweepers and waste management committee are the main stakeholders related to waste management.

I:What is the role of the community?

R: Municipality allocates certain budget for the hospital Else where, there is no role of the community. Municipality is also lacking coordination as the are yet to provide vehicle for waste collection.

I: What are the role of the people for hospital management?

R: If the people help in segregation of the waste from the source, it would be lot more easier for waste management. But we do lack awareness in local level in this subject.

I: What are the issues related in implementing Health care waste management guidelines 2014?

R:First of all the issues is of the manpower. We only have 3 sweepers who work on shift basis thus segregation of the waste is not possible. Also, there is lack of budget to implement guidelines. We don’t have high voltage electric supply, regulation and motivation among the staffs.

I:What is the problem related to communication problems between waste workers and management?

R: We don’t have any kinds of problem related to communication since this is a small hospital and meeting is held every month.

I: How can you implement the waste management guidelines?

R: Motivating the regular staffs, routine meeting and providing rewards to the workers and allocating budgets would help in implementing waste management guidelines.

I:Who are responsible for monitoring and evalutation?

R: We have a team of indoor incharge as the chairman, each workers from each of the department as the member where the chairman guide each department and members for monitoring and evaluation and flow tree is also formed for proper evaluation and communication.

I: Do you have checkbooks or log books for monitoring?

R: We do have checklist for the sweepers who mark the checklist after every duty completion while we don’t have any log books and checkbooks for waste management.

I: How do you ensure healthcare workers safety in the hospital?

R: Sweepers are on high risk from the waste management thus we provide PPEs, masks gloves and also provide guidelines for treatment incase of any sort of exposure and T T injections are timely provided.

I: How do you plan for annual plan for waste management?

R: The plan for waste management is formulated by waste management committee which is further minute and is taken on action. In case of the major plans, we further contact with the municipality as well as the provincial government for implementation.

I: How do you manage training for waste handlers?

R: I guess training is required for all sectors. It would be great if we could provide training to all the staffs , sweepers management teams sothat they can learn more about the waste management.

I: Is there any provision that you can hire a consultant for waste management?

R: Atleast district hospital should be capable of hiring a consultant.

I: How will awareness help in waste management of the hospital?

R: Awareness will help in reduce reuse and recycle which will ultimately help in waste management in the hospital.

I: What do you think is the role of local, provincial and federal government?

R: Central government should help in implementing guidelines for waste management and help in the monitoring of waste management while local government can help in logistics and budget allocation .

**KII 9**

I: What are the different types of health-related wastes generated in the district hospital?

R: Yes, we can talk department wise or we can talk category wise. We can talk according to you. It is easier according to the department.

Yes there are needles, blood mixed cotton, there are also some waste thrown by the hospitalized people and plastic waste materials.

I: That means ordinary health waste is paper, plastic, and so on am I right?

R: Yes, that's right they might have selected form delivery room.

I: That means placenta is general and hazardous waste, another which is generated from delivery room. am I right?

R: Yes, there are two such things. Hazardous and non-Hazardous. they are also producing.

Likewise, if we talk about the category of Liquid waaste and hazardious waste are also one of them.

I: Ummmm. In the same way, now I want to move a bit forward, what you want to say about the issues that are filed in district court?

R: Yes, Waste management. Yes, we are doing segregation but other things like blood mix and they need to autoclave, we are not able to send them. We separate the waste and for that out province government also separate the budget for it. We used to do that but we are not doing it right now. And others nonhazardous waste is given to the municipality.

I: That means you have an issue with health care management. Like how the segregation was happened?

R: After the segregation we have an issue with disposal. Likewise, there is a problem in center collection side, municipality is helping you but they are helping only for non-hazard sample waste.

I: These are the issue of yours, did you face any issue in the hospital? You have the excessive planning, annual planning and healthcare waste guidelines for health and waste management. But these planning are not implemented any many places or health care waste is not a prior topic for them. Have you face any kind of issue like this?

R: No, we haven’t had any issue

I: We just talk about the issues connected to them. Who is the responsible person for this?

Who should I blame, Responsible person are committee member? Did they help in management of waste or not?

R: Sick people do not have a proper knowledge to throw which waste to where. Some issue is arising because of our committee member and their role in our organization. May be due to this reason our province government separate the budget of waste management and hopefully we can do our best.

I: Ok this much for now on this topic, this topic is connected to the management, what are the issues while implementing the waste management guideline and how to solve this issue?

R: We have done how much we can but till when we have our segregation kit, we cannot manage those issue. We should start from there; we should know which waste should throw where. We called 90%, what we called is they are those waste which came in municipality truk. That is what we decompose and 10% are hazardous and it was blood mixed. And we are in risk, we install autoclave there and we decompose.

I: And the thing which are connected with the guideline, do you have something more? Like you may have problem with monitoring or you may have problem with following the guidelines or to buy goods and product or you have the problem make this kind of community?

R: The thing is we are not able to convince people.

Yeah, that’s right, that means you have an issue with implementing the guidelines, So, what di you that, what are the solution to solve this kind of issue.

This waste management is a big challenge for the hospital, to solve this issue our Chairperson, staff, and committee member should actively help us.

I: Like you said to solve this issue every employee and committee member should proper coordinate, right?

R: That can't be solve with segregation, now it needs to autoclave, we needs to disinfect then it can help, we also need setup for that and our province government municipality are working for it, proper setup might help otherwise it is not possible.

I: What do you think, how waste management monitoring should be done?

R: This is the challenging thing in waste management. We are monitoring but this is not effective. We are giving training time to time and UNICEF also provide training related to segregation, waste management but it is really difficult to manage. As a chairperson we monitor these things but I personally think it is not effective till date.

I: We called it occupational health and safety, so, those employees who are working for waste management, what do you think how they can secure their health?

R: Firstly, we are giving them a knowledge about hazardous waste, also we told them about the problems. They are taking universal precaution while they work, we followed them, also we are accepting them.

I: How to use

That means you are giving awareness program, you aware people about how to protect form hazard, also aware people about how to wear and PPE. And furthermore, if someone cut them up with needle/ Hept B pin what would you do?

R: Till now we haven’t seen these kinds of issues, but if we had these kinds of issues, we will go for a treatment which was from Hospital side. And in the hospital, they should establish the Vaccination program, TT, Hepatitis B Vaccine.

If someone suffered with HIV then, what do you think about the medicine if you can get those from the hospital itself.

that would be good if we could get that but till now this service are not served. But There is nayarani hospital, you can contact them if you need medicine. we have a good relation with them. They are for safety if needed for medicine they would contact and ask for medicine.

Yes, medicine is available there, You may not get every medicine but there are HIV medicine available and for Hepatitis B medicine you have to visit Kathmandu.

Hospital does not need development group but it would be great if government takeover them? umm Yeah it is good if the government would take. government should take responsibility of health workers. For example, we are doing work in pandemic, if someone work in the time of corona pandemic, government should provide allowances and 75% to 50% extra salary is provided in different places. These kinds of things should be done by our government.

I: If you get a chance to make an extra law and yearly plan, what would you do to make Health related waste management plan in your hospital?

R: Yes, we have our own Redbook where we have a guideline on and according to the guideline, if hospital get a Yearly budget categorization, we have somehow managed 15% fund to spend on waste management. Like if our workers want to buy some goods e.g., toilet cleaner finel, harpic, brush etc. it was included in that 15% budget.

I: In the time of making yearly planning, who do you think to get involve with you?

R: While we make yearly planning according to the government norms, we have 15%. And another one is Healthcare waste management guideline, to make this guideline hospital recruit some staff to make the guideline about the healthcare waste management.

I: if you create the guideline, we can include 2 things in one and we can create healthcare waste management annual planning. how would you make the guideline?

R: Likewise, we haven’t done meeting, haven’t planning yearly planning, we discussed in committee meeting, in the committee meeting we have medical officer and members from another department and we discussed in the meeting related to waste management. It would not take aggressively, then we will do according to you.

I: If it is not made yet then we may have a guideline on how to create health care waste management guideline?

R: We will follow up this guideline.

I: Why health-related waste management related training and awareness program are important?

How we can train people who are working in the hospital?

R: Till now we are getting training by UNICEF, their training is good and effective also Employee who are working on waste management are down level people. that’s why they have an important role because they are active. By doing work if they are lack of education then we should help that how to handle everything. if we don’t let them learn then they might not work, they might face difficulties and they might have lung cancer that’s why they need training.

I:How we can engage everyone?

Theoretical training is not effective. so, we need a practical training through which we can demonstrate the trainee that how to do the things. I don’t know about everyone but I think workers, in charge, including nursing staff should active in waste management. It would be good if we could include them in the training.

I: My personal curiosity, what if national central government schedule and organize some training related to nutrition and waste handling?

R: it would be good

I: Another thing is in the local level consultant they rise and like you belong from pokhaiya municipality. so, if your municipality rise some consultant and also there are others health facilities. Also, you have your waste handlers' team, they also discussed about hazardous and non-hazardous. Shouldn’t they hire a waste consult and give training related to waste?

R: We can do that but that depends on the activeness of municipality.

What's your opinion on what Nepal government need to do with waste management with the help of federal government, municipality, province government? What do you think that these 3level government need to do?

R: I already stated that waste management is a big challenge and we need a good budget, and we also need big space for that and we need to have an experienced officer staff. So, we need a good manpower. Those people who worked actively for department in any shift that segregation, this is what I am seeing right now. While I am talking in the context of my hospital those staff, who work in the morning shift, we need to make a kind of law that we need budget for waste management. In the context of our hospital our municipality has budgeted of 25% for waste management which is really a good thing to do. We need to buy auto lay and for that we need a big space and we can do segregation and even we can allocate people. That’s why many hospitals have done segregation only once and people are less after less. So, darbandi needs to increase. By making the budget they need to launch an awareness program in local level, after training they might help in segregation. We should aware them not to throw waste here and there. Local level also has their own budget. So, they can do by themselves. Major issues are we need to have a good policy, and furthermore we need to have a resource. We need more employee for motivation program and training. After doing these thing waste management should properly do.

**KII 10**

I: What are the different healthcare wastes produce in district hospital?

R: Healthcare wastes are degradable and non-degradable. In our emergency department healthcare wastes is cotton swab, urobag etc. In MCH, cloths and water which is produce during delivery time. In lab, different chemical and in the OPD side waste papers are more.

I: What do you mean by simple healthcare waste?

R: In simple waste, different waste paper, cotton swab etc. People who came in hospital for different services, they also leave different waste. In health related waste, saliva, blood, other liquid solution which is discharge from patient body during procedure and cotton swabs.

I: You already told this, Hazardous wastes are produce from where in the hospital?

R: Hazardous waste includes those medicines. Which are expired, chemicals use during preparing the slide in the lab, sputum and specimen container, blood , urobag, Betadine during giving insulin, during I and D. We use different equipment, including dressing. From MCH, Placenta and equipment used during delivery. These all are infected waste. This create problem when we contact with them. These are hazardous waste.

I: what are the issues relate to hospital waste management?

R: health personals are well known about which waste is harmful or which is not. But the personal works in waste disposal, they do not know about this. They think all waste are as a general waste. So, waste to dispose waste according to segregation protocol (green, yellow, red). But due to same misunderstanding or unable to maintain segregation protocol, waste management not done properly and this is being one of the arising issues in hospital waste disposal.in the other hand , being health personnel also while working and managing hospital waste , there is presence of negligence. Person assigning for managing waste disposal should be given counselling, training regarding waste management. This can be effective and can enhance the management of hospital waste.

I: other issues like transportation, side of disposal, planning…

R: Not at all because I think main important is given to sanitation. In every programm, activities planning are given important, procurement is also done at time. Next one is monitoring is done and is being effective. I think this might be done due to lack of training for monitoring. Persons doing monitoring might have confused and less knowledge regarding monitoring, lack of understanding due to temporary placement. Most of them are not permanent and posted for only one year. Because of uncertainty in this post , this problem has been raised.

I: side by side, when we do annual planning to run hospital is their specific planning for waste management. What do you think?

R: there is a hospital strengthening program in hospital under which we plan for waste management activities. One nurse in-charge is head and under her waste management planning is done. Issues regarding waste management usually come from outside of hospital rather than us. As there is collection of waste, issues has been raised.

We do planning of waste management and according to that planning we have arranged everything. We had chosen and made disposal site also and these all activities comes under planning of waste management.

I: ok, there are all, similarly there is lack of trolley to transport waste. In some place there is no facility of road, waste are transport through the way from where patient travel. What is the condition already used in your hospital.

R: we have enough trolley for transportation. There is a dumping site (back side) of hospital. Waste are not transported through patient way as there is issues regarding this, we keep waste separate like health waste are kept separately and other waste are kept.

I: And what about storage, disposing of waste in your hospital, infected waste most autoclave before dressing it. What is the condition about it in your hospital?

I: what are the roles of municipality waste disposal?

R: Municipality itself manage waste disposal, less amount of budget was separate for waste disposal management through this budget separation, waste management is much better than previous.

I: what kind of problem face by district hospital to implement waste management guideline 2014?

R: we are trying to do as per waste management guideline 2014 and it is implemented as a part. We are arranging all things that are needed for waste management and procurement of all that things needed for waste management. We have all articles and that are sufficient for waste management we did all things for implementing effective waste management. These who are non-health person have little bit difficulty during waste segregation for waste management.

I: how do you identify difficulties in waste management?

R: we have one group for that we do group discussion 2- 3 times per year. Some time we do discussion with medical superintendent and staff of other hospital. We provide guidelines for what and how to do waste management for these and its improvement.

7-8 photo

I: Except waste management what you do?

R: we have some sweeper about good waste if any difficult arise then let the sister in-charge know, we arranged waste management program and said things any difficulty, let know about it.

I: how do you monitor and evaluate waste management in hospital?

R: Nick simon monitored and evaluated the waste management process. It is one of the part of waste management along with we also monitor and evaluate. As we have a general command waste management, activities going as per command.

I: how you assigned focal person for waste management in hospital?

R: we all one responsible for waste management in hospital. So, we all supervise that waste management process. No one has assigned for monitoring, In our hospital one sister observed the waste management process. But no one has officially assigned for that jobs.

I: Have you formed committee that consider control of sister. Sweeper and staff from emergency work under head sister in this way we make committee for healthcare waste management.

R: yes, we have formed committee that is consider control of sister, sweeper and staff from emergency work under head sister in this way we make committee for health care waste management.

I: have you prepared tool for healthcare waste management?

R: we have not prepared tool for this. This should have been done very early. We will arrange it soon.

I: how can you ensure the occupational health of healthcare waste handlers?

R: for this, they need personal protective equipment. Previously, they have been given and using equipment like masks, gloves, boots, etc. Currently, due to corona Virus they have been managed and given at least one PPE> they are being told to use boot to mask, face shield to protect oneself. After complete PPE only, they start to work, they are being instructed this way. In other places also, it would be effective this way. Especially, they are in contact with blood and body fluid.

I: Incase, if something happened to employee then, what are should government do to manage this?

R: Government should manage facility of health insurance to them. Employees are on the basis of daily wages, if health insurance facility is provided then, they would be motivated towards work. This would be early and getter.

I: Along with awareness program, do you also provide CME program?

R: Yes, there is problem regarding CME programme. We are trying to solve it.

I: who and how plans healthcare waste management yearly?

R: we have hospital management committee. In this committee, there are manager, me (MS) and nursing in-charge. After that, we committee and other staff from hospitals sit together and discuss what is good for waste management.

I: what kind discussions are made in yearly meeting for improvement of hospital waste management? Are there discussion regarding budget, waste disposal, equipment and material?

R: During meeting, we discussion about equipment and materials that are needed because these programme is continued from many years. Then, we observe out stock equipment and materials, if some materials are needed then we manage according to our budget. In previous year, we got some extra budget from province government. From this budget, we purchased some needed equipment and materials. Now, we are doing in a same way.

I: Who are responsible person for budget management?

R: …..

I: why are training and awareness program important for healthcare waste management?

R: Training and awareness are very important. Staffs get information only through training and awareness program and they gave motivated through this. After that they will know about problems relates that arise during waste management from person to hospital training and program helps to motivate employee and it is very important.

I: how can you include and participate every cleaning staff in this training programme?

R: This training programme is very important in cleaning staffs. To ensure participation of every staff, training program should be conduct frequently (2-3 participants at one session)

I: Nepal government is conducting various programme and training such as SBA training. Is there any training related to waste handlers by Nepal Government.

R: waste handlers training can be schedule and conducted by government of Nepal. Protocol can be developed and managed. We should also initiate some steps for this.

I: Can hospital hire a consultant to provide training?

R: In case of district level, we can do it. But in our own district level hospital there is lack of sources and equipment. But, I think we can conduct it in our district hospital.

I: how these awareness programme help to decrease waste production?

R: Mainly waste management are in two parts. They are recycled and reuse. If we could do this then it would be very effective it helps in less waste generation and it helps to use waste in a productive ways.

I: if you are given chance to give suggestion to government regarding waste management, then what kind of suggestion would you give?

R: if I get chance to give suggestion to government regarding waste management, then I think a hospital should appoint permanent person who will be responsible for waste management if these is temporary employee then, we have to explain provide councelling regarding protocol to them time and gain only the waste will be managed properly. Sweeper should also be permanent employee aand should be known about protocol of waste management, while working there would be nursing icharge. I and other staffs to instruct them for this. Hospital should appointee healthcare manager for effectiveness.
